# Induction of Methuosis in Breast Cancer Stem Cells by a Cobalt(III) Complex Evokes Immunotherapeutic Response

**DOI:** 10.1002/anie.8926368

**Published:** 2026-09-10

**Authors:** Jiaxin Fang, Sijie Wen, Xiao Feng, Hao Ren, Kuldip Singh, Haihua Xiao, Lingpu Zhang, Kogularamanan Suntharalingam

**Affiliations:** ^1^ School of Chemistry University of Leicester Leicester UK; ^2^ Beijing National Laboratory for Molecular Sciences Laboratory of Polymer Physics and Chemistry Institute of Chemistry Chinese Academy of Sciences Beijing P. R. China; ^3^ Suzhou Institute of Pharmaceutical Innovation Soochow University Suzhou P. R. China

**Keywords:** bioinorganic chemistry, cancer stem cells, cobalt, immunotherapy, methuosis

## Abstract

Cancer stem cells (CSCs) drive metastasis and relapse and are resistant to conventional apoptosis‐inducing chemotherapies. Methuosis, a non‐apoptotic form of cell death, is largely unexplored for anti‐CSC therapy. Here we report a cobalt(III)‐cyclam complex (**1**) incorporating pyrene and diflunisal moieties that induces features consistent with methuosis in a CSC model. Mechanistic investigations revealed that **1** induces phenotypes consistent with methuosis in breast CSCs, including extensive cytoplasmic vacuolation, elevated Rab7 and LAMP1 expression, activation of JNK and p38 MAPK pathways, G2/M cell‐cycle arrest, augmented intracellular reactive oxygen species levels, and disruption of the mitochondrial membrane potential. Beyond direct cytotoxicity, **1** induces an anti‐breast CSC immunogenic response by perturbing the inhibitory damage‐associated molecular patterns axis through cyclooxygenase‐2 downregulation. When formulated as polymeric nanoparticles and evaluated in a murine metastatic triple‐negative breast cancer model, **1** penetrates tumour tissue and suppresses tumour growth more than cisplatin. The nanoparticle formulation activates anti‐tumour immunity, characterised by enhanced infiltration of T cells and mature dendritic cells in tumours and tumour‐draining lymph nodes. Intratumoral T‐cell recruitment is associated with M2‐to‐M1 macrophage polarisation and depletion of myeloid‐derived suppressor cells. This work identifies **1** as the first metal complex shown to induce features consistent with methuosis in a CSC model.

## Graphical abstract

Cancer stem cells (CSCs) drive metastasis, relapse and resist conventional apoptosis‐inducing chemotherapies. We report a cobalt(III)‐cyclam complex with pyrene and diflunisal moieties that reduces the viability of breast CSCs at low micromolar concentrations. This cobalt(III) complex represents the first metal complex to induce features of methuosis in a CSC model.

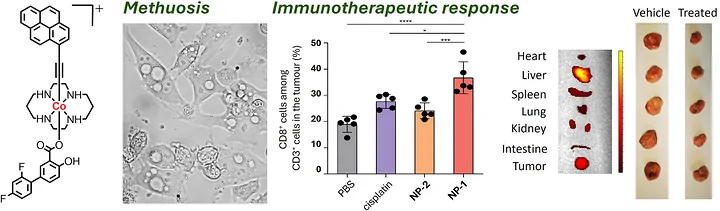

## 1 Introduction

Most small molecule anti‐cancer agents kill cancer cells by apoptosis, a regulated form of cell death [1, 2]. The widespread use of apoptosis‐inducing chemotherapeutics has contributed to higher incidences of apoptosis resistance in tumours [3, 4, 5]. Apoptosis resistance is closely associated to the inability of some small molecule chemotherapeutics to protect patients from tumour recurrence and metastasis [5, 6]. Over the past two decades, numerous non‐apoptotic cell death pathways have been characterised, including necroptosis, pyroptosis, autophagy, ferroptosis, cuproptosis, paraptosis and oncosis [7, 8, 9, 10, 11]. Methuosis is a distinct and poorly understood form of cell death, characterised by dysregulated macropinocytosis that drives extensive vacuole enlargement and subsequent membrane disruption [12, 13, 14]. The cellular machinery underlying methuosis is thought to operate independently of the apoptotic pathway [12, 13, 14], therefore methuosis‐inducers could be viable candidates to overcome apoptosis resistant cancer cells. At the time of writing, there are less than thirty small molecule methuosis‐inducers (natural products and synthetic molecules) reported in the literature, of which only one contains a metal [15, 16, 17, 18, 19, 20, 21, 22]. Bis(acetylacetonato)oxidovanadium (IV) was shown to induce methuosis in glioblastoma cells (via the Rac‐Mitogen‐activated protein kinase kinase 4 and c‐Jun N‐terminal kinase signaling pathways) and inhibit their growth in vitro and in vivo [20]. The induction of methuosis by bis(acetylacetonato)oxidovanadium (IV) was determined to be non‐immunogenic [20]. The ability of bis(acetylacetonato)oxidovanadium (IV) to evoke methuosis in apoptosis‐resistant cancer sub‐populations such as cancer stem cells (CSCs) was not investigated [20]. CSCs are a sub‐class of tumour cells with stem cell‐like properties, including the ability to self‐renew and differentiate [23, 24]. Their slow cell cycle progression enables them to evade apoptosis‐inducing chemotherapeutics, which preferentially eliminate proliferating cells that are more susceptible to apoptosis [25, 26, 27]. Following their survival, CSCs can drive re‐establishment of the primary tumour mass and initiate colonisation of distant secondary sites [28, 29]. Given the adverse clinical implications of CSCs, new therapeutic regimens are needed that can remove the entirety of cancer cells, including CSCs for long‐term beneficial patient outcomes. Several academic and industrial drug discovery programmes are currently underway to identify small molecule candidates capable of selectively eliminating CSCs at clinically tolerable doses [30, 31]. Unfortunately, these efforts are yet to identify a clinically viable candidate. Methuosis‐inducers have not been investigated in the context of anti‐CSC therapy. We intend to explore this knowledge gap and hypothesise that apoptosis‐resistant CSCs that possess the necessary protein machinery required for methuosis could be susceptible to methuosis‐inducers.

Immunotherapy has emerged as a clinically viable approach for eliminating both bulk cancer cells and CSCs by harnessing the patient's endogenous immune system [32, 33, 34]. The clinical success of immunotherapeutics has seen the cancer immunotherapy market size grow to over $125 billion in 2025 [35]. A common strategy for activating the immune system is to induce immunogenic cell death (ICD) in cancer cells [36]. ICD promotes the release of damage‐associated molecular patterns (DAMPs), which recruit and activate immune cells [37]. Inhibitory DAMPs (iDAMPs), such as prostaglandins, have recently been identified as negative regulators of ICD [38]. Therefore, reducing prostaglandin levels through inhibition or downregulation of cyclooxygenase‐2 (COX‐2) may enhance DAMP release and thereby promote an immunogenic response [38]. We and others have employed metal complexes bearing nonsteroidal anti‐inflammatory drugs (NSAIDs) to induce ICD in CSCs by disrupting the iDAMP signalling axis [39, 40]. In these limited examples, the metal complexes elicited an immunogenic response by inducing cell death consistent with apoptosis [39, 40]. Here we report an asymmetric cobalt(III)‐cyclam‐pyrene complex with diflunisal (a potent COX‐2 inhibitor) [41, 42], **1** that is able to induce features consistent with methuosis in breast CSCs and at the same time promote an immunogenic response towards breast cancer cells in vitro and in vivo, in part, by perturbing the iDAMPs axis. The reported cobalt(III) complex **1** is, as far as we are aware, the first metal complex to be shown to evoke hallmarks of methuosis in a CSC model.

## 2 Results and Discussion

### 2.1 Synthesis and Characterisation

The cobalt(III) complex **1** was prepared via the precursor cobalt(III) complex **2** as depicted in Figure 1A. The precursor cobalt(III) complex **2** was synthesised by reacting *trans*‐dichloro(cyclam)‐cobalt(III) chloride **3** [43] with 1‐ethynyl pyrene in the presence of KOH, in DCM:MeOH (1:1) under nitrogen (as depicted in Figure S1A). The crude product, obtained by removal of solvent, was purified by column chromatography [DCM:MeOH (9:1)] and converted to the corresponding hexafluorophosphate salt. This allowed isolation of pure **2** as an orange solid, in a reasonable yield (33 %). Then **2** was reacted with diflunisal in acetonitrile (dried over Na_2_SO_4_) with the added presence of excess silver(I) oxide. The crude product was obtained by work up with acetone and then converted to the corresponding hexafluorophosphate salt and recrystallised with DCM to afford pure **1** as an orange solid in a good yield (64%). The cobalt(III) complexes **1** and **2** were fully characterised by ^1^H, ^19^F{^1^H} and ^31^P{^1^H} NMR, infrared spectroscopy, high‐resolution ESI mass spectrometry, and elemental analysis (Figures S2–S13). Single crystals of **1** and **2** suitable for x‐ray diffraction studies were obtained by vapour diffusion of diethyl ether into acetonitrile solutions of **1** or **2** (CCDC 2521598‐ 2521599, Figures 1B and S1B; Table S1–S3). Both **1** and **2** adopt distorted octahedral geometries with the cyclam ligand residing in the equatorial positions and the axial positions occupied by 1‐ethynyl pyrene and diflunisal for **1** or 1‐ethynyl pyrene and chloride for **2**. The diflunisal in **1** is attached to the cobalt(III) centre by the deprotonated hydroxyl moiety within the carboxylate group. Overall the bond parameters for **1** and **2** are consistent with the values reported for structurally related cobalt(III)‐cyclam complexes [39, 44, 45, 46, 47, 48, 49].

**FIGURE 1:**
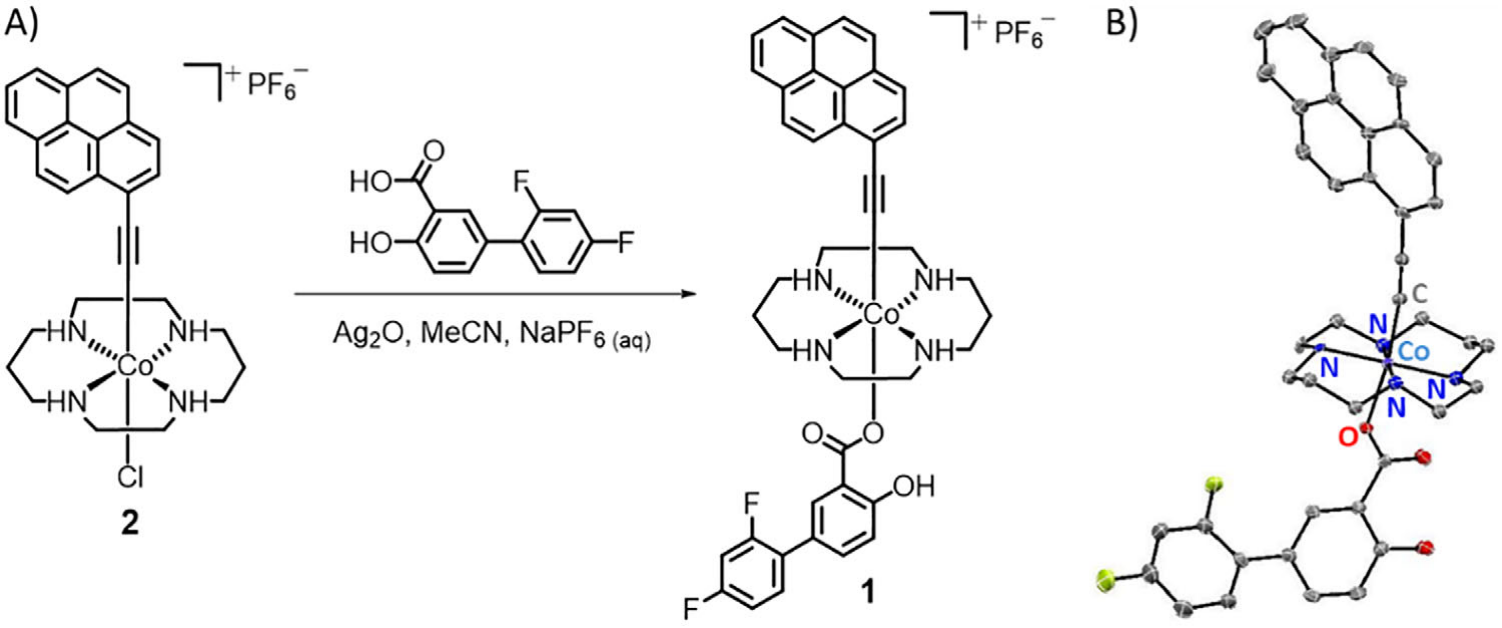
(A) Reaction scheme for the preparation of cobalt(III)‐cyclam complex **1** from **2** and diflunisal. (B) X‐ray crystal structure of **1**. Ellipsoids are set at 50 % probability level. Hydrogen atoms, the counter‐anion, and co‐crystallising solvent molecules have been omitted for clarity.

### 2.2 Structural Insight in Biologically Relevant Solutions

Aqueous solubility and cell uptake are key considerations in drug development, this was gauged by determining the experimental LogP of **1** using the shake‐flask method. The LogP value for **1** was determined as 0.96 ± 0.01, indicative of reasonable aqueous solubility and potentially good cell uptake. As expected, **2** (LogP = −1.07 ± 0.01), without the diflunisal moiety, was significantly more hydrophilic than **1**. The stability of **1** was probed by spectroscopic and analytical methods. The UV–vis spectrum of **1** was analysed in several biologically relevant solutions. The UV–vis trace for **1** (50 µM) in DMSO, H_2_O:DMSO (8:2), PBS:DMSO (8:2), and cell culture media:DMSO (8:2) remained unaltered over the course of 24 h at 37°C (Figures S14–S17), suggestive of good stability under these conditions. The aqueous stability of **1** was confirmed by ESI mass spectrometry studies. The ESI mass spectrum of **1** (50 µM) in H_2_O:DMSO (8:2) after incubation for 24 h at 37°C, displayed a distinctive molecular ion peak with the appropriate isotopic pattern expected for **1** (*m/z* = 733.4 a.m.u., [**1**‐PF_6_]^+^) and no sign of ligand liberation (Figure S18). Upon addition of excess amounts of ascorbic acid or glutathione (strong extra‐ and intra‐cellular reductants, 5‐10‐fold) to **1** (50 µM) in H_2_O:DMSO (8:2) and incubation for 24 h at 37°C, the ESI mass spectrum displayed a new signal for free diflunisal (*m/z* = 249.0 or 249.2 a.m.u., Figures S19 and S20). This implies that **1** can release diflunisal in biologically reducing conditions, likely via the reduction of the Co(III) centre in **1** to Co(II). The liberation of diflunisal from **1** in the presence of ascorbic acid was also evidenced by the presence of a signal for [Co^III^(cyclam)(pyrene)(MeCN)]^2+^ [**1**‐PF_6_‐diflunisal+MeCN]^2+^ (*m/z* = 262.6 a.m.u., Figure S19). Complementary emission studies showed that when **1** (25 µM) was incubated with ascorbic acid (10 equivalences) in acetonitrile at 37°C, the resulting emission trace (*λ*_ex_ = 356 nm, *λ*_em_ = 389 and 405 nm) was similar to that of **2** (25 µM) with ascorbic acid (10 equivalences), indicating that diflunisal had been released (Figure S21). The stability of **1** in human plasma was assessed to evaluate its suitability for in vivo applications. Following incubation of **1** (1 mM) in human plasma at 37°C for 3 days, the concentration of intact **1** remained largely unchanged, as determined by UV–vis spectroscopy and ICP‐MS analysis (Figure S22). These results indicate that **1** exhibits high stability in human plasma, with minimal cobalt dissociation or structural alteration over the 3‐day incubation period.

### 2.3 Cytotoxicity towards Two‐ and Three‐dimensionally Cultured Breast Cancer Stem Cells

The monolayer cytotoxicity of the cobalt(III) complex **1** towards breast CSC‐enriched cells (HMLER‐shEcad) and bulk breast cancer cells (HMLER) was determined. The IC_50_ values, determined from dose‐response curves (Figure S23), are presented in Table 1. The cobalt complex **1** displayed low micromolar potency towards both HMLER and HMLER‐shEcad cells. Impressively, **1** displayed 4‐ to 74‐fold lower IC_50_ values towards breast CSCs than clinically approved breast cancer metallodrugs, cisplatin and carboplatin, and an established anti‐breast CSC agent, salinomycin [50, 51]. Control cytotoxicity studies showed that the potency of **2**, **3**, diflunisal or 1‐ethynyl pyrene towards HMLER and HMLER‐shEcad cells was significantly lower (*p* < 0.05, *n* = 18) than **1** (Figures S24–S26 and Table 1) [50, 51]. Furthermore, when dosed as a 1:1 mixture of **2** and diflunisal, or a 1:1:1 mixture of **3**, 1‐ethynyl pyrene, and diflunisal the potency of the cocktails was significantly less (*p* < 0.05, *n* = 18) than **1** towards breast CSCs (Figure S27 and Table 1). This proves that the preformed complex **1** is better at killing monolayer‐grown breast CSCs than a mixture of its individual components. The cytotoxicity of **1** towards human bronchial epithelium BEAS‐2B cells and embryonic kidney HEK 293 cells was determined (Figure S28). The cobalt(III) complex **1** was significantly less potent towards BEAS‐2B and HEK 293 cells (IC_50_ values = 11.22 ± 0.08 µM for BEAS‐2B cells and 10.95 ± 0.36 µM for HEK 293 cells, *p* < 0.05) than HMLER and HMLER‐shEcad cells. Therefore, **1** has the potential to potently kill bulk breast cancer cells and breast CSCs over non‐cancerous cells, however it should be acknowledged that it is challenging to predict off‐target toxicity by simply comparing potency across a small panel of cell lines. Breast CSCs cultured as three‐dimensional spheroids (mammospheres) are a better representation of solid tumours than those cultured in monolayer [52]. We determined the ability of **1** to inhibit the formation and reduce the viability of mammospheres. At a non‐lethal dose (IC_20_ value for 5 days), **1** was able to significantly inhibit mammosphere formation with respect to the number and size (volume) of mammospheres formed (Figures 2A,B, and S29). The inhibitory effect of **1** was comparable to salinomycin and significantly more than **2**, **3**, and cisplatin under identical conditions (Figures 2A,B, and S29). The inhibitory effect of **1** was also markedly more than diflunisal, 1‐ethynyl pyrene, a 1:1 cocktail of **2** and diflunisal, or a 1:1:1 cocktail of **3**, 1‐ethynyl pyrene, and diflunisal (Figures 2A,B, and S29). With respect to mammosphere viability, the IC_50_ value for **1** towards mammospheres was 3‐ to 21‐fold lower than **2**, **3**, salinomycin and cisplatin, demonstrating its superiority in killing breast CSCs cultured in three‐dimensional systems (Figure S29 and Table 1) [51, 53]. Cobalt(III) complex **1** was also significantly more (*p* < 0.05, *n* = 18) potent towards mammospheres than diflunisal, 1‐ethynyl pyrene, a 1:1 cocktail of **2** and diflunisal, or a 1:1:1 cocktail of **3**, 1‐ethynyl pyrene, and diflunisal, indicating that the mammosphere activity of the preformed complex **1** is better than its individual parts administered together (Figure S30 and Table 1). It should be noted that the mammosphere assay does not assess the intra‐spheroidal distribution, penetration depth or ligand‐release characteristics of **1** within spheroids. Furthermore, this assay does not directly model a clinical treatment scenario, rather it serves as a preclinical measure of breast CSC activity [52, 54].

**TABLE 1:** IC_50_ values of cobalt(III) complexes **1**‐**3**, diflunisal, 1‐ethynyl pyrene, **2** + diflunisal (1:1), **3** + diflunisal + 1‐ethynyl pyrene (1:1:1), cisplatin, carboplatin, and salinomycin against HMLER and HMLER‐shEcad cells, and HMLER‐shEcad mammospheres.

Compound	HMLER IC_50_ [µM]^a^	HMLER‐shEcad IC_50_ [µM]^a^	Mammospheres IC_50_ [µM]^b^
**1**	1.02 ± 0.01	0.98 ± 0.02	4.61 ± 0.01
**2**	6.04 ± 0.33	15.39 ± 0.03	96.60 ± 4.81
**3** ^c^	>100	>100	>133
Diflunisal	33.94 ± 1.39	68.30 ± 0.03	>133
1‐Ethynyl pyrene	12.03 ± 0.27	7.82 ± 0.19	>133
**2** + Diflunisal	n.d.	12.70 ± 0.81	68.90 ± 10.04
**3** + Diflunisal + 1‐ethynyl pyrene	n.d.	12.28 ± 1.13	70.86 ± 15.31
Cisplatin^c^	2.57 ± 0.02	5.65 ± 0.30	13.50 ± 2.34
Carboplatin^c^	67.31 ± 2.80	72.39 ± 7.99	18.06 ± 0.40
Salinomycin^c^	11.43 ± 0.42	4.23 ± 0.35	18.50 ± 1.50

^a^ Determined after 72 h incubation (mean of three independent experiments ± SD).

^b^ Determined after 5 days incubation (mean of three independent experiments ± SD).

^c^ Reported in references 50, 51 and 53. n.d. not determined.

**FIGURE 2:**
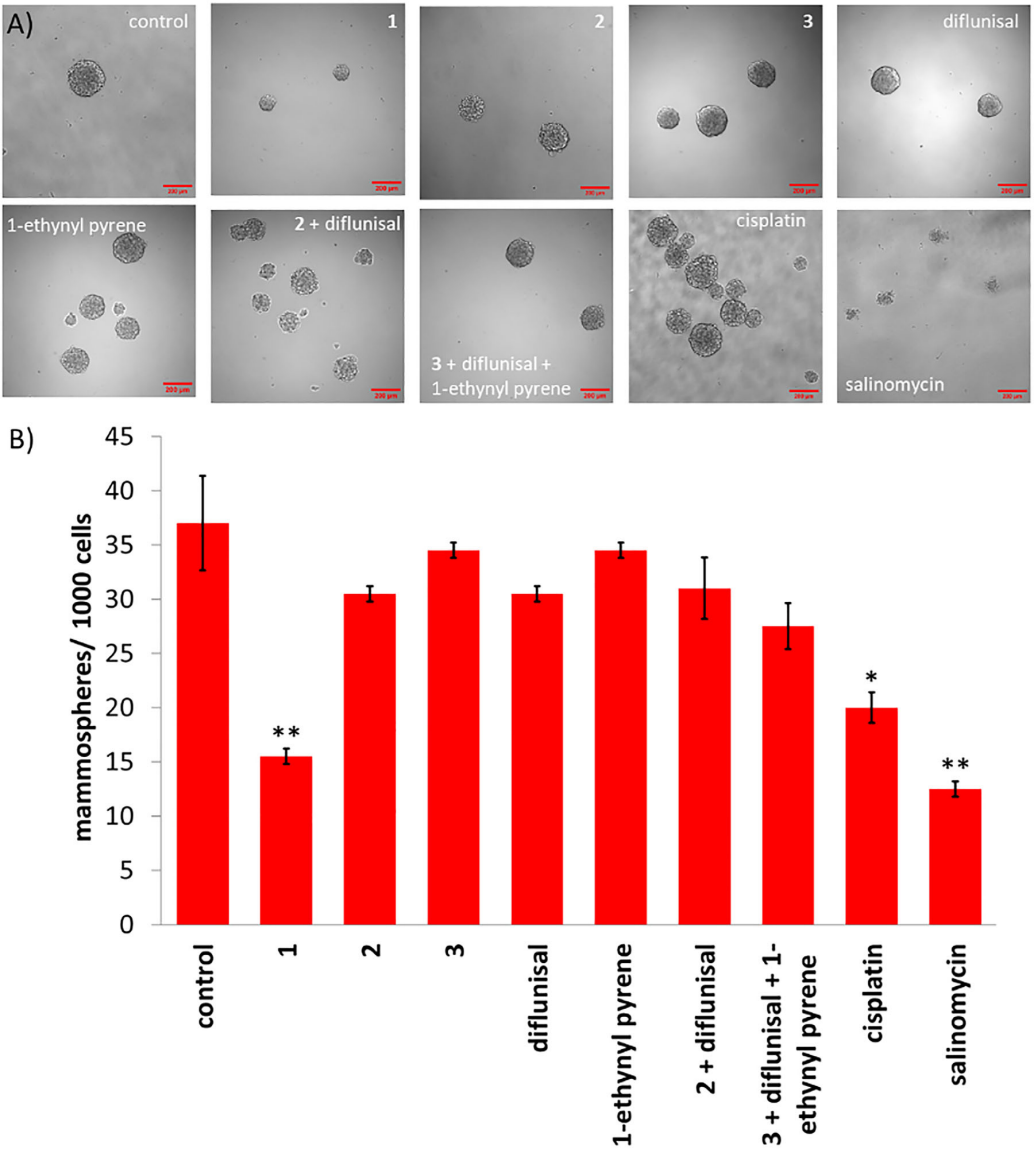
(A) Representative bright‐field images (x 10) of mammospheres in the absence and presence of cobalt(III) complexes **1**‐**3**, diflunisal, 1‐ethynyl pyrene, **2** + diflunisal (1:1), **3** + diflunisal + 1‐ethynyl pyrene (1:1:1), cisplatin or salinomycin (IC_20_ value for 5 days). Scale bar = 200 µm. (B) Quantification of mammosphere formation with HMLER‐shEcad cells untreated and treated with cobalt(III) complexes **1**‐**3**, diflunisal, 1‐ethynyl pyrene, **2** + diflunisal (1:1), **3** + diflunisal + 1‐ethynyl pyrene (1:1:1), cisplatin or salinomycin (IC_20_ value for 5 days). Error bars represent standard deviations and Student *t*‐test, *p < 0.05 and **p < 0.01.

### 2.4 Methuosis Hallmarks in Breast Cancer Stem Cells

Further cell‐based studies were conducted to shed light on the mechanism of action of **1**. ICP‐MS was used to determine cobalt levels in HMLER‐shEcad cells treated with **1** (1 µM for 24 h). It should be noted that ligand exchange, metabolism, or partial decomposition of **1** may occur following cellular uptake and that ICP‐MS does not differentiate between the parent complex and cobalt‐containing metabolites (it simply quantifies cobalt levels). The ICP‐MS data suggested that **1** (as the parent complex or cobalt‐containing metabolites) was readily taken up by HMLER‐shEcad cells (55.27 ± 0.03 ng of Co/ million cells), with the majority of the cellular cobalt signal detected in the cytoplasm (72%) as opposed to the nucleus (6%) or membrane (22%) (Figure 3A). This suggests that **1** (as the parent complex or cobalt‐containing metabolites) is likely to have cytoplasmic targets. Upon treatment of HMLER‐shEcad cells with **1** (2 × IC_50_ value for 48 h) large cytoplasmic vacuoles were observed (Figure 3B). Cytoplasmic vacuolation is synonymous with certain modes of cell death such as paraptosis, autophagy and methuosis [9, 12, 13, 14, 55]. The cytoplasmic vacuolation induced by **1** was not attenuated in the presence of cycloheximide (0.1 µM), chloroquine (10 µM) or 3‐methyladenine (1 mM) (Figures 3B and S31). Given that cycloheximide is a protein synthesis inhibitor that inhibits paraptosis [56], and chloroquine and 3‐methyladenine both halt autophagic flux and block autophagosome fusion [57], the morphological features observed for **1**‐treated HMLER‐shEcad cells are unlikely to result from paraptosis or autophagy. Flow cytometry studies were performed with the CYTO‐ID green dye to further probe the ability of **1** to induce autophagy. CYTO‐ID green fluoresces inside pre‐autophagosomes, autophagosomes and autophagolysosomes, and so can reliably detect autophagy [58]. HMLER‐shEcad cells dosed with **1** (IC_50_ value or 2 × IC_50_ value for 24 h) did not display increased pre‐autophagosomes, autophagosomes or autophagolysosomes levels, indicative of a non‐autophagic cell death mechanism (Figure 3C). As expected, treatment with rapamycin (1 µM for 24 h), a well‐known autophagy‐inducer, did appreciably increase pre‐autophagosomes, autophagosomes or autophagolysosomes levels (Figure S32). Complementary cytotoxicity studies showed the IC_50_ value of **1** towards HMLER‐shEcad cells did not significantly increase (*p* < 0.05, *n* = 18) in the presence of cycloheximide (1 µM), chloroquine (10 µM) or 3‐methyladenine (1 mM) (Figure S33 and Table S4), further supporting the notion that **1** does not induce paraptosis or autophagy of breast CSCs.

**FIGURE 3:**
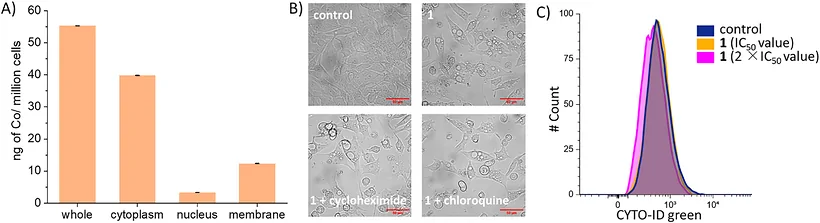
(A) Cobalt content (ng of Co/10^6^ cells) in various cellular components upon treatment of HMLER‐shEcad cells with **1** (1 µM for 24 h). (B) Representative bright‐field images (× 60) of untreated HMLER‐shEcad cells, HMLER‐shEcad cells treated with **1** (2 × IC_50_ value for 48 h), HMLER‐shEcad cells co‐treated with **1** (2 × IC_50_ value for 48 h) and cycloheximide (0.1 µM for 48 h), and HMLER‐shEcad cells co‐treated with **1** (2 × IC_50_ value for 48 h) and chloroquine (10 µM for 48 h). (C) Representative histograms displaying the green fluorescence emitted by CYTO‐ID green reagent‐stained HMLER‐shEcad cells untreated (blue) and treated with **1** (IC_50_ value, orange) or **1** (2 × IC_50_ value, pink) for 24 h.

Given that the cytoplasmic vacuolation observed in **1**‐treated HMLER‐shEcad cells was not the result of paraptosis or autophagy, the possibility that it was a manifestation of methuosis was investigated. Bright‐field imaging studies showed that the cytoplasmic vacuolation induced in HMLER‐shEcad cells following treatment with **1** (2 × IC_50_ value for 48 h) was markedly reduced when cells were pre‐treated with macropinocytosis inhibitor 5‐(*N*‐ethyl‐*N*‐isopropyl)amiloride (EIPA, 4 µM for 6 h) (Figure S34). This suggests that the cytoplasmic vacuolation induced by **1** is associated with aberrant macropinocytosis and methuosis. Live‐cell imaging studies were conducted to monitor cytoplasmic vacuole formation. Following treatment of HMLER‐shEcad cells with **1** (4 × IC_50_ value), cytoplasmic vacuolation was observed within 20 min, with nascent macropinosomes fusing to form larger vacuoles (Figure S35). These observations are consistent with previous reports describing vacuole formation induced by chalcone‐related and azaindole‐based methuosis inducers [59, 60]. Furthermore, complementary cytotoxicity studies revealed that the IC_50_ value of **1** towards HMLER‐shEcad cells increased significantly (*p* < 0.05, *n* = 18) in the presence of EIPA (4 µM pre‐treatment for 6 h), providing further support for the involvement of methuosis in the mode of action of **1** (Figure S36 and Table S4). Vacuoles formed during methuosis originate from macropinosomes and possess late endosomes features, and are Rab7‐ and LAMP1‐positive [13]. Immunoblotting analysis of HMLER‐shEcad cells dosed with **1** (0.5–2 µM for 72 h) indicated a noticeable increase in Rab7‐ and LAMP1‐expression (Figures 4A and S37), suggestive of methuosis induction. Mitogen‐activated protein kinases (MAPK) pathways have been implicated in the initiation of methuosis [61, 62]. Immunoblotting studies revealed that HMLER‐shEcad cells treated with **1** (1–4 µM for 24 h) exhibited enhanced phosphorylation of Jun‐amino‐terminal kinase (JNK) and p38 MAPK and their corresponding downstream effectors, c‐Jun and MAP kinase‐activated protein kinase 2 (MAPKAPK‐2), respectively (Figure S38). This suggests that **1**‐induced methuosis in HMLER‐shEcad cells could be associated to JNK and p38 MAPK pathway activation. Methuosis‐inducers such as bis(acetylacetonato)oxidovanadium(IV) and the *N*‐phenyl‐4‐pyrimidinediamine derivative DZ‐514 have been reported to induce cell cycle arrest at the G2/M phase [20, 61]. To understand the impact of **1** on the cell cycle profile of breast CSCs, DNA‐flow cytometric studies were performed. The cell cycle profile of HMLER‐shEcad cells treated with **1** (2 × IC_50_ value for 24–72 h) displayed a significant decrease in the proportion of cells in the G1‐phase and a concurrent increase in the proportion of cells in the G2/M‐phase (Figure 4B), implicative of G2/M phase arrest. Other characteristics for methuosis‐inducing agents include an increase in intracellular reactive oxygen species (ROS) levels and mitochondrial membrane potential (MMP) disruption [13, 61]. Changes in intracellular ROS levels upon incubation of HMLER‐shEcad cells with **1** (2 × IC_50_ value or 4 × IC_50_ value) were measured using the ROS detector, 6‐carboxy‐2,7‐dichlorodihydrofluorescein diacetate (DCFH‐DA) [63], over the course of 24 h (Figure 4C). HMLER‐shEcad cells dosed with **1** exhibited a significant (*p* < 0.05) increase in intracellular ROS levels, at short (0.5 h) and prolonged (24 h) incubation times (up to 44% increase). This suggests that **1** has the potential to induce ROS‐related methuosis. The effect of **1** on the MMP was assessed by flow cytometry, using the JC‐1 dye. If the MMP is disrupted, JC‐1 forms green‐emitting monomers [64]. HMLER‐shEcad cells incubated with **1** (IC_50_ value for 24 h) and the JC‐1 dye displayed increased green fluorescence compared to untreated cells, indicative of a compromised MMP (Figure S39A). A similar result was observed for HMLER‐shEcad cells treated with carbonyl cyanide *m*‐chlorophenyl hydrazone (CCCP) (1 µM and 5 µM for 24 h), a known mitochondrial membrane depolariser (Figure S39B). Overall, the mechanistic investigations suggest that **1** can induce hallmarks associated to methuosis in breast CSCs.

**FIGURE 4:**
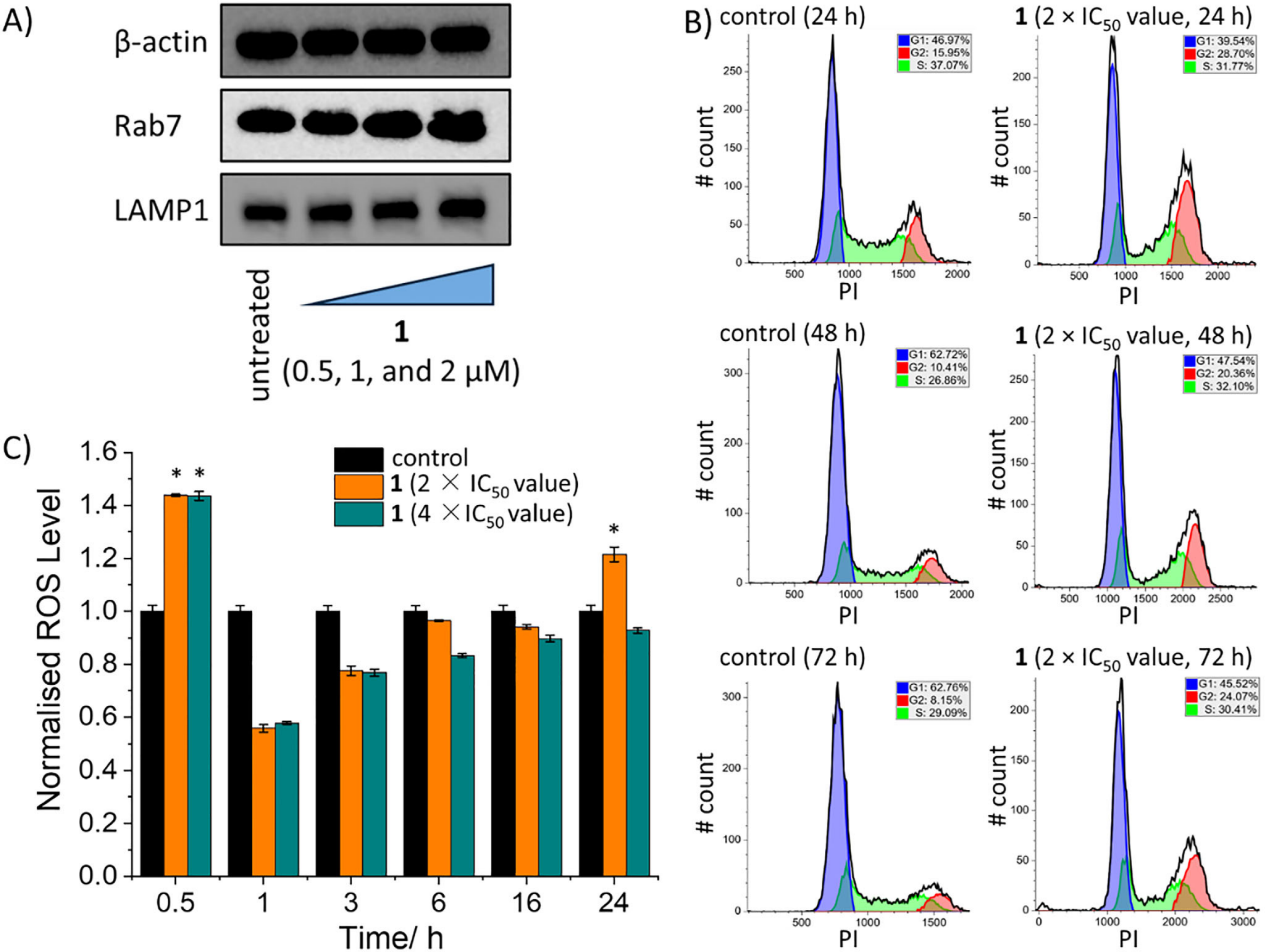
(A) Immunoblotting analysis of proteins related to the methuosis pathway. Protein expression in HMLER‐shEcad cells following treatment with **1** (0.5–2 µM after 72 h incubation). (B) Histograms representing the different phases of the cell cycle (%) for HMLER‐shEcad cells in the absence and in the presence of **1** (2 × IC_50_ value) for 24–72 h. (C) Normalised ROS activity in untreated HMLER‐shEcad cells and HMLER‐shEcad cells treated with **1** (2 × IC_50_ value or 4 × IC_50_ value for 0.5, 1, 3, 6, 16 and 24 h). Error bars represent standard deviations and Student *t*‐test, *p < 0.05.

### 2.5 Perturbation of Cyclooxygenase‐2 Expression and Induction of Immunogenic Cell Death

As **1** contains a diflunisal unit that is capable of modulating COX‐2 levels [41, 42], flow cytometry and immunoblotting studies were performed to assess the effect of **1** on COX‐2 expression and, consequently, the iDAMP axis. HMLER‐shEcad cells were first exposed to lipopolysaccharide (LPS) (2.5 µg/mL for 24 h) to elevate basal COX‐2 levels, followed by treatment with **1** or diflunisal, after which COX‐2 expression was determined. Flow cytometric analysis revealed a marked increase in HMLER‐shEcad cells exhibiting low COX‐2 expression following treatment with **1** (4 × IC_50_ value for 48 h) or diflunisal (20‐40 µM for 48 h), consistent with COX‐2 downregulation (Figure S40). Immunoblotting studies indicated that the expression of COX‐2 fragments decreased upon treatment with **1** (0.5–2 µM for 72 h) (Figures S41 and S42). To determine whether **1** induces COX‐2‐dependent breast CSC death, cytotoxicity studies were conducted using HMLER‐shEcad cells exposed to LPS (2.5 µg/mL for 24 h) prior to treatment. Under these conditions, the potency of **1** against HMLER‐shEcad cells decreased significantly (IC_50_ value = 1.80 ± 0.07 µM, *p* < 0.05, *n* = 18) (Figure S43) supporting a COX‐2‐dependent mechanism of **1**‐induced breast CSC death.

Inhibition or downregulation of COX‐2 has been reported to promote ICD in both bulk cancer cells and CSCs [38]. Given the capacity of **1** to downregulate COX‐2 and potentially modulate the iDAMP axis in breast CSCs, we investigated whether **1** could promote DAMP release from these cells. The effect of **1** on the release or exposure of four key DAMPs associated with ICD, adenosine triphosphate (ATP), high mobility group box 1 (HMGB‐1), calreticulin (CRT) and phosphatidylserine (PS) was examined in breast CSCs [37]. Treatment of HMLER‐shEcad cells with **1** (2–4 µM for 24 h) resulted in a dose‐dependent increase in ATP release, reaching up to 20.4‐fold above untreated controls, as determined using an established luciferase‐based assay (Figure 5A). Exposure to cisplatin (50 µM for 24 h) likewise resulted in a significant increase in ATP release, reaching 1.4‐fold that of untreated cells (Figure 5A). Immunoblotting studies further revealed substantially reduced HMGB‐1 levels in HMLER‐shEcad cells following treatment with **1** (1–4 µM for 24 h), consistent with the extracellular release of HMGB‐1 (Figure 5B). Flow cytometric analysis revealed that HMLER‐shEcad cells treated with **1** (2 × IC_50_ value or 4 × IC_50_ value for 24 h) showed no appreciable increase in membrane‐exposed CRT levels (Figures S44A). In contrast, shorter exposure to **1** (2 × IC_50_ value or 4 × IC_50_ value for 6 h) resulted in a noticeable increase in cell surface CRT relative to untreated cells (Figures S44B). Consistent with previous observations, exposure of HMLER‐shEcad cells to a cocktail of cisplatin (150 µM for 24 h) and thapsigargin (7 µM for 24 h) promoted CRT accumulation at the cell surface (Figure S44C). PS externalisation may also contribute to an immune response by promoting immune cell recognition, recruitment and subsequent clearance of dying cells [65]. Treatment of HMLER‐shEcad cells with **1** (4 × IC_50_ value for 72 h) resulted in a pronounced increase in cell surface PS levels (Figure 5C), suggesting that **1**‐induced breast CSC death may promote immunogenicity through PS exposure. HMLER‐shEcad cells exposed to cisplatin (25 µM for 72 h) similarly exhibited increased cell surface PS levels (Figure S45). Collectively, these DAMP profiling studies indicate that **1** is capable of inducing ICD in breast CSCs.

**FIGURE 5:**
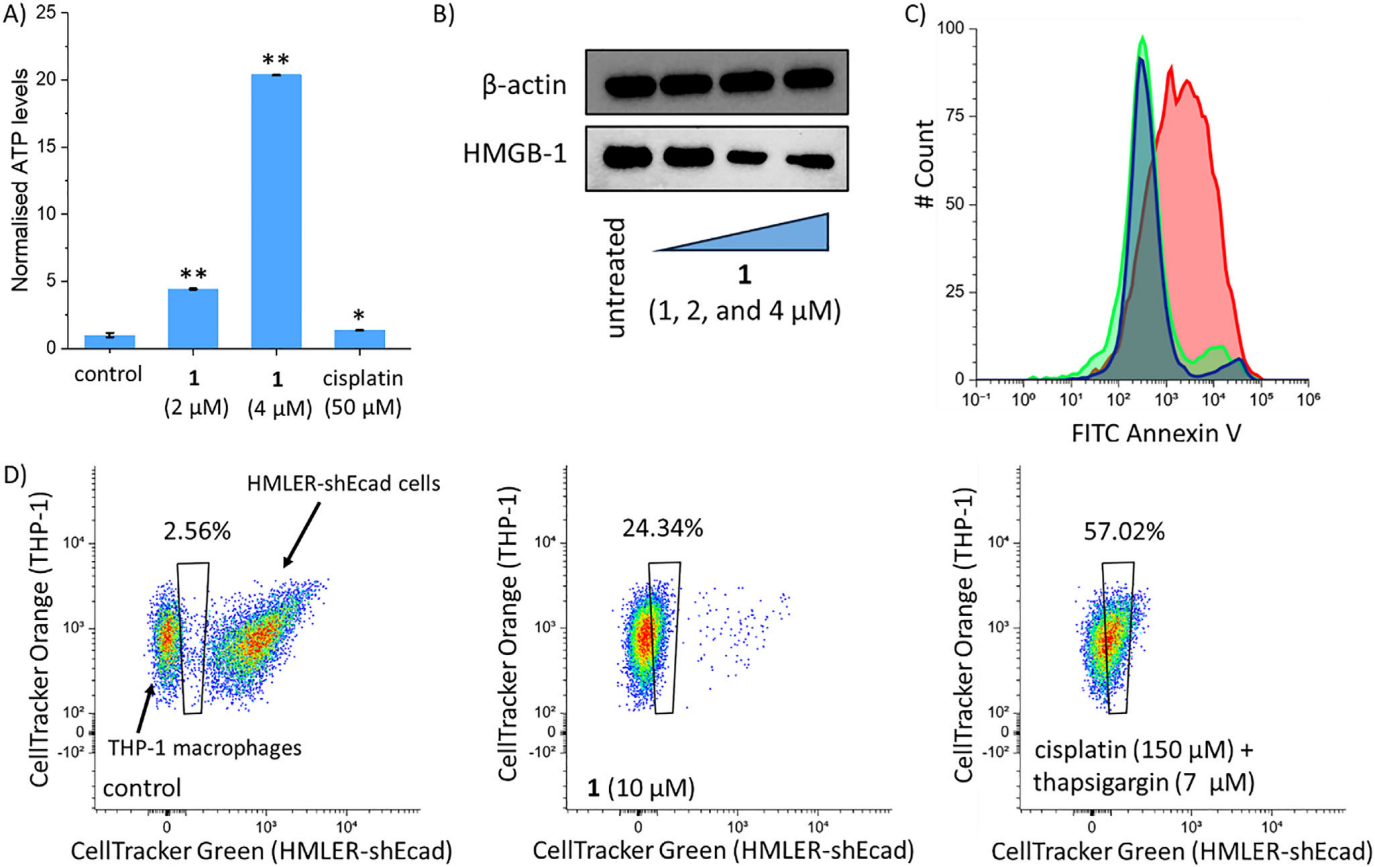
(A) Normalised extracellular ATP released from HMLER‐shEcad cells untreated and treated with **1** (2 or 4 µM) or cisplatin (50 µM) for 24 h. Error bars represent standard deviations and Student *t*‐test, *p < 0.05 and **p < 0.01. (B) Immunoblotting analysis of high mobility group box 1 (HMGB‐1). Protein expression in HMLER‐shEcad cells following treatment with **1** (1–4 µM for 24 h). (C) Representative histograms displaying the green fluorescence emitted by FITC Annexin V‐stained HMLER‐shEcad cells untreated (blue), and treated with **1** (2 × IC_50_ value) (green) or **1** (4 × IC_50_ value) (red) for 72 h. (D) Representative two‐dimensional scatter plots of CellTracker green‐stained HMLER‐shEcad cells untreated and treated with **1** (10 µM) or cisplatin (150 µM) and thapsigargin (7 µM) for 24 h and then co‐cultured with CellTracker Orange‐stained THP‐1 macrophages for 2 h.

Given that **1** induced ICD and promoted DAMP release in breast CSCs, we next assessed whether **1**‐treated HMLER‐shEcad cells could undergo macrophage‐mediated phagocytosis using an in vitro phagocytosis assay. HMLER‐shEcad cells were first labelled with CellTracker Green (Figure S46), exposed to **1** (5–10 µM for 24 h), and subsequently co‐incubated with CellTracker Orange‐labelled macrophages (Figure S46) for 2 h. The two‐dimensional scatter plots in Figures 5D and S47 show the population of macrophages containing engulfed, double‐stained HMLER‐shEcad cells. Exposure of HMLER‐shEcad cells to the cobalt(III) complex **1** (5–10 µM for 24 h) markedly increased their uptake by macrophages, with engulfment reaching up to 9.5‐fold above that observed in untreated cells (Figure 5D). In contrast, treatment of HMLER‐shEcad cells with either diflunisal (20 µM for 24 h) or **2** (20 µM for 24 h) did not significantly enhance macrophage‐mediated phagocytosis (Figure S47). These findings indicate that macrophage‐mediated phagocytosis of breast CSCs is induced specifically by the preformed cobalt(III) complex **1**, rather than by its individual components. Treatment of HMLER‐shEcad cells with a cocktail of cisplatin (150 µM for 24 h) and thapsigargin (7 µM for 24 h) produced a significant increase in the double‐stained population (22.3‐fold above untreated cells), indicative of enhanced phagocytosis (Figure 5D). Collectively, the phagocytosis assay demonstrates that **1** induces breast CSC death in a manner that facilitates their subsequent engulfment by macrophages.

### 2.6 Immunotherapeutic Efficacy in a Murine Breast Cancer Model

In vivo studies were performed to evaluate the functional outcomes of **1** with respect to anti‐breast cancer cell immunogenicity. To enable efficient and intact delivery of **1** to the tumour microenvironment, it was encapsulated within polymeric nanoparticles. Complex **2** was similarly encapsulated and used as a comparator. Specifically, **1** and **2** were encapsulated into the amphiphilic polymer distearoyl phosphatidylethanolamine‐polyethylene glycol 2000 (DSPE‐PEG_2000_) via the nanoprecipitation method to yield the corresponding nanoparticle formulations, **NP‐1** and **NP‐2**, respectively (Figure 6A). The concentrations of **1** and **2** in **NP‐1** and **NP‐2**, respectively, were quantified by atomic absorption spectrometry (AAS). The average hydrodynamic diameters of **NP‐1** and **NP‐2**, as determined by dynamic light scattering (DLS), were 89 nm (polydispersity index = 0.07) and 117 nm (polydispersity index = 0.12), respectively (Figure 6B). The morphologies of **NP‐1** and **NP‐2** were examined by transmission electron microscopy (TEM). As shown in Figure 6C, both **NP‐1** and **NP‐2** exhibited spherical morphologies with uniform size distributions.

**FIGURE 6:**
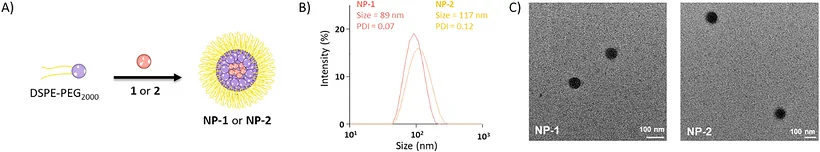
(A) Schematic showing the encapsulation of **1** and **2** into DSPE‐PEG_2000_ nanoparticles (**NP‐1** and **NP‐2**). (B) The average nanoparticle size and PDI of **NP‐1** and **NP‐2** determined by DLS. (C) Representative TEM images of **NP‐1** and **NP‐2**. Scale bar = 100 nm.

To investigate the biodistribution of **1** in BALB/c mice bearing 4T1 breast tumours, fluorescence imaging studies were conducted. Complex **1** was co‐encapsulated with the near‐infrared fluorescent probe cyanine 7.5 (Cy7.5) into DSPE‐PEG_2000_ nanoparticles, yielding the formulation **NP‐1@Cy7.5**. **NP‐1@Cy7.5** was administered intravenously to tumour‐bearing BALB/c mice at a dose of 0.2 mg Co kg^−1^, and whole‐body fluorescence imaging was performed using a CT fluorescence imaging system at various time points over a 3‐day period (Figure 7A–C). At the conclusion of the imaging study, selected organs were harvested and subjected to ex vivo fluorescence imaging to assess biodistribution. Time‐course fluorescence imaging revealed a progressive increase in intratumoral accumulation of **NP‐1@Cy7.5**, reaching a maximum at 24 h post‐administration (Figure 7A,B). Beyond 24 h, a marked decrease in intratumoral fluorescence intensity was observed (Figure 7A,B), which is likely attributable to systemic clearance, primarily via hepatic and renal pathways. Consistent with this observation, ex vivo biodistribution analysis at 3 days post‐administration showed the highest accumulation of **1** in the liver, followed by the tumour tissue (Figures 7C and S48). Importantly, these results demonstrate that encapsulation of **1** within DSPE‐PEG_2000_ nanoparticles enables effective delivery to tumour sites, potentially mediated by the enhanced permeability and retention (EPR) effect.

**FIGURE 7:**
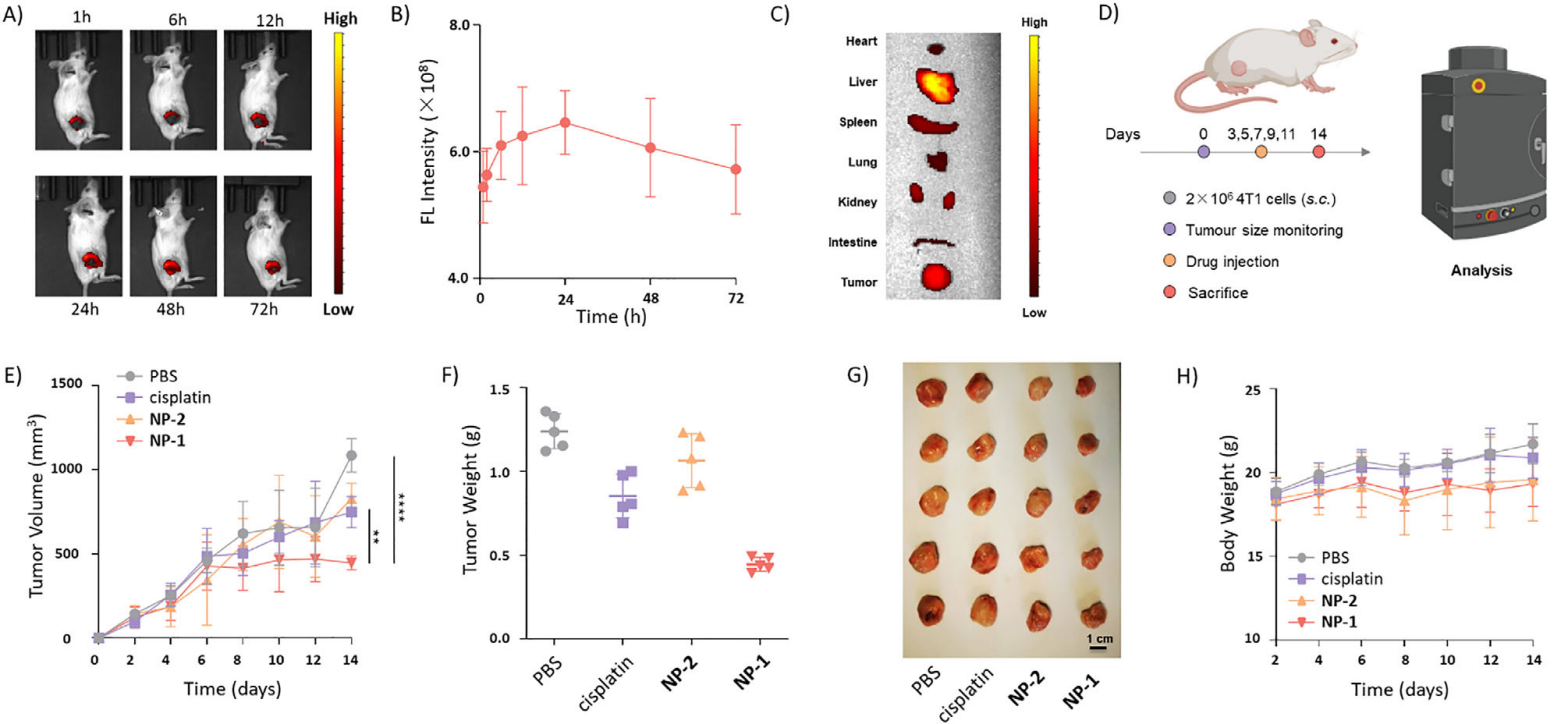
(A) Representative in vivo CT fluorescence imaging of 4T1 tumour‐bearing mice over a 3‐day period following intravenous administration of **NP‐1@Cy7.5** (0.2 mg Co kg^−1^). (B) Intratumoral fluorescence intensity of 4T1 tumour‐bearing mice over a 3‐day period following intravenous administration of **NP‐1@Cy7.5** (0.2 mg Co kg^−1^). (C) Ex vivo fluorescence imaging of various tissues excised from 4T1 tumour‐bearing mice after 3 days following intravenous administration of **NP‐1@Cy7.5** (0.2 mg Co kg^−1^). (D) Schematic of the experimental design for determining the tumour growth inhibition profiles. (E) Averaged tumour growth inhibition curves of 4T1 tumour‐bearing mice (*n* = 5 mice), following intravenous administration of the vehicle (PBS) or cisplatin (1.5 mg Pt kg^−1^) or **NP‐2** (1.5 mg Co kg^−1^) or **NP‐1** (1.5 mg Co kg^−1^). The data is represented as the mean ± SD (*n* = 5 mice, **p < 0.01 and ****p < 0.0001, one‐way analysis of variance (ANOVA) with Tukey's multiple comparisons test). (F) Excised tumour weight after 14 days upon different treatments. (G) Images of excised tumours from 4T1 tumour‐bearing mice after 14 days upon different treatments. (H) Body weight of 4T1 tumour‐bearing mice during the efficacy study lasting 14 days.

Survival analysis and maximum tolerated dose studies (*n* = 6 mice) were conducted with three doses of **NP‐1** to determine the administration dose for subsequent in vivo studies (Figures S49 and S50). The study showed that the body weight of BALB/c mice was relatively stable when dosed with 0.5 or 1.5 mg Co kg^−1^ of **NP‐1** every 2 days over a 12‐day period (Figure S49). When the dose was increased to 3 mg Co kg^−1^ of **NP‐1**, body weight loss was observed (Figure S49). At the end of the treatment period, histological changes in heart, liver, and lung tissue were assessed by hematoxylin and eosin (H&E) staining. While mice treated with 0.5 or 1.5 mg Co kg^−1^ of **NP‐1** showed no observable adverse effects, those receiving 3 mg Co kg^−1^ of **NP‐1** exhibited marked off‐target toxicity (Figure S50). In this treatment group, heart tissue displayed pale, loosened cytoplasm in cardiomyocytes accompanied by widened intercellular spaces, but without significant inflammatory infiltration or myocardial necrosis, suggesting mild cardiac edema. Liver tissue exhibited diffuse macrovesicular steatosis, characterised by hepatocytes containing numerous round, clear vacuoles of varying sizes, consistent with disrupted hepatic lipid homeostasis and abnormal intracellular lipid accumulation. Lung tissue showed abundant neutrophils with lobulated nuclei and intensely blue‐stained chromatin, indicative of an acute inflammatory response. Based on these findings, 1.5 mg Co kg^−1^ of **NP‐1** was selected as the administration dose.

Next the anti‐tumour efficacy of **NP‐1** was evaluated in BALB/c mice bearing 4T1 breast tumours. BALB/c mice were inoculated with 4T1 cells, and after tumour establishment, **NP‐1** was administered intravenously at a dose of 1.5 mg Co kg^−1^ every 2 days for a further 8 days (*n* = 5 mice). Tumour volumes and body weight were measured every 2 days over a 14‐day period (Figure 7D). Control groups were treated with the vehicle (PBS), **1** (1.5 mg Co kg^−1^), **NP‐2** (1.5 mg Co kg^−1^), or cisplatin (a clinically established metallodrug, 1.5 mg Pt kg^−1^) [66] to enable comparisons. We note, however, that equivalent metal dosing does not necessarily constitute an equivalent basis for comparison, given the distinct physicochemical and pharmacological properties of different metal complexes and nanoparticle formulations. As shown in Figure 7E–G, tumour growth (volume and weight) was significantly inhibited in the **NP‐1**‐treated group compared with the vehicle control (*p* < 0.0001 at day 14). The anti‐tumour efficacy of **1** was comparable to that observed for **NP‐1** (Figures 7E–G and S51A–C). This indicates that the in vivo anti‐tumour activity of **NP‐1** is primarily attributable to **1** (the active payload in **NP‐1**), rather than the DSPE‐PEG_2000_ nanoparticle carrier. Also, **NP‐1** exhibited a significantly greater tumour growth inhibitory effect than both the **NP‐2**‐ and cisplatin‐treated groups (Figure 7E–G). These findings suggest that the presence of both the cobalt(III)‐cyclam‐pyrene and diflunisal moieties within **1** is critical for its in vivo anti‐tumour efficacy. Across all treatment groups, mice maintained stable body weights throughout the study, indicating favorable safety profiles at the administered doses (Figures 7H and S51D).

Next, the in vivo immunotherapeutic potential of **NP‐1** was evaluated in immunocompetent BALB/c mice bearing 4T1 breast tumours. Effective immunotherapy typically induces immune cell infiltration (mature dendritic cells and T‐cells) into tumour‐draining lymph nodes and tumour tissue, along with remodeling of the immunosuppressive tumour microenvironment [67]. 4T1 tumour‐bearing mice were subject to intravenous administration of **NP‐1** (1.5 mg Co kg^−1^) or **NP‐2** (1.5 mg Co kg^−1^) or cisplatin (1.5 mg Pt kg^−1^) or the vehicle (PBS) every 2 days for 8 days (*n* = 5 mice) and after 14 days the tumour‐draining lymph nodes and tumour tissues were extracted and analysed by flow cytometry using relevant immune cell markers (Figure 8A). Analysis of tumour‐draining lymph nodes revealed that mice treated with **NP‐1** exhibited significantly higher levels of CD80^+^ CD86^+^ mature dendritic cells (60.7%) than to those treated with **NP‐2** (34.3%), cisplatin (43.4%) or the vehicle (27.0%) (Figure 8B). Mature dendritic cells can activate T lymphocytes through antigen presentation, and thereby initiate an adaptive immune response [68]. Therefore, the proportion of tumour infiltrating CD8^+^ T‐cells was determined. The percentage of CD8^+^ T‐cells in tumours of mice treated with **NP‐1** (36.7 %) was significantly higher than in tumours of mice treated with the vehicle (19.0%) and markedly more than those treated with **NP‐2** (24.1%) or cisplatin (27.7%) (Figure 8C). Tumour‐associated macrophages are a major immune cell population in tumours and exist as two phenotypes: M1 and M2 [69, 70]. M1 tumour‐associated macrophages (F4/80^+^ CD80^+^ CD206^−^) are immunostimulatory and promote T‐cell recruitment, whereas M2 tumour‐associated macrophages (F4/80^+^ CD80^−^ CD206^+^) are immunosuppressive and support tumour growth, invasion and metastasis [69, 70]. Therefore, the ability of **NP‐1** to reprogram M2 tumour‐associated macrophages into M1 tumour‐associated macrophages was investigated. Flow cytometry studies of homogenised tumour cells indicated that **NP‐1** treatment resulted in a M1/M2 phenotypic value of 1.80 (value for vehicle treatment = 0.17), distinctly higher than that observed upon treatment with **NP‐2** (0.44) or cisplatin (0.82) (Figure 8D). Another factor influencing intratumoral T‐cell recruitment is the proportion of myeloid‐derived suppressor cells (Gr‐1^+^ CD11b^+^ CD45^+^), which inhibit CD8^+^ T‐cell function and proliferation [71, 72]. As depicted in Figure 8E, **NP‐1** treatment resulted in a 27% decrease in intratumoral myeloid‐derived suppressor cells (57.2%) compared to the vehicle (78.8%), and levels were also markedly lower than treatment with **NP‐2** (67.5%) or cisplatin (61.4%). Collectively, the data on M1/M2 polarisation and the proportion of intratumoral myeloid‐derived suppressor cells suggest that **NP‐1**‐induced intratumoral T‐cell recruitment may be linked to effective reprogramming of the tumour microenvironment via M2‐to‐M1 polarisation and depletion of myeloid‐derived suppressor cells. Finally, we conducted tumour rechallenge studies to evaluate the ability of **NP‐1** to elicit a durable anti‐tumour response. Briefly, 4T1 tumour‐bearing BALB/c mice were intravenously administered **NP‐1** (1.5 mg Co kg^−^^1^) or the vehicle (PBS) every 2 days for 8 days (*n* = 6 mice). Following completion of the treatment regimen, the primary tumour(s) were surgically resected, and the mice were rechallenged with a second subcutaneous inoculation of 4T1 cells (2 × 10^6^ cells). Tumour growth was subsequently monitored for 14 days. As shown in Figure S52, tumour growth, as measured by both tumour volume and tumour weight, was significantly suppressed in the **NP‐1**‐treated group compared with the vehicle control group (*p* < 0.0001 at day 14). This suggests **NP‐1** is potentially capable of inducing functional immune memory and providing protection against tumour re‐establishment upon rechallenge. Overall, the in vivo studies demonstrate that **NP‐1** can deliver **1** to tumour tissue, inhibit breast tumour growth, activate the immune system to attack breast tumours, and protect against tumour re‐establishment upon rechallenge.

**FIGURE 8:**
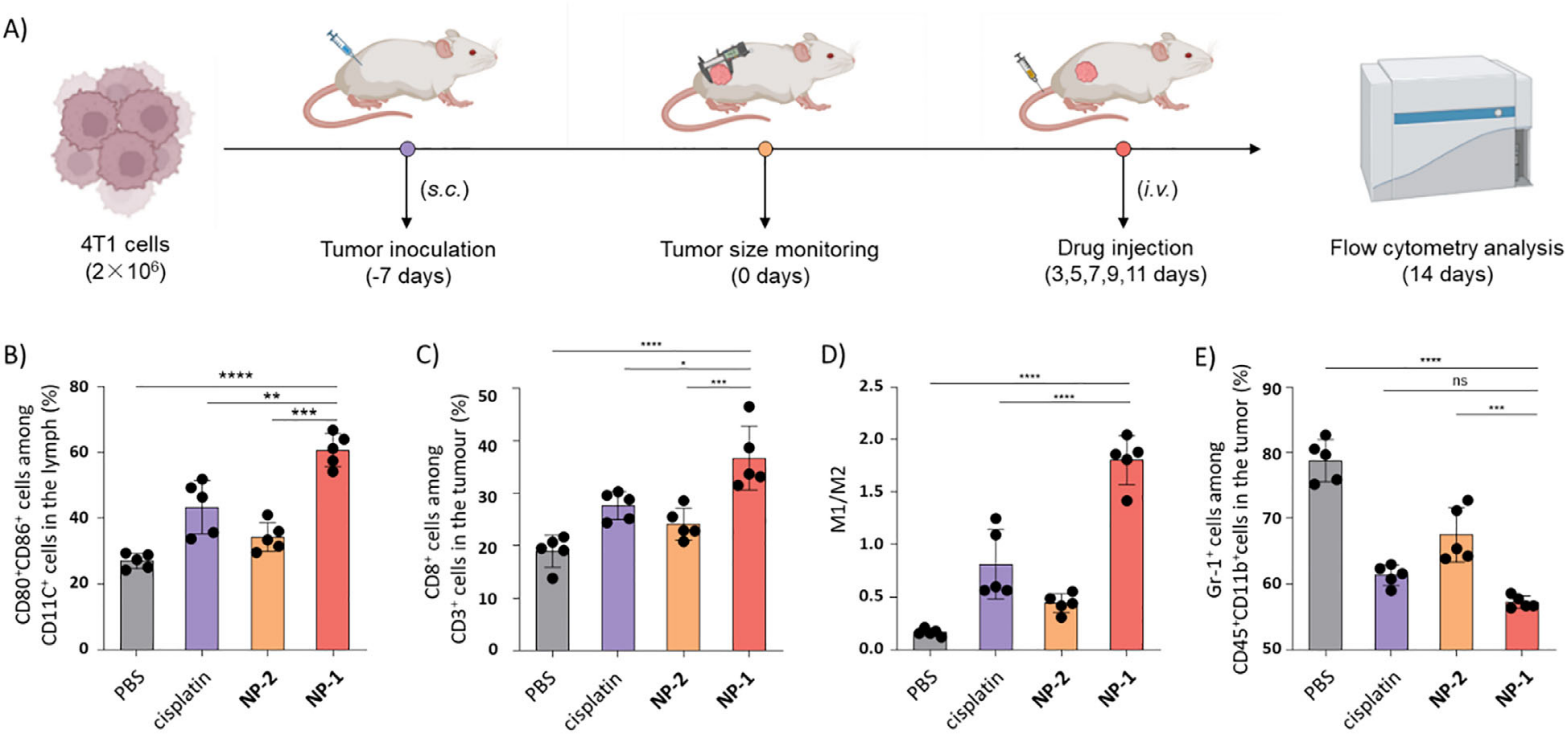
(A) Schematic of the experimental design used to determine in vivo immune system activation. (B) Flow cytometric study of CD80^+^ CD86^+^ mature dendritic cells gating on CD11c^+^ cells in tumour‐draining lymph nodes upon different treatments. The average percentage of CD80^+^ CD86^+^ mature dendritic cell populations (*n*=5) in each group is provided. (C) Flow cytometric study of CD8^+^ T‐cells gating on CD3^+^ cells in tumour tissue upon different treatments. The average percentage of CD8^+^ T‐cell populations (*n*=5) in each group is provided. (D) Average M1/M2 macrophage phenotypic values (*n*=5) in tumour tissue upon different treatments determined by flow cytometric analysis. (E) Flow cytometric study of Gr‐1^+^ myeloid‐derived suppressor cells gating on CD45^+^ and CD11b^+^ cells in tumour tissue upon different treatments. The average percentage of Gr‐1^+^ myeloid‐derived suppressor cells populations (*n*=5) in in each group is provided. The data is represented as the mean ± SD (*n* = 5 mice, *p < 0.05, **p < 0.01, ***p < 0.001 and ****p < 0.0001, one‐way and two‐way analysis of variance (ANOVA) with Bonferroni multiple comparisons post‐test).

## 3 Conclusion

In summary, we report the synthesis, full characterisation, and in vitro and in vivo anti‐breast CSC activity of a cobalt(III) complex (**1**) incorporating pyrene and diflunisal. X‐ray crystallography revealed that **1** adopts a distorted octahedral geometry. Time‐course UV–vis spectroscopy and mass spectrometry demonstrated that **1** is stable under cell culture conditions, while ICP‐MS confirmed its stability in human plasma, with minimal cobalt leaching. Additional mass spectrometry and emission studies indicated release of diflunisal in the presence of bio‐reductants. Cobalt(III)‐cyclam complex **1** displayed low micromolar potency towards breast CSCs grown in both two‐dimensional and three‐dimensional culture systems, and was significantly more active than cisplatin, carboplatin and salinomycin. Mechanistic investigations revealed that **1** induces hallmark features of methuosis in breast CSCs, including extensive cytoplasmic vacuolation, increased Rab7 and LAMP1 expression, activation of JNK and p38 MAPK signalling and downstream effectors, G2/M cell‐cycle arrest, elevated intracellular ROS levels, and MMP depolarisation. In addition, **1** downregulated COX‐2 expression in breast CSCs, and its cytotoxicity was attenuated by LPS, suggesting a mechanistic link between COX‐2 modulation and breast CSC susceptibility. DAMPs analysis in breast CSCs showed that treatment with **1** promotes extracellular ATP and HMGB‐1 release, together with CRT and PS exposure, consistent with the induction of ICD. Correspondingly, phagocytosis studies demonstrated efficient engulfment of breast CSCs treated with **1** by macrophages, highlighting its immunogenic potential. When administered as a polymeric nanoparticle formulation in a murine metastatic triple‐negative breast cancer model, **1** entered tumour tissue and inhibited tumour growth more effectively than cisplatin. The nanoparticle formulation also elicited a robust immunotherapeutic response, characterised by enhanced infiltration of T‐cells and mature dendritic cells into tumours and tumour‐draining lymph nodes. Further studies suggest that intratumoral T‐cell recruitment is associated with tumour microenvironment reprogramming through M2‐to‐M1 macrophage polarisation and depletion of myeloid‐derived suppressor cells. To the best of our knowledge, **1** is the first metal complex reported to induce characteristics consistent with methuosis in a CSC model. These findings provide a basis for further investigating the potential of metal‐based agents that induce characteristics consistent with methuosis as anti‐CSC agents.

## Author Contributions

**Jiaxin Fang**: conceptualization, methodology, data curation, investigation, validation, formal analysis, writing – original draft, writing – review and editing. **Sijie Wen**: conceptualization, methodology, data curation, investigation, validation, formal analysis, writing – original draft, writing – review and editing. **Xiao Feng**: methodology, data curation, investigation, validation, formal analysis, writing – review and editing. **Hao Ren**: writing – review and editing, methodology, validation, formal analysis, investigation, data curation. **Kuldip Singh**: methodology, validation, investigation, data curation, formal analysis, writing – review and editing. **Haihua Xiao**: conceptualization, methodology, data curation, investigation, validation, formal analysis, supervision, funding acquisition, project administration, writing – original draft, writing – review and editing. **Lingpu Zhang**: conceptualization, investigation, funding acquisition, writing – original draft, methodology, validation, writing – review and editing, formal analysis, project administration, supervision, data curation. **Kogularamanan Suntharalingam**: data curation, supervision, formal analysis, methodology, validation, conceptualization, investigation, funding acquisition, writing – original draft, writing – review and editing, project administration.

## Conflicts of Interest

The authors declare no conflicts of interest.

## Supporting information




**Supporting File**: anie74516‐sup‐0001‐SuppMat.pdf.

## Data Availability

The data that supports the findings of this study are available in the Supporting Information of this article.
