# Supplementary material for: Induction of Methuosis in Breast Cancer Stem Cells by a Cobalt(III) Complex Evokes Immunotherapeutic Response
Source: Angew Chem Int Ed Engl. 2026 Sep 10;65(44):e8926368. doi: 10.1002/anie.8926368 (PMC13703765; doi:10.1002/anie.8926368)
Supplement: Supplementary file 1 [file ANIE-65-e8926368-s001.pdf]

Supporting Information for

Induction of Methuosis in Breast Cancer Stem Cells by a Cobalt(III) Complex Evokes Immunotherapeutic Response

Jiaxin Fang+,[a] Sijie Wen+,[b] Xiao Feng,[a] Hao Ren,[a] Kuldip Singh,[a] Haihua Xiao,[c]* Lingpu Zhang [c]* and Kogularamanan Suntharalingam [a]*

[a] School of Chemistry, University of Leicester, Leicester, United Kingdom
[b] Beijing National Laboratory for Molecular Sciences, Laboratory of Polymer Physics and Chemistry, Institute of Chemistry Chinese Academy of Sciences, Beijing, P. R. China
[c] Suzhou Institute of Pharmaceutical Innovation, Soochow University, Suzhou City, P. R. China

+ These authors contributed equally.
* To whom correspondence should be addressed:
Email: k.suntharalingam@leicester.ac.uk; hhxiao@suda.edu.cn; lpzhang@suda.edu.cn

Table of Content

Experimental Details
Figure S1. (A) Reaction scheme for the preparation of cobalt(III)-cyclam complex **2** from *trans*-dichloro(cyclam)-cobalt(III) chloride **3** and 1-ethynyl pyrene. (B) X-ray crystal structure of **2**. Ellipsoids are set at 50 % probability level. Hydrogen atoms and the counter-anion have been omitted for clarity.
Figure S2. ^1H NMR spectrum of **1** in DMSO-d_6.
Figure S3. ^1H COSY spectrum of **1** in DMSO-d_6.
Figure S4. $^{19}F\{^1H\}$ NMR spectrum of **1** in DMSO-d_6.
Figure S5. $^{31}P\{^1H\}$ NMR spectrum of **1** in DMSO-d_6.
Figure S6. ATR-FTIR spectra of **1** in the solid form.
Figure S7. High resolution ESI mass spectrum (positive mode) of **1**.
Figure S8. ^1H NMR spectrum of **2** in DMSO-d_6.
Figure S9. ^1H COSY spectrum of **2** in DMSO-d_6.
Figure S10. $^{19}F\{^1H\}$ NMR spectrum of **2** in DMSO-d_6.
Figure S11. $^{31}P\{^1H\}$ NMR spectrum of **2** in DMSO-d_6.
Figure S12. ATR-FTIR spectra of **2** in the solid form.

Figure S13. High resolution ESI mass spectrum (positive mode) of **2**.
Table S1. Crystallographic data for cobalt(III) complexes **1** and **2**.
Table S2. Selected bond lengths (Å) and angles (°) for cobalt(III) complex **1**.
Table S3. Selected bond lengths (Å) and angles (°) for cobalt(III) complex **2**.
Figure S14. UV-Vis spectra of **1** (50 μM) in DMSO over the course of 24 h at 37 ºC.
Figure S15. UV-Vis spectra of **1** (50 μM) in H_2O:DMSO (8:2) over the course of 24 h at 37 ºC.
Figure S16. UV-Vis spectra of **1** (50 μM) in PBS:DMSO (8:2) over the course of 24 h at 37 ºC.
Figure S17. UV-Vis spectra of **1** (50 μM) in Mammary Epithelial Cell Growth Medium (MEGM):DMSO (8:2) over the course of 24 h at 37 ºC.
Figure S18. ESI mass spectrum (positive and negative modes) of **1** (50 μM) in H_2O:DMSO (8:2) after 24 h incubation.
Figure S19. ESI mass spectrum (positive and negative modes) of **1** (50 μM) in H_2O:DMSO (8:2), in the presence of ascorbic acid (250 μM) after 24 h incubation.
Figure S20. ESI mass spectrum (positive and negative modes) of **1** (50 μM) in H_2O:DMSO (8:2), in the presence of glutathione (500 μM) after 24 h incubation.
Figure S21. Emission spectra in acetonitrile (λ_{ex} =356 nm) of **1** (25 μM) and **1** (25 μM) or **2** (25 μM) in the presence of ascorbic acid (250 μM).
Figure S22. The stability profile of **1** (1 mM) in human plasma over the course of 72 h at 37 °C
.**Figure S23.** Representative dose-response curves for the treatment of (A) HMLER and (B) HMLER-shEcad cells with **1** after 72 h incubation. Error bars represent standard deviations.
Figure S24. Representative dose-response curves for the treatment of (A) HMLER and (B) HMLER-shEcad cells with **2** after 72 h incubation. Error bars represent standard deviations.
Figure S25. Representative dose-response curves for the treatment of (A) HMLER and (B) HMLER-shEcad cells with diflunisal after 72 h incubation. Error bars represent standard deviations.
Figure S26. Representative dose-response curves for the treatment of (A) HMLER and (B) HMLER-shEcad cells with 1-ethynyl pyrene after 72 h incubation. Error bars represent standard deviations.
Figure S27. Representative dose-response curves for the treatment of HMLER-shEcad cells with (A) 1:1 mixture of **2** and diflunisal and (B) 1:1:1 mixture of **3**, 1-ethynyl pyrene, and diflunisal after 72 h incubation. Error bars represent standard deviations.
Figure S28. Representative dose-response curves for the treatment of (A) BEAS-2B cells or (B) HEK 293 cells with **1** after 72 h incubation. Error bars represent standard deviations.
Figure S29. Average volume (μm^3) of mammospheres in the absence and presence of cobalt(III) complexes **1**-**3**, diflunisal, 1-ethynyl pyrene, **2** + diflunisal (1:1), **3** + diflunisal + 1-ethynyl pyrene (1:1:1), cisplatin or salinomycin (IC_{20} value for 5 days).
Figure S30. Representative dose-response curves for the treatment of HMLER-shEcad mammospheres with cobalt(III) complexes **1** and **2**, diflunisal, 1-ethynyl pyrene, **2** + diflunisal (1:1), or **3** + diflunisal + 1-ethynyl pyrene (1:1:1).
Figure S31. Representative bright-field images (× 60) of untreated HMLER-shEcad cells, HMLER-shEcad cells treated with 3-methyladenine only (1 mM for 48 h) and

HMLER-shEcad cells co-treated with **1** (2 × IC_{50} value for 48 h) and 3-methyladenine (1 mM for 48 h).

Figure S32. Representative histograms displaying the green fluorescence emitted by CYTO-ID green reagent-stained HMLER-shEcad cells untreated (blue) and treated with rapamycin (1 μM, red) for 24 h.

Figure S33. Representative dose-response curves for the treatment of HMLER-shEcad cells with **1** and cycloheximide (1 μM) or chloroquine (10 μM) or 3-methyladenine (1 mM) after 72 h incubation. Error bars represent standard deviations.

Table S4. IC_{50} values of **1** against HMLER-shEcad cells in the absence and presence of paraptosis (cycloheximide, 1 μM) or autophagy (chloroquine, 10 μM and 3-methyladenine, 1 mM) or methuosis (EIPA, pre-treated with 4 μM for 6 h) inhibitors.

Figure S34. Representative bright-field images (× 60) of untreated HMLER-shEcad cells, HMLER-shEcad cells treated with **1** (2 × IC_{50} value for 48 h) and HMLER-shEcad cells co-treated with **1** (2 × IC_{50} value for 48 h) and EIPA (pre-treated with 4 μM for 6 h).

Figure S35. Representative live cell images (× 60) of HMLER-shEcad cells treated with **1** (4 × IC_{50} value) over a 60 min period.

Figure S36. Representative dose-response curves for the treatment of HMLER-shEcad cells with **1** and EIPA (pre-treated with 4 μM for 6 h) after 72 h incubation. Error bars represent standard deviations.

Figure S37. Protein band intensities corresponding to the immunoblotting analysis shown in Figure 4A.

Figure S38. Immunoblotting analysis of proteins related to the JNK and p38 pathways. Protein expression in HMLER-shEcad cells following treatment with **1** (1-4 μM after 24 h incubation).

Figure S39. (A) Representative histograms displaying the green fluorescence emitted by JC-1 stained HMLER-shEcad cells (blue), and HMLER-shEcad cells treated with **1** (IC_{50} value for 24 h) (red). (B) Representative histograms displaying the green fluorescence emitted by JC-1 stained HMLER-shEcad cells (blue), and HMLER-shEcad cells treated with CCCP (1 μM or 5 μM for 24 h) (red and green).

Figure S40. (A) Representative histograms displaying the green fluorescence emitted by anti-COX-2 Alexa Fluor 488 nm antibody-stained HMLER-shEcad cells treated with LPS (2.5 μg/mL) for 24 h, followed by 48 h in untreated media (blue) or media containing **1** (4 × IC_{50} value, green). (B) Representative histograms displaying the green fluorescence emitted by anti-COX-2 Alexa Fluor 488 nm antibody-stained HMLER-shEcad cells treated with LPS (2.5 μg/mL) for 24 h, followed by 48 h in untreated media (blue) or media containing diflunisal (20 or 40 μM, green and red).

Figure S41. Immunoblotting analysis of the COX-2 protein. Protein expression in HMLER-shEcad cells following treatment with **1** (0.5-2 μM after 72 h incubation).

Figure S42. Protein band intensities corresponding to the immunoblotting analysis shown in Figure S41.

Figure S43. Representative dose-response curves for the treatment of HMLER-shEcad cells pre-treated with lipopolysaccharide (LPS) (2.5 μg/mL for 24 h) and with **1** after 72 h incubation. Error bars represent standard deviations.

Figure S44. (A) Representative histograms displaying the green fluorescence emitted by anti-CRT Alexa Fluor 488 nm antibody-stained HMLER-shEcad cells untreated (blue) and treated with **1** (2 × IC_{50} value) (green) or **1** (4 × IC_{50} value) (red) for

24 h. (B) Representative histograms displaying the green fluorescence emitted by anti-CRT Alexa Fluor 488 nm antibody-stained HMLER-shEcad cells untreated (blue) and treated with **1** (2 × IC_{50} value) (green) or **1** (4 × IC_{50} value) (red) for 6 h. (C) Representative histograms displaying the green fluorescence emitted by anti-CRT Alexa Fluor 488 nm antibody-stained HMLER-shEcad cells untreated (blue) and treated with cisplatin (150 μM) and thapsigargin (7 μM) (green) for 24 h.

Figure S45. Representative histograms displaying the green fluorescence emitted by FITC Annexin V-stained HMLER-shEcad cells untreated (blue) and treated with cisplatin (25 μM) (red) for 72 h.

Figure S46. Representative two-dimensional scatter plots of CellTracker Green-stained HMLER-shEcad cells and CellTracker Orange-stained THP-1 macrophages.

Figure S47. Representative two-dimensional scatter plots of CellTracker Green-stained HMLER-shEcad cells untreated and treated with **1** (5 μM) or **2** (20 μM) or diflunisal (20 μM) for 24 h and then co-cultured with CellTracker Orange-stained THP-1 macrophages for 2 h.

Figure S48. Biodistribution of **NP-1@Cy7.5** in BALB/c mice (n=5) bearing 4T1 breast tumours 3 days post intravenous administration.

Figure S49. The change in body weight of BALB/c mice (n=6) upon intravenous administration of various doses of **NP-1** (0.5-3 mg kg^{-1}) over 12 days.

Figure S50. Representative hematoxylin and eosin (H&E) staining images of heart, liver, and lung tissue taken from BALB/c mice upon intravenous administration of various doses of **NP-1** (0.5-3 mg kg^{-1}) or the vehicle (PBS) over 12 days.

Figure S51. (A) Averaged tumour growth inhibition curves of 4T1 tumour-bearing mice (n = 6 mice), following intravenous administration of the vehicle (PBS) or **1** (1.5 mg Co kg^{-1}). The data is represented as the mean ± SD (n = 6 mice, $^{****}p < 0.0001$, one-way analysis of variance (ANOVA) with Tukey's multiple comparisons test). (B) Excised tumour weight after 14 days upon different treatments. (C) Images of excised tumours from 4T1 tumour-bearing mice after 14 days upon different treatments. (D) Body weight of 4T1 tumour-bearing mice during the efficacy study lasting 14 days.

Figure S52. (A) Average tumour growth inhibition curves of secondary 4T1 tumours in mice (n = 6 mice) following primary tumour establishment by 4T1 cell inoculation, intravenous administration of either vehicle (PBS) or **NP-1** (1.5 mg Co kg^{-1}), surgical resection of the primary tumours after the treatment period, and subsequent rechallenge with a second inoculation of 4T1 cells. The data is represented as the mean ± SD (n = 6 mice, $^{****}p < 0.0001$, one-way analysis of variance (ANOVA) with Tukey's multiple comparisons test). (B) Excised secondary tumour weight measured 14 days after the second inoculation of 4T1 cells. (C) Images of excised secondary tumours collected from 4T1 tumour-bearing mice 14 days following rechallenge with a second inoculation of 4T1 cells.

References

Experimental Details

Materials and Methods. All synthetic procedures were performed under normal atmospheric conditions except specific reactions. ^1H, $^{31}P\{^1H\}$ and $^{19}F\{^1H\}$ NMR were recorded at room temperature on a Bruker Avance 400 spectrometer (^1H 400.0 MHz, ^{31}P 162.0 MHz, ^{19}F 376.5 MHz) with chemical shifts (δ, ppm) reported relative to the solvent peaks of the deuterated solvent. Fourier transform infrared (FTIR) spectra were recorded with an IRAffinity-1S Shimadzu spectrophotometer. UV-vis absorption spectra were recorded on a Cary 3500 UV-Vis spectrophotometer. ICP-MS were measured using a Thermo Scientific ICAP-Qc quadrupole ICP mass spectrometer. Elemental analysis of the compounds prepared was performed commercially by the University of Cambridge. *Trans*-dichloro(cyclam)-cobalt(III) chloride **3** was prepared using a reported protocol.[1] Diflunisal, 1-ethynyl pyrene, silver(I) oxide, sodium hexafluorophosphate, and sodium sulphate were purchased from Sigma-Aldrich and used without further purification. Solvents were purchased from Fisher and used without further purification.

Synthesis of [Co(cyclam)(1-ethynyl pyrene)(diflunisal)]PF_6 (1). Diflunisal (17.5 mg, 0.07 mmol) and **2** (50 mg, 0.07 mmol, synthesis reported below) were dissolved in acetonitrile (25 mL) dried with Na_2SO_4. Silver(I) oxide (90 mg, 0.39 mmol) was added to this solution and the mixture was stirred for 4 days at room temperature. The resulting silver salts were removed by filtration and the filtrate was removed under vacuum. The solid was suspended in acetone (3 mL), filtered, and the filtrate was removed under vacuum. The resultant residue was dissolved in a minimum of DMSO then dispersed in a solution of $NaPF_6$ (100 mg, 0.6 mmol) in water (10 mL). The resulting precipitate was collected and washed with water (3 × 10 mL). The solid was recrystallised from DCM to yield pure **1** as an orange solid (40 mg, 64%); ^1H NMR (400 MHz, DMSO-d_6) δ 12.75 (s, 1H), 8.77 (d, 1H), 8.31-8.27 (m, 3H), 8.25-8.24 (m, 2H), 8.17-8.12 (m, 2H), 8.08 (t, 1H), 8.05 (s, 1H), 7.65-7.57 (m, 2H), 7.37 (ddd, 1H), 7.22 (td, 1H), 7.04 (d, 1H), 6.99 (br s, 2H), 5.63 (br s, 2H), 2.92-2.82 (m, 6H), 2.72-2.55 (m, 8H), 2.44-2.38 (m, 2H), 2.04-1.97 (m, 2H), 1.87-1.81 (m, 1H), 1.74-1.64 (m, 1H); ^{19}F NMR (376.5 MHz, DMSO-d_6) δ -70.15 (d, PF_6), -111.86 (d, F), -114.66 (d, F); ^{31}P NMR (162 MHz, DMSO-d_6) δ -144.20 (sept, PF_6); ATR-FTIR (solid, cm^{-1}): 3255, 1637, 1602, 1567, 1485, 1465, 1433, 1408, 1390, 1362, 1300, 1283, 1256, 1248, 1141, 1100, 1068, 1040, 1023, 970, 878, 859, 845, 826, 811, 785, 714, 663, 555; ESI-MS Calcd. for $C_{41}H_{40}CoF_2N_4O_3$ $[M-PF_6]^+$: 733.2400 a.m.u. Found $[M-PF_6]^+$: 733.2410 a.m.u.; Anal. Calcd. for $C_{41}H_{40}CoF_8N_4O_3P\cdot 0.2H_2O$: C, 55.81; H, 4.62; N, 6.35. Found: C, 55.55; H, 4.54; N, 6.37.

Synthesis of [Co(cyclam)(1-ethynyl pyrene)Cl]PF_6 (2). *Trans*-dichloro(cyclam)-cobalt(III) chloride **3** (100 mg, 0.27 mmol) was dissolved in methanol (10 mL) dried with Na_2SO_4. 1-Ethynyl pyrene (61 mg, 0.27 mmol) dissolved in dry DCM (10 mL) and KOH (20 mg, 0.36 mmol) dissolved in dried methanol (5 mL) were added to the green solution under nitrogen. The mixture was refluxed for 21 h. The resulting suspension was filtered and the filtrate was removed under vacuum. The solid was purified by silica column chromatography DCM:MeOH [9:1]. The purified solid obtained after column chromatography was dissolved in a minimum of DMSO then dispersed in a solution of $NaPF_6$ (100 mg, 0.6 mmol) in water (10 mL). The resulting precipitate was collected and washed with water (3 × 10 mL) to yield pure **2** as an orange solid (59.5 mg, 33%); ^1H NMR (400 MHz, DMSO-d_6) δ 8.77 (d, 1H), 8.30-8.23 (m, 5H), 8.16-8.11 (m, 2H), 8.07 (t, 1H), 5.39 (br s, 4H), 2.87-2.70 (m, 9H), 2.62-2.54 (m, 6H), 2.47-2.45 (m, 1H), 1.94-1.90 (m, 2H), 1.63-1.48 (m, 2H); ^{19}F NMR (376.5 MHz, DMSO-d_6) δ -70.15 (d, PF_6); ^{31}P NMR (162 MHz, DMSO-d_6) δ -144.20 (sept, PF_6); ATR-FTIR (solid, cm^{-1}): 3253, 3241, 3231, 3184, 2966, 1504, 1486, 1462, 1432, 1135, 1298, 1102, 1068, 1056, 1046,

1025, 880, 830, 748, 712, 678, 613, 556; ESI-MS Calcd. for $C_{28}H_{33}ClCoN_4$ $[M-PF_6]^+$: 519.1726 a.m.u. Found $[M-PF_6]^+$: 519.1723 a.m.u.; Anal. Calcd. for $C_{28}H_{33}ClCoF_6N_4P$: C, 50.58; H, 5.00; N, 8.43. Found: C, 50.27; H, 4.96; N, 8.26.

X-ray crystallography. Single crystals of **1** and **2** suitable for X-ray diffraction studies were obtained by vapour diffusion of diethyl ether into acetonitrile solutions of **1** or **2**. Crystals suitable for X-ray diffraction analysis were selected and mounted on a Bruker D8 Quest diffractometer with a Photon III detector and a microfocus source with Cu–Kα radiation (λ = 1.54178) at 150(2) K. Intensities were integrated from data recorded on 1.0° frames by ω rotation. A multiscan method absorption correction (SADABS) with a beam profile was applied.[2] The structures were solved using SHELXS or SHELXT;[3] the datasets were refined by full-matrix least-squares on reflections with $F^2 \geq 2\sigma(F^2)$ values, with anisotropic displacement parameters for all non-hydrogen atoms, and with constrained riding hydrogen geometries, unless otherwise stated;[4] U_{iso}(H) was set at 1.2 times U_{eq} of the parent atom. SHELX was employed through OLEX2 for structure solution and refinement.[5] The structures have been deposited with the Cambridge Crystallographic Data Centre (CCDC 2521598 and 2521599). This information can be obtained free of charge from www.ccdc.cam.ac.uk/data_request/cif.

Measurement of water-octanol partition coefficient (LogP). The LogP value for **1** and **2** was determined using the shake-flask method and UV-vis spectroscopy. The 1-octanol used in this experiment was pre-saturated with water. A DMSO solution of **1** and **2** (10 µL, 10 mM) was incubated with 1-octanol (495 µL) and H_2O (495 µL) in a 1.5 mL tube. The tube was shaken at room temperature for 24 h. The two phases were separated by centrifugation and the content of **1** and **2** in the water and 1-octanol phases was determined by UV-vis spectroscopy.

Human plasma stability. A 10 mM stock solution of **1** in DMSO (100 µL) was added to human plasma (900 µL) and incubated at 37 °C on a mechanical shaker. The final concentration of **1** was 1 mM. Human plasma was obtained from Sigma Aldrich (H4522, 20 mL). At certain time points over a 72 h period, 50 µL of the reaction mixture was aliquoted and mixed with 50 µL of ethanol. The solution was then vortexed for 1 min and centrifuged for 2 min to pellet the proteins present in the human plasma solution. Then 25 µL from the supernatant was taken and added to 25 µL of DMSO and the UV-vis spectrum of the solution was measured to determine the concentration of **1** unbound to proteins in human plasma. Also 25 µL from the supernatant was taken and digested with 25 µL of 70 % nitric acid for 24 h at 65 ºC. The digested sample was then diluted appropriately with MilliQ water and the concentration of unbound cobalt was determined by ICP-MS. The cobalt content in the supernatant directly correlates to intact **1** left in the human plasma solution. The concentration of **1** at 0 h was normalised to 100 % and the percentage left was then plotted against time to obtain the stability profile of **1** in human plasma.

Cell culture. The human mammary epithelial cell lines, HMLER and HMLER-shEcad were kindly donated by Prof. R. A. Weinberg (Whitehead Institute, MIT). BEAS-2B bronchial epithelium, HEK 293 embryonic kidney, and 4T1 mammary carcinoma cell lines were acquired from American Type Culture Collection (ATCC, Manassas, VA, USA). HMLER and HMLER-shEcad cells were maintained in Mammary Epithelial Cell Growth Medium (MEGM) with supplements and growth factors (BPE, hydrocortisone, hEGF, insulin, and gentamicin/amphotericin-B). BEAS-2B cells were cultured in RPMI 1640 medium with 2 mM L-glutamine supplemented with 1% penicillin and 10% fetal bovine serum. HEK 293 cells were cultured in Dulbecco's Modified Eagle's Medium (DMEM) supplemented with 1%

penicillin and 10% fetal bovine serum. 4T1 cells were cultured in RPMI 1640 medium with 1% penicillin and 10% fetal bovine serum. The cells were grown at 310 K in a humidified atmosphere containing 5% CO_2.

Cytotoxicity studies: MTT assay. Exponentially growing cells were seeded at a density of approximately 5 × 10^3 cells per well in 96-well flat-bottomed microplates and allowed to attach for 24 h prior to addition of compounds. Various concentrations of the test compounds (0.0004-100 μM) were added and incubated for 72 h at 37 °C (total volume 200 μL). Stock solutions of the compounds were prepared as 10 mM DMSO solutions and diluted using cell media. Stock solutions of cisplatin were prepared as 5 mM PBS solutions and diluted using cell media. The final concentration of DMSO in each well was <1 %. After 72 h, 20 μL of MTT (4 mg mL^{-1} in PBS) was added to each well and the plates incubated for an additional 4 h at 37 °C. The media/MTT mixture was eliminated and DMSO (100 μL per well) was added to dissolve the formazan precipitates. The optical density was measured at 550 nm using a 96-well multiscanner autoreader. Absorbance values were normalised to (DMSO-containing) control wells and plotted as concentration of compound versus % cell viability. IC_{50} values were interpolated from the resulting dose dependent curves. The reported IC_{50} values are the average of three independent experiments (n = 18).

Mammosphere formation and viability assay. HMLER-shEcad cells (5 × 10^3) were plated in ultralow-attachment 96-well plates (Corning) and incubated in MEGM supplemented with B27 (Invitrogen), 20 ng mL^{-1} EGF and 4 μg mL^{-1} heparin (Sigma) for 5 days. Studies were also conducted in the presence of **1**-**3**, diflunisal, 1-ethynyl pyrene, cisplatin, salinomycin, a 1:1 cocktail of **2** and diflunisal, or a 1:1:1 cocktail of **3**, 1-ethynyl pyrene, and diflunisal (0-133 μM). Mammospheres treated with **1**-**3**, diflunisal, 1-ethynyl pyrene, cisplatin, salinomycin, a 1:1 cocktail of **2** and diflunisal, or a 1:1:1 cocktail of **3**, 1-ethynyl pyrene, and diflunisal (IC_{20} value, 5 days) were counted and imaged using an inverted microscope. The viability of the mammospheres was determined by addition of a resazurin-based reagent, TOX8 (Sigma). After incubation for 16 h, the fluorescence of the solutions was read at 590 nm (λ_{ex} = 560 nm). Viable mammospheres reduce the amount of the oxidised TOX8 form (blue) and concurrently increase the amount of the fluorescent TOX8 intermediate (red), indicating the degree of mammosphere cytotoxicity caused by the test compound. Fluorescence values were normalised to DMSO-containing controls and plotted as concentration of test compound versus % mammosphere viability. IC_{50} values were interpolated from the resulting dose dependent curves. The reported IC_{50} values are the average of three independent experiments, each consisting of two replicates per concentration level (n = 6).

Cellular uptake and fractionation studies. To measure the cellular uptake of **1** about 1 million HMLER-shEcad cells were treated with **1** (1 μM) at 37 °C for 24 h. After incubation, the media was removed, the cells were washed with PBS (2 mL × 3) and harvested. The number of cells was counted at this stage, using a haemocytometer. This mitigates any cell death induced by **1** at the administered concentration and experimental cell loss. Half of the cellular pellet was dissolved in 65% HNO_3 (250 μL) overnight. Half of the cellular pellet was used to determine the cobalt content in the cytoplasmic, nuclear, and membrane fractions. The ThermoScientific NE-PER Nuclear and Cytoplasmic Extraction Kit was used to extract and separate the cytoplasmic, nuclear, and membrane fractions. The fractions were dissolved in 65% HNO3 (250 μL final volume) overnight. All samples were diluted 17-fold with water and analysed using inductively coupled plasma mass spectrometry (ICP-MS, Thermo Scientific ICAP-Qc quadrupole ICP mass spectrometer). Cobalt levels are expressed as mass of Co (ng) per million cells. Results are presented as the mean of four determinations for each data point.

CYTO-ID assay. HMLER-shEcad cells were seeded into a 6-well plate (at a density of 5 × 10^5 cells/ mL) and the cells were incubated at 37 °C overnight. The cells were dosed with **1** (IC_{50} value and 2 × IC_{50} value) or rapamycin (1 μM) for 24 h. Following this incubation, cells were washed with PBS (500 μL) and harvested from adherent cultures by trypsinisation. Cells were centrifuged and the supernatant was removed. The cells were resuspended in 1 × CTYO-ID assay buffer (250 μL) and the CYTO-ID dye was added (250 μL). After 30 minutes incubation, the cells were centrifuged, the supernatant removed and the cells resuspended in PBS (500 μL) twice. Then, the cells were resuspended in the CYTO-ID assay buffer and analysed using a FACSCanto II flow cytometer (BD Biosciences) (10,000 events per sample were acquired) at the University of Leicester FACS Facility. Cell populations were analysed using Floreada.io.

Immunoblotting analysis. HMLER-shEcad cells (5 × 10^6) were incubated with **1** (1-4 μM for 24 h or 0.5-2 μM for 72 h) at 37 ºC. HMLER-shEcad cells were harvested and isolated as pellets. SDS-PAGE loading buffer (64 mM Tris-HCl (pH 6.8), 9.6% glycerol, 2% SDS, 5% β-mercaptoethanol, 0.01% bromophenol blue) was added to the pellets, and this was incubated at 95 ºC for 10 min. Lysates were resolved by 4-20 % sodium dodecylsulphate polyacylamide gel electrophoresis (SDS-PAGE; 200 V for 25 min) followed by electro transfer to polyvinylidene difluoride membrane, PVDF (350 mA for 1 h). Membranes were blocked in 5% (w/v) non-fat milk in PBST (PBS/0.1% Tween 20) and incubated with the appropriate primary antibodies (Cell Signalling Technology). After incubation with horseradish peroxidase-conjugated secondary antibodies (Cell Signalling Technology), immune complexes were detected with the ECL detection reagent (BioRad) and analysed using a chemiluminescence imager (Bio-Rad ChemiDoc Imaging System).

Cell cycle analysis. HMLER-shEcad cells (1.5 × 10^6 per well) grown in six-well plates were incubated with and without **1** (2 × IC_{50} value) for 24-72 h at 37 °C. Cells were harvested from adherent cultures by trypsinisation and combined with all detached cells from the incubation medium to assess total cell viability. Following centrifugation at 1000 rpm for 5 min, cells were washed with PBS and then fixed with 70% ethanol in PBS. Fixed cells were collected by centrifugation at 2500 rpm for 3 min, washed with PBS, and centrifuged as before. Cellular pellets were resuspended in 50 μg/mL PI (Sigma) in PBS for nucleic acids staining and treated with 100 μg/mL RNaseA (Sigma). DNA content was measured using a FACSCanto II flow cytometer (BD Biosciences) (10,000 events per sample were acquired) at the University of Leicester FACS Facility. Cell cycle profiles were analysed using Floreada.io.

Intracellular ROS assay. HMLER-shEcad cells (5 × 10^3) were seeded in each well of a 96-well plate. After incubating the cells overnight, they were treated with **1** (2 × IC_{50} value or 4 × IC_{50} value for 0.5-24 h), and incubated with 6-carboxy-2′,7′-dichlorodihydrofluorescein diacetate (20 μM) for 30 min. The intracellular ROS level was determined by measuring the fluorescence of the solutions in each well at 529 nm (λ_{ex} = 504 nm).

JC-1 assay. The JC-1 Mitochondrial Membrane Potential Assay Kit (Cayman) was used. The manufacturer's protocol was followed to carry out this experiment. Untreated and treated HMLER-shEcad cells (1 × 10^6 cells) grown in six-well plates were treated with the JC-1 staining solution for 30 min (100 μL/mL of cell media). Following staining, the cells were harvested, and analysed using a FACSCanto II flow cytometer (BD Biosciences) (10,000 events per sample were acquired) at the University of Leicester FACS Facility. Cell cycle profiles were analysed using Floreada.io.

COX-2 expression assay. HMLER-shEcad cells were seeded in 6-well plates (at a density of 5×10^5 cells/ mL) and the cells were allowed to attach overnight. The cells were treated with lipopolysaccharide (LPS) (2.5 μg/mL for 24 h), and then treated with **1** (4 × IC_{50} value) or diflunisal (20-40 μM) and incubated for a further 48 h. The cells were then harvested by trypsinisation, fixed with 4% paraformaldehyde (at 37 °C for 10 min), permeabilised with ice-cold methanol (for 30 min), and suspended in PBS (200 μL). The Alexa Fluor® 488 nm labelled anti-COX-2 antibody (5 μL) was then added to the cell suspension and incubated in the dark for 1 h. The cells were then washed with PBS (1 mL) and analysed using a FACSCanto II flow cytometer (BD Biosciences) (10,000 events per sample were acquired) at the University of Leicester FACS Facility. The FL1 channel was used to assess COX-2 expression. Cell populations were analysed using Floreada.io.

ATP assay. HMLER-shEcad cells (5×10^3 cells /well) were seeded in a 96-well plate and incubated overnight. The cells were then treated with **1** (2-4 μM for 24 h) or cisplatin (50 μM, positive control) for 24 h at 37 °C. The media was carefully extracted and transferred into a white-walled opaque 96-well plate, and a luciferin-based ENLITEN ATP Assay Kit (Promega) was used to measure the relative amount of ATP released into the supernatant.

HMGB-1 release. HMLER-shEcad cells (1×10^6 cells) were incubated with **1** (1-4 μM) for 24 h at 37 °C. Cells were collected in full and added to SDS-PAGE loading buffer (64 mM Tris-HCl (pH 6.8), 9.6% glycerol, 2% SDS, 5% β-mercaptoethanol, 0.01% Bromophenol Blue) and incubated at 95 °C for 10 min. The HMGB-1 content was probed by immunoblotting analysis as described above. The anti-HMGB-1 antibody (Cell Signalling Technology) was used in this experiment.

CRT cell surface exposure. Flow cytometry was used to analyse cell surface CRT exposure. HMLER-shEcad cells were seeded into a 6-well plate (at a density of 5×10^5 cells/ mL) and the cells were incubated at 37 °C overnight. The cells were treated with **1** (2 × IC_{50} value or 4 × IC_{50} value) for 24 h or co-treated with cisplatin (150 μM) with thapsigargin (7 μM) for 24 h at 37 °C. The cells were then harvested by trypsinization and collected by centrifugation. The pellet was suspended in PBS (500 μL), and after the addition of the Alexa Fluor® 488 nm labelled anti-CRT antibody (2 μL), the cells were incubated in the dark for 30 minutes. The cells were then washed with PBS (1 mL) and analysed using a FACSCanto II flow cytometer (BD Biosciences) (10,000 events per sample were acquired) at the University of Leicester FACS Facility. The FL1 channel was used to assess CRT cell surface exposure. Cell populations were analysed using Floreada.io.

PS cell surface exposure. Flow cytometry was used to analyse cell surface PS exposure. HMLER-shEcad cells were seeded into a 6-well plate (at a density of 5×10^5 cells/ mL) and the cells were incubated at 37 °C overnight. The cells were treated with **1** (2 × IC_{50} value or 4 × IC_{50} value) or with cisplatin (25 μM) for 72 h at 37 °C. The cells were then harvested by trypsinisation and collected by centrifugation. The pellets were suspended in 1× Annexin binding buffer (100 μL) (10 mM HEPES, 140 mM NaCl, 2.5 mM $CaCl_2$, pH 7.4), then 5 μL of FITC Annexin V was added to each sample and incubated at room temperature for 15 min. After which more 1× Annexin binding buffer (400 μL) was added while gently mixing. The cells were analysed using a FACSCanto II flow cytometer (BD Biosciences) (10,000 events per sample were acquired) at the University of Leicester FACS Facility. The FL1 channel was used to assess Annexin V binding. Cell populations were analysed using Floreada.io.

Phagocytosis assay. HMLER-shEcad cells were seeded into a 6-well plate (at a density of 5 × 10^5 cells/ mL) and the cells were incubated at 37 °C overnight. The cells were stained with CellTracker Green (30 min) and washed with MEGM media. The cells were then treated with **1** (5-10 µM) or **2** (20 µM) or diflunisal (20 µM) or cisplatin (150 µM) with thapsigargin (7 µM) for 24 h at 37 °C. Then macrophages, obtained by differentiating THP-1 cells with phorbol 12-myristate 13-acetate (100 nM for 72 h) and pre-stained with CellTracker Orange for 30 min and washed with RPMI 1640 media, were added to the HMLER-shEcad cells (at a density of 1 × 10^5 cells/ mL). After 2 h, phagocytosis was assessed by flow cytometry, using a FACSCanto II flow cytometer (BD Biosciences) (10,000 events per sample were acquired) at the University of Leicester FACS Facility. The FL1 channel was used to assess the CellTracker Green-stained HMLER-shEcad cell population and the FL4 channel was used to assess the CellTracker Orange-stained THP-1 macrophage population. Cell populations were analysed using Floreada.io.

Preparation and characterisation of NP-1 and NP-2. Distearoyl phosphatidylethanolamine-polyethylene glycol 2000 (DSPE-PEG_{2000}) was purchased from Bide Pharmatech Ltd., Shanghai, China. Cobalt(III) complexes **1** or **2** (10 mg) and DSPE-PEG_{2000} (100 mg) were dissolved in 2 mL of DMSO, which was then added dropwise into 18 mL of water under continuous agitation. Then, the mixture was dialysed using a dialysis bag (MWCO: 8000-14000 Da). After 48 h, the mixture was concentrated through ultrafiltration, followed by filtration using a 0.22 µm syringe-driven filter to give **NP-1** or **NP-2**. The morphology and size of **NP-1** or **NP-2** were measured by transmission electron microscope (TEM, Hitachi HT 7700, Japan). The size and polydispersity index of **NP-1** or **NP-2** were measured by dynamic light scattering on an ALV/CGS-3 goniometry system (ALV, Germany). The concentration of cobalt in **NP-1** or **NP-2** was quantitatively determined by a Graphite Furnace Atomic Absorption Spectrometer.

Preparation and characterisation of NP-1@Cy7.5. Cobalt(III) complex **1** (1 mg), Cy7.5 (100 µg), and DSPE-PEG_{2000} (50 mg) were dissolved in 1 mL of DMSO, which was then added dropwise into 9 mL of water under continuous agitation. Then, the mixture was dialysed using a dialysis bag (MWCO: 8000-14000 Da). After 48 h, the mixture was concentrated through ultrafiltration, followed by filtration using a 0.22 µm syringe-driven filter to give **NP-1@Cy7.5**.

Tissue biodistribution of NP-1@Cy7.5. All animal experiments were approved by the Peking University Institutional Animal Care and Use Committee (LA2021316). The breast pads of female BALB/c mice were subcutaneously injected with 4T1 cells (2×10^6). After 7 days, the tumour volumes of the mice reached approximately 50 mm^3. The mice (n=5) were intravenously injected with **NP-1@Cy7.5** (0.2 mg Co kg^{-1}). Then mice were imaged using an Imaging System instrument (IVIS Spectrum CT, PerkinElmer, $\lambda_{ex}/\lambda_{em}$ = 745 nm/ 840 nm) at 1 h, 6 h, 12 h, 24 h, 48 h, and 72 h. After 72 h, various tissues (heart, liver, spleen, lung, kidney, intenstines, tumour) was excised from euthanised mice (n=5). The tissues were imaged using an Imaging System instrument (IVIS Spectrum CT, PerkinElmer, $\lambda_{ex}/\lambda_{em}$ = 745 nm/ 840 nm).

Maximum tolerated dose studies. All animal experiments were approved by the Peking University Institutional Animal Care and Use Committee (LA2021316). BALB/c mice were intravenously injected with various doses of **NP-1** (0.5, 1.5 or 3 mg Co kg^{-1} in PBS) every 2 days over a 12 day period. The dose refers to the metal in each treatment. The body weight of the mice was monitored every 2 days for 12 days. At the end of the treatment period, all mice were sacrificed. Various tissues (heart, liver, and lung) were fixed in freshly prepared paraformaldehyde (4% in PBS) for 24 h and processed for paraffin sectioning. Tissue sections of 5 μm were stained with hematoxylin and eosin (H&E) and used for histological examination.

Tumour growth inhibition studies. All animal experiments were approved by the Peking University Institutional Animal Care and Use Committee (LA2021316). 4T1 cells are known to contain a high proportion of $CD44^{+}/CD24^{-}$ breast CSCs, making them particularly well suited for evaluating anti-CSC activity in a murine model.[6] The breast pads of female BALB/c mice were subcutaneously injected with 4T1 cells (2×10^{6}). After 10 days, the tumour volumes of the mice reached approximately 50 mm^3. The mice were randomly divided into five groups (n=5 or 6). Then the mice were intravenously injected with PBS or cisplatin (1.5 mg Pt kg^{-1} in PBS) or **NP-1** (1.5 mg Co kg^{-1} in PBS) or **1** (1.5 mg Co kg^{-1} in PBS) or **NP-2** (1.5 mg Co kg^{-1} in PBS) every 2 days for a further 8 days and the body weight and tumour volume was monitored every 2 days for 14 days. The dose refers to the respective metal in each treatment. Tumour volume was calculated by the following formula: volume = (length × $width^2$)/ 2. At the end of the study, all mice were sacrificed, and the tumours were collected, weighed, and imaged. Based on priori power calculations (via http://clincalc.com/Stats/SampleSize.aspx), the sample sizes used in the tumour growth inhibition studies were sufficient to detect a 75% reduction in tumour growth with 80% statistical power, using $\alpha = 0.05$ and $\beta = 0.15$.

Immunotherapeutic efficacy studies. All animal experiments were approved by the Peking University Institutional Animal Care and Use Committee (LA2021316). The breast pads of female BALB/c mice were subcutaneously injected with 4T1 cells (2×10^{6}). After 10 days, the tumour volumes of the mice reached approximately 50 mm^3. The mice were randomly divided into four groups (n=5). Then the mice were intravenously injected with PBS or cisplatin (1.5 mg Pt kg^{-1} in PBS) or **NP-1** (1.5 mg Co kg^{-1} in PBS) or **NP-2** (1.5 mg Co kg^{-1} in PBS) every 2 days for a further 8 days. The dose refers to the respective metal in each treatment. Mice were sacrificed after 14 days. The tumour and the tumour-draining lymph nodes were excised from euthanised mice and homogenised to produce single-cell suspensions. The single-cell suspensions were incubated with various antibodies for the immune cells (mature dendritic cells, T-cells, M1/M2 macrophages, myeloid-derived suppressor cells). For the detection of mature dendritic cells, cells collected from tumour-draining lymph nodes were stained with anti-CD11c-PE, anti-CD80-FITC, and anti-CD86-APC antibodies. For analysis of M1 and M2 macrophages, cells collected from tumours were stained with anti-F4/80-PE, anti-CD80-FITC, and anti-CD206-APC antibodies. For analysis of T cells, cells collected from tumours were stained with anti-CD3-PE, anti-CD4-APC, and anti-CD8-FITC antibodies. For analysis of myeloid-derived suppressor cells, cells collected from tumours were stained with anti-CD11b-PE, anti-CD45-PC5.5, anti-MHC-II-APC, and anti-Gr-1-FITC antibodies.

Tumour rechallenge studies. All animal experiments were approved by the Peking University Institutional Animal Care and Use Committee (LA2021316). The breast pads of female BALB/c mice were subcutaneously injected with 4T1 cells (2×10^{6}). After 10 days, the tumour volumes of the mice reached approximately 50 mm^3. The mice were randomly divided into two groups (n=6). Then the mice were intravenously injected with PBS or **NP-1** (1.5 mg Co kg^{-1} in PBS) every 2 days for a further 8 days. The dose refers to the metal in each treatment.

After the treatment period, the tumors were surgically removed, and the mice were rechallenged with a second subcutaneous inoculation of 4T1 cells (2×10^6). Tumour volume of the secondary tumour was then monitored and recorded over 14 days. Tumour volume was calculated by the following formula: volume = (length × width^2)/ 2. At the end of the study, all mice were sacrificed, and the tumours were collected, weighed, and imaged.

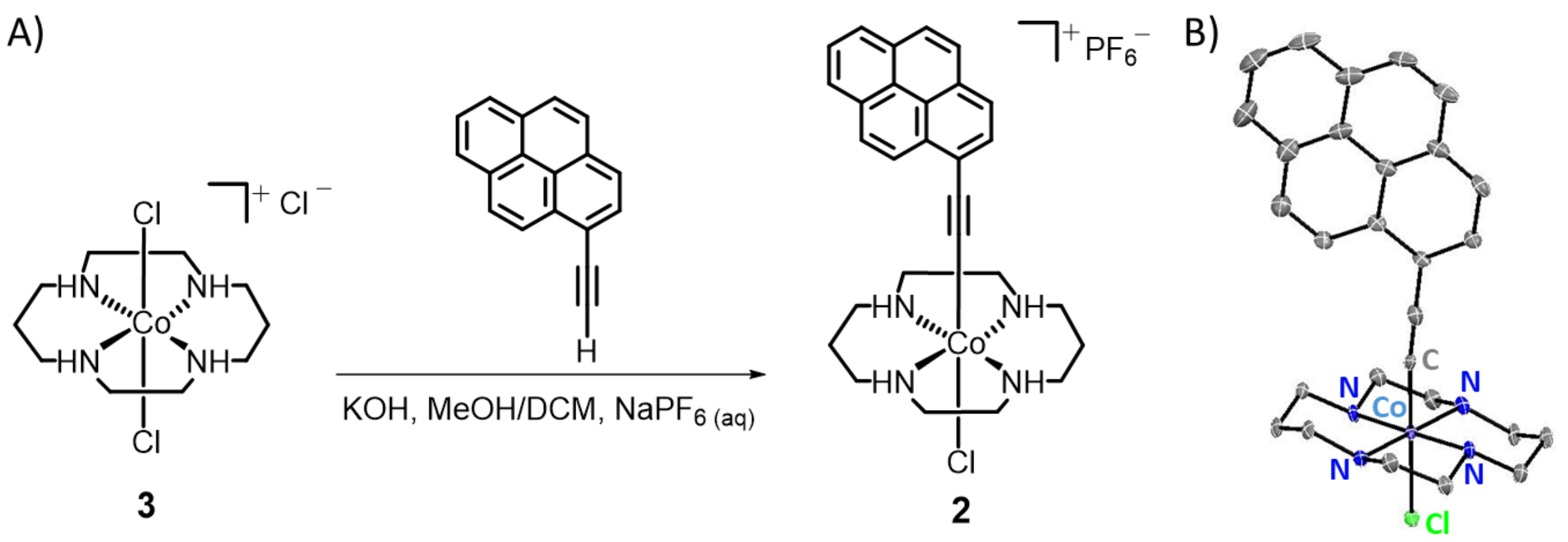

Figure S1. (A) Reaction scheme for the preparation of cobalt(III)-cyclam complex **2** from *trans*-dichloro(cyclam)-cobalt(III) chloride **3** and 1-ethynyl pyrene. (B) X-ray crystal structure of **2**. Ellipsoids are set at 50 % probability level. Hydrogen atoms and the counter-anion have been omitted for clarity.

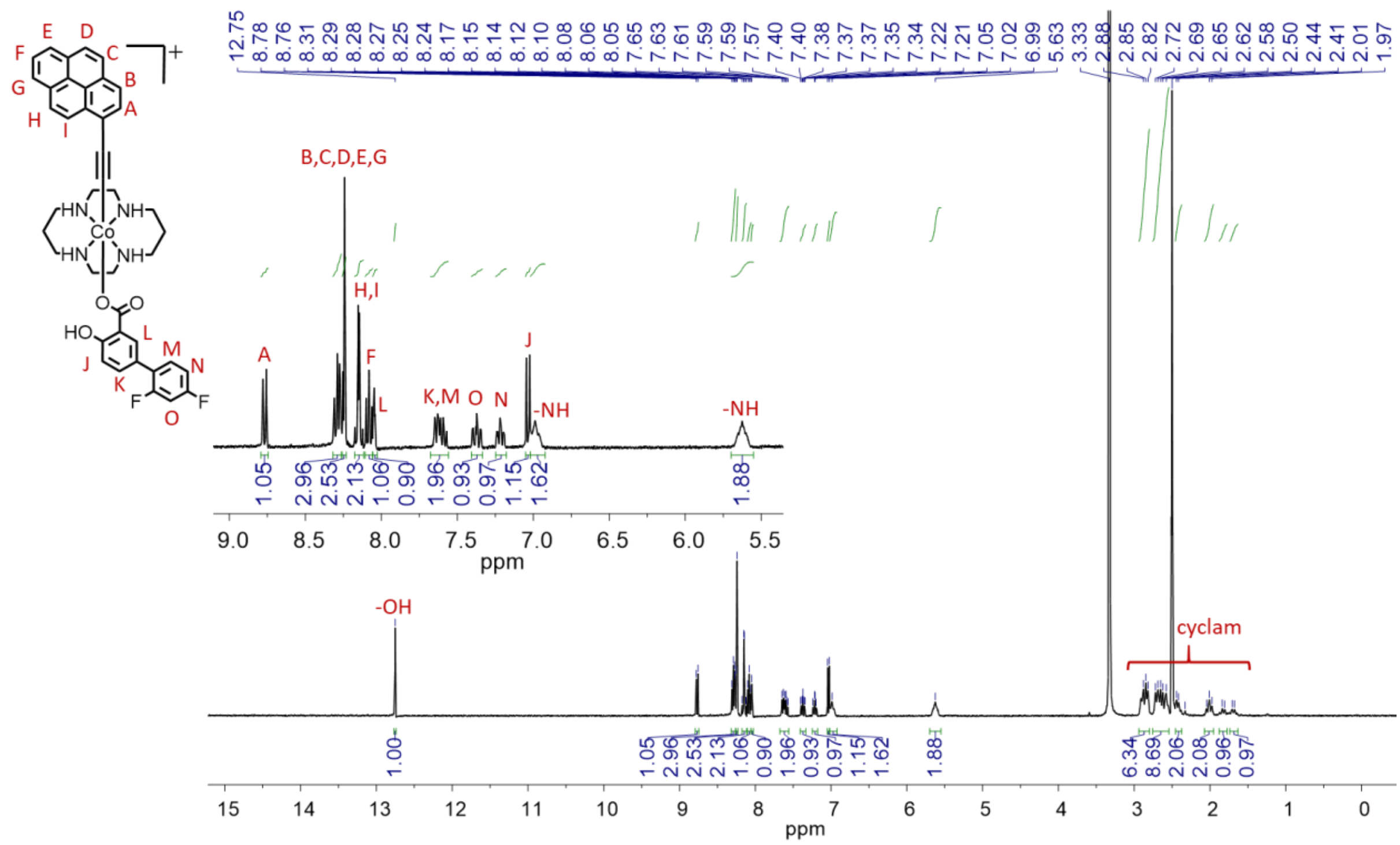

Figure S2. ^{1}H NMR spectrum of **1** in DMSO-d_6.

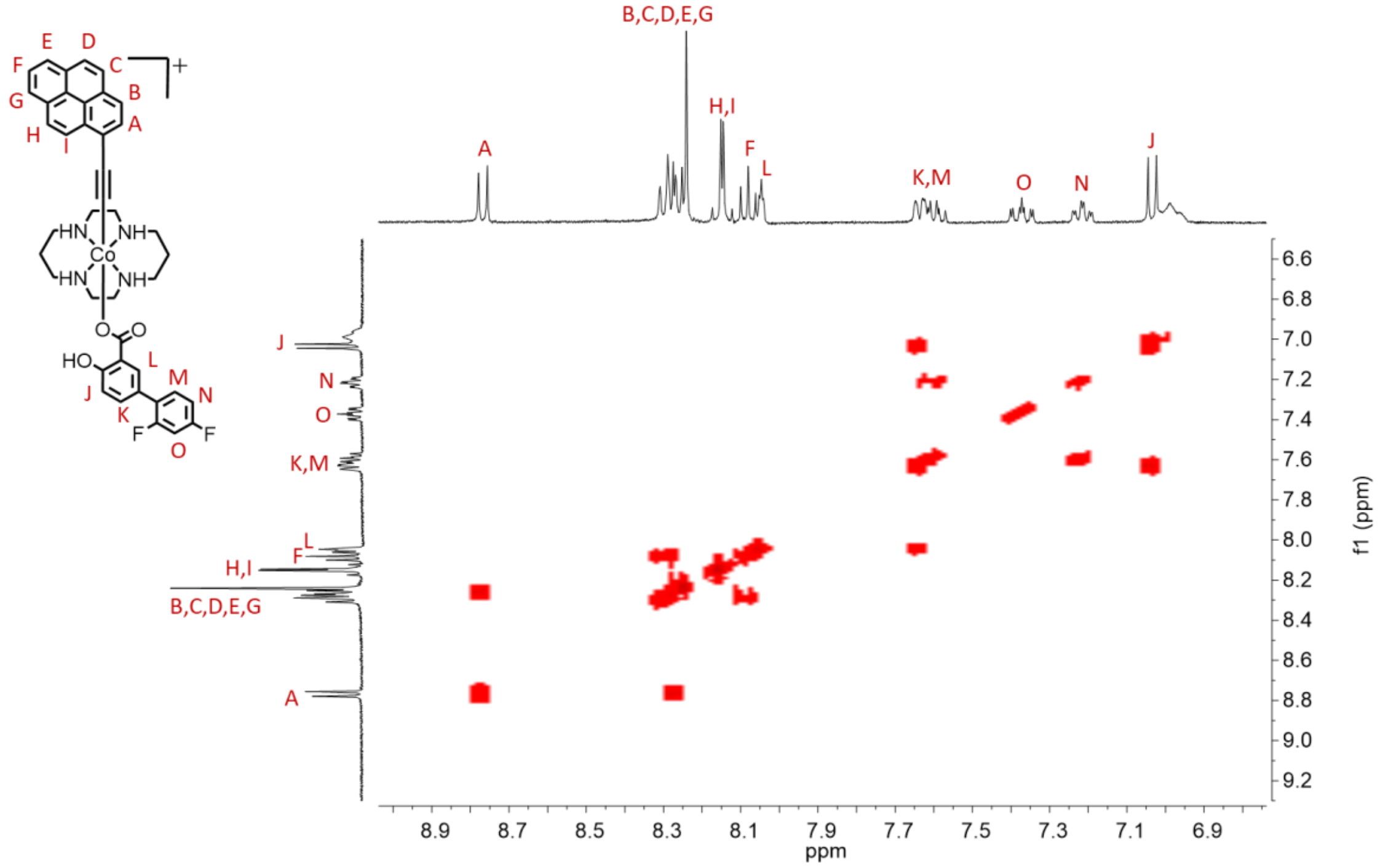

Figure S3. ^{1}H COSY spectrum of **1** in DMSO-d$_6$.

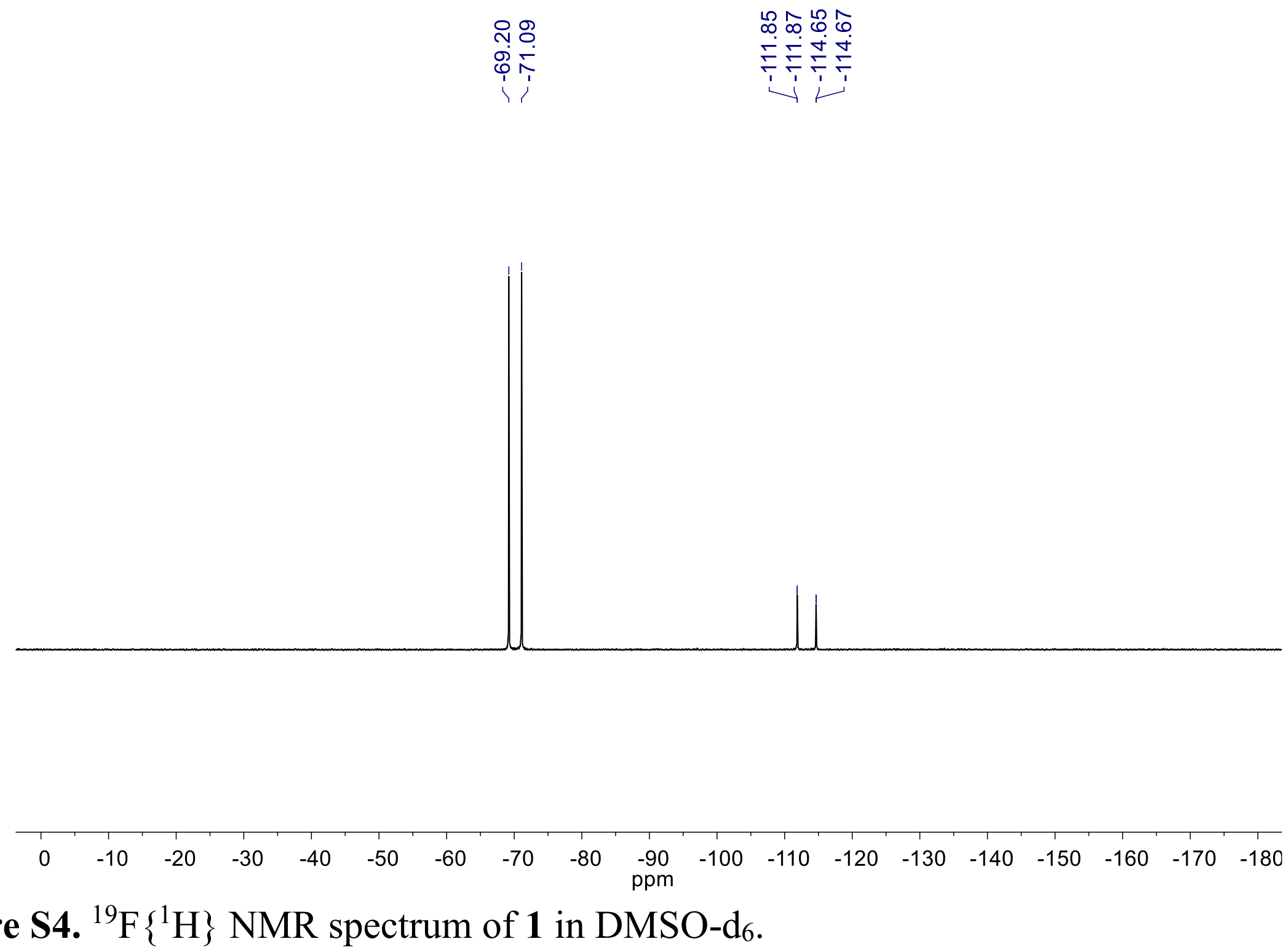

Figure S4. ^{19}F{^{1}H} NMR spectrum of **1** in DMSO-d$_6$.

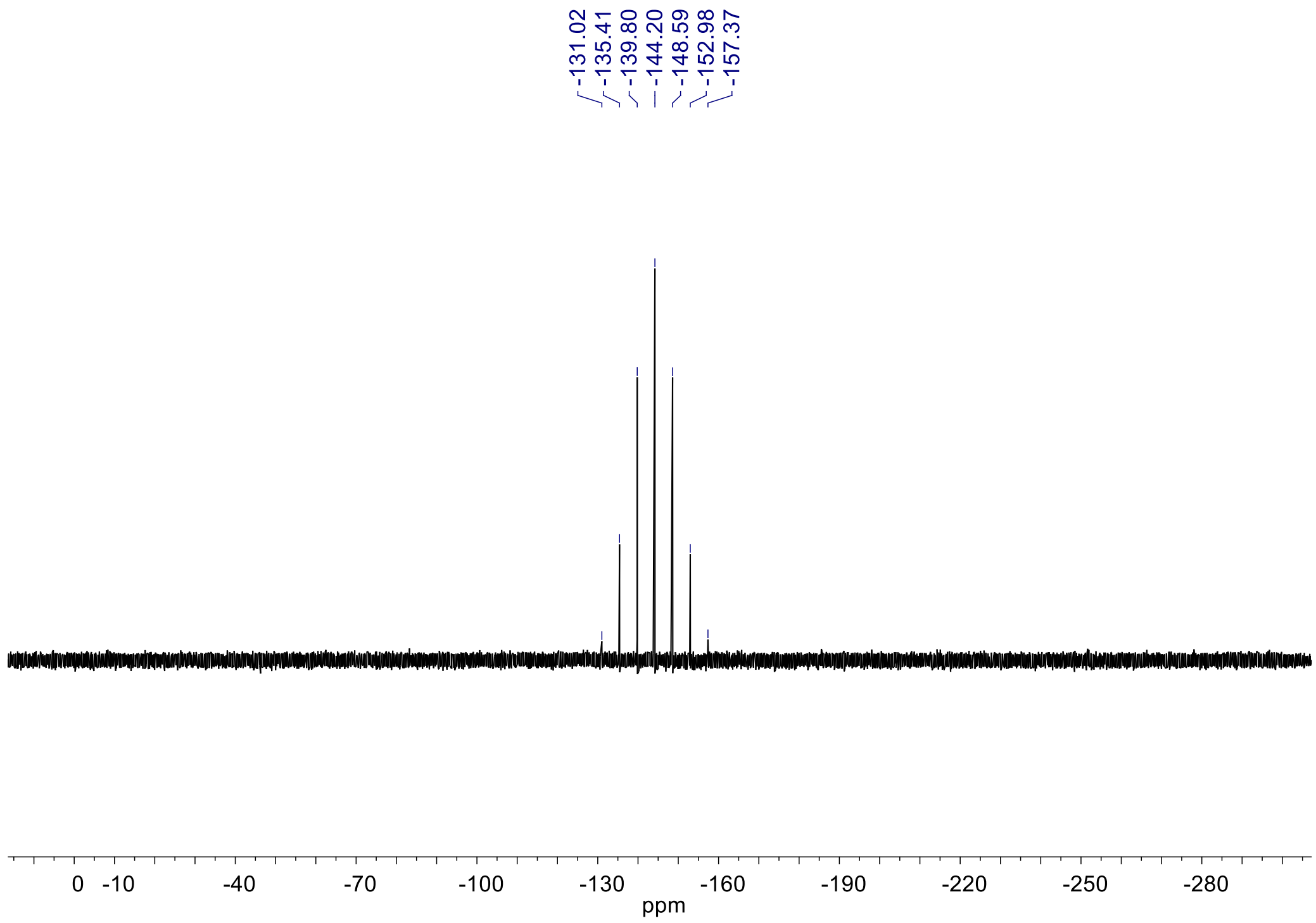

Figure S5. $^{31}P\{^{1}H\}$ NMR spectrum of **1** in DMSO-d_6.

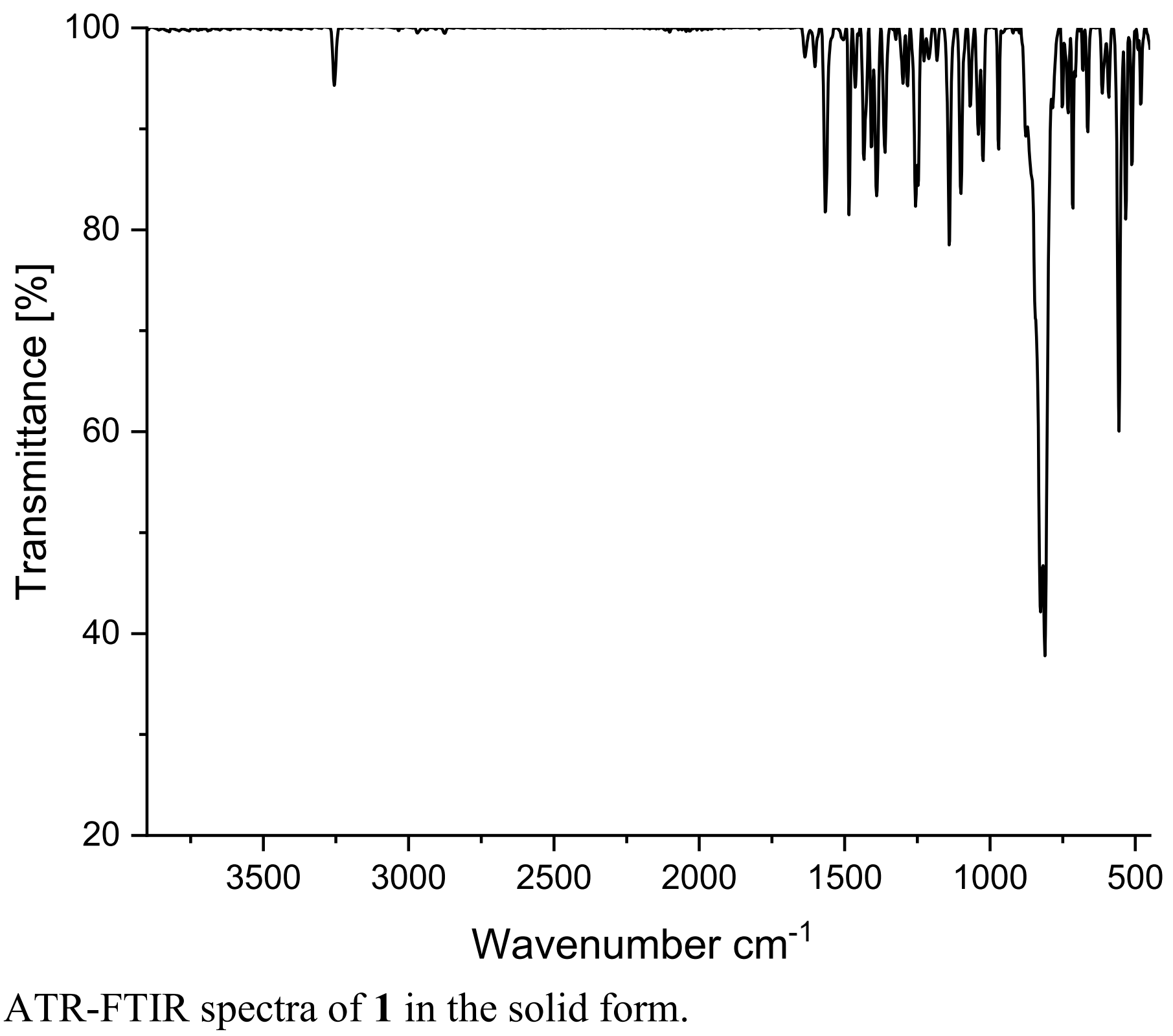

Figure S6. ATR-FTIR spectra of **1** in the solid form.

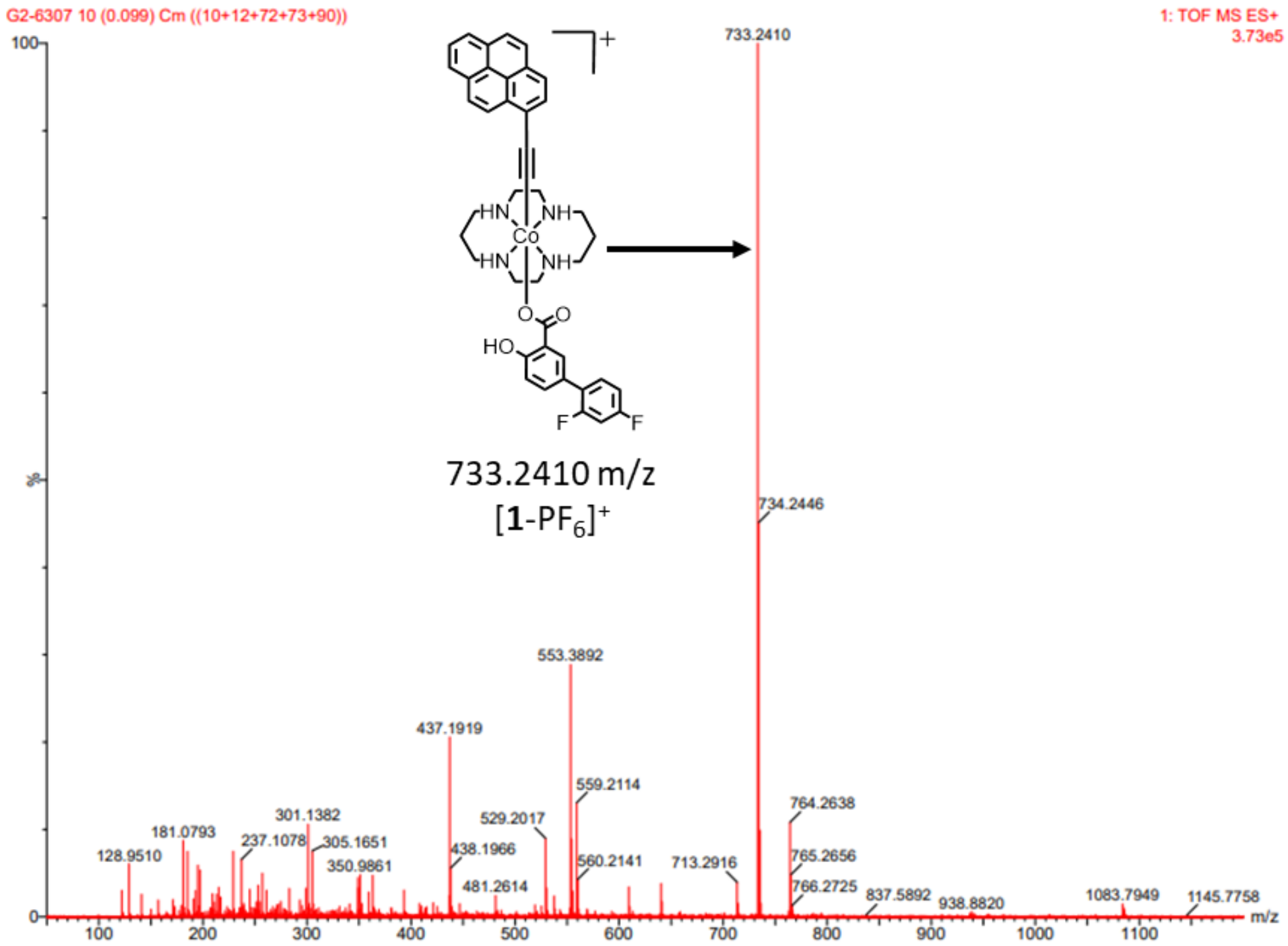

Figure S7. High resolution ESI mass spectrum (positive mode) of **1**.

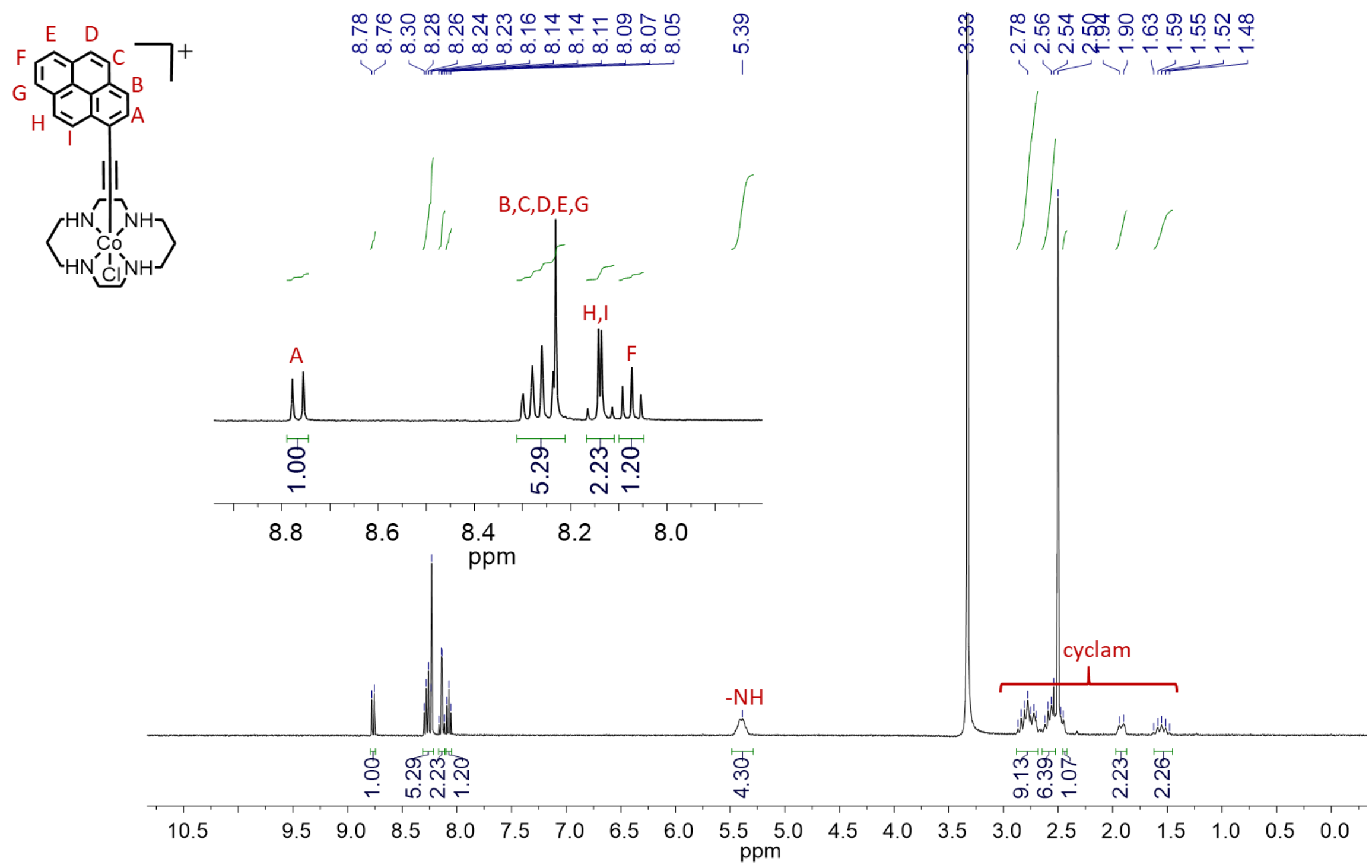

Figure S8. ^{1}H NMR spectrum of **2** in DMSO-d_6.

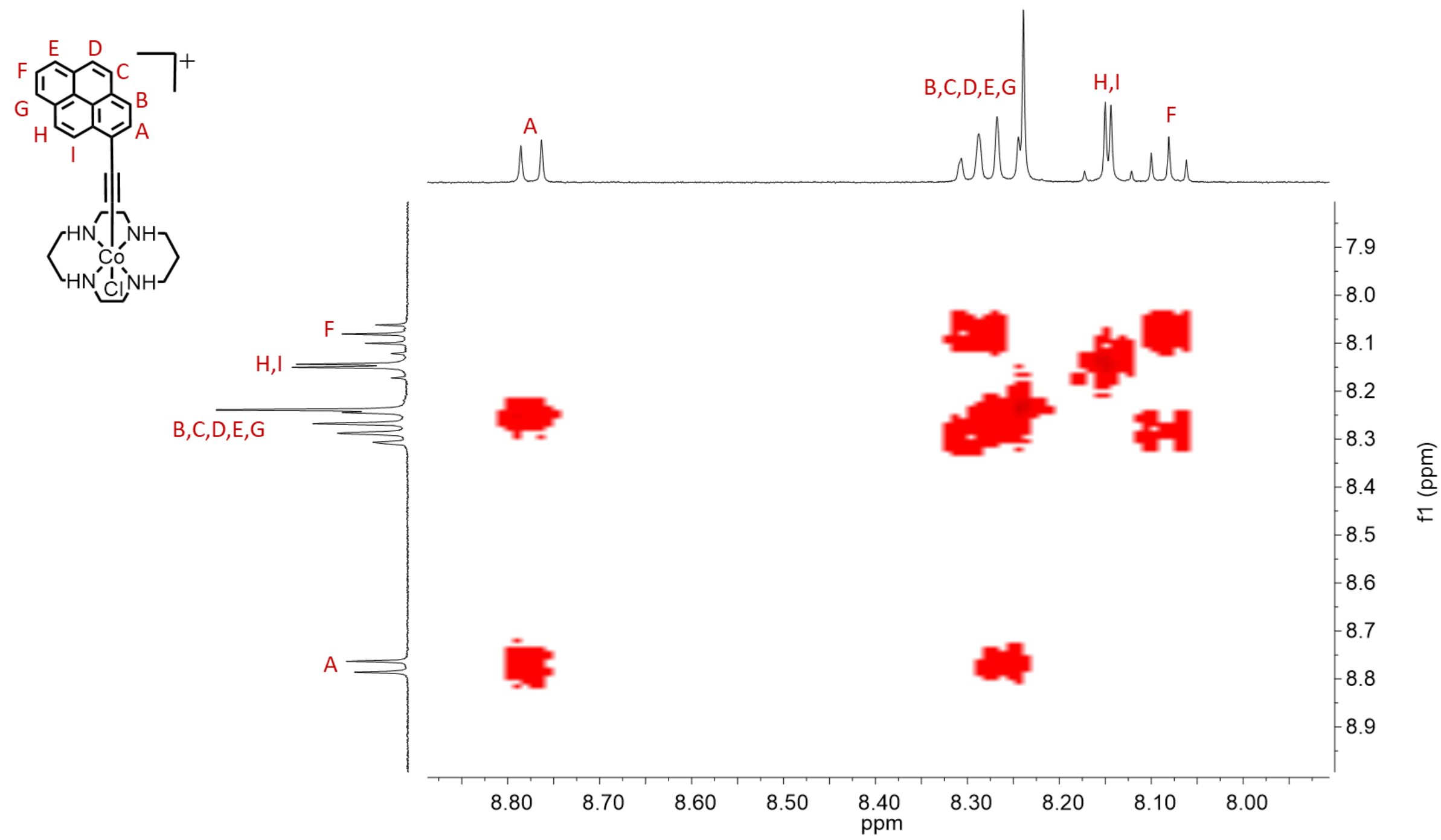

Figure S9. ^{1}H COSY spectrum of **2** in DMSO-d$_6$.

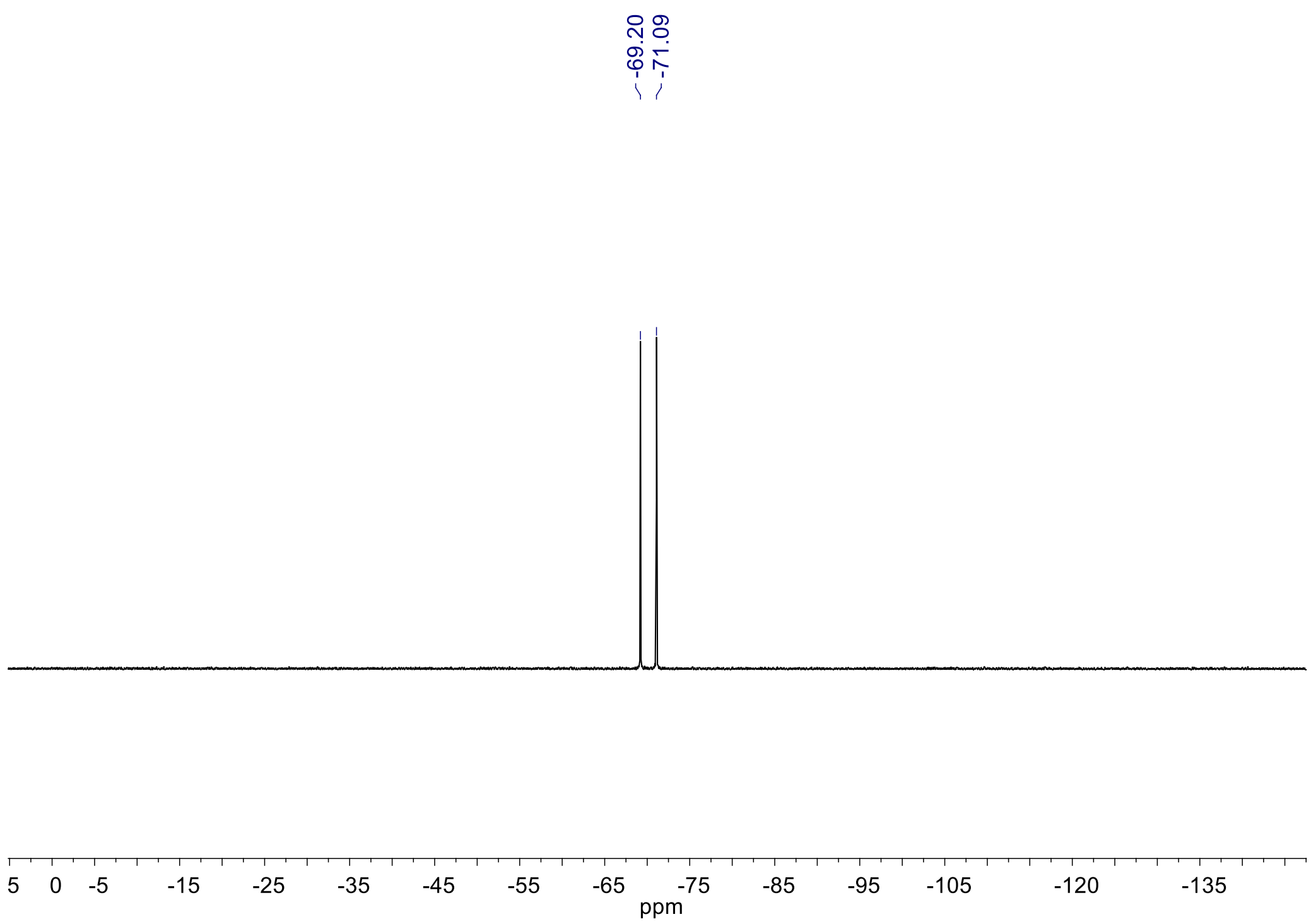

Figure S10. ^{19}F{^{1}H} NMR spectrum of **2** in DMSO-d$_6$.

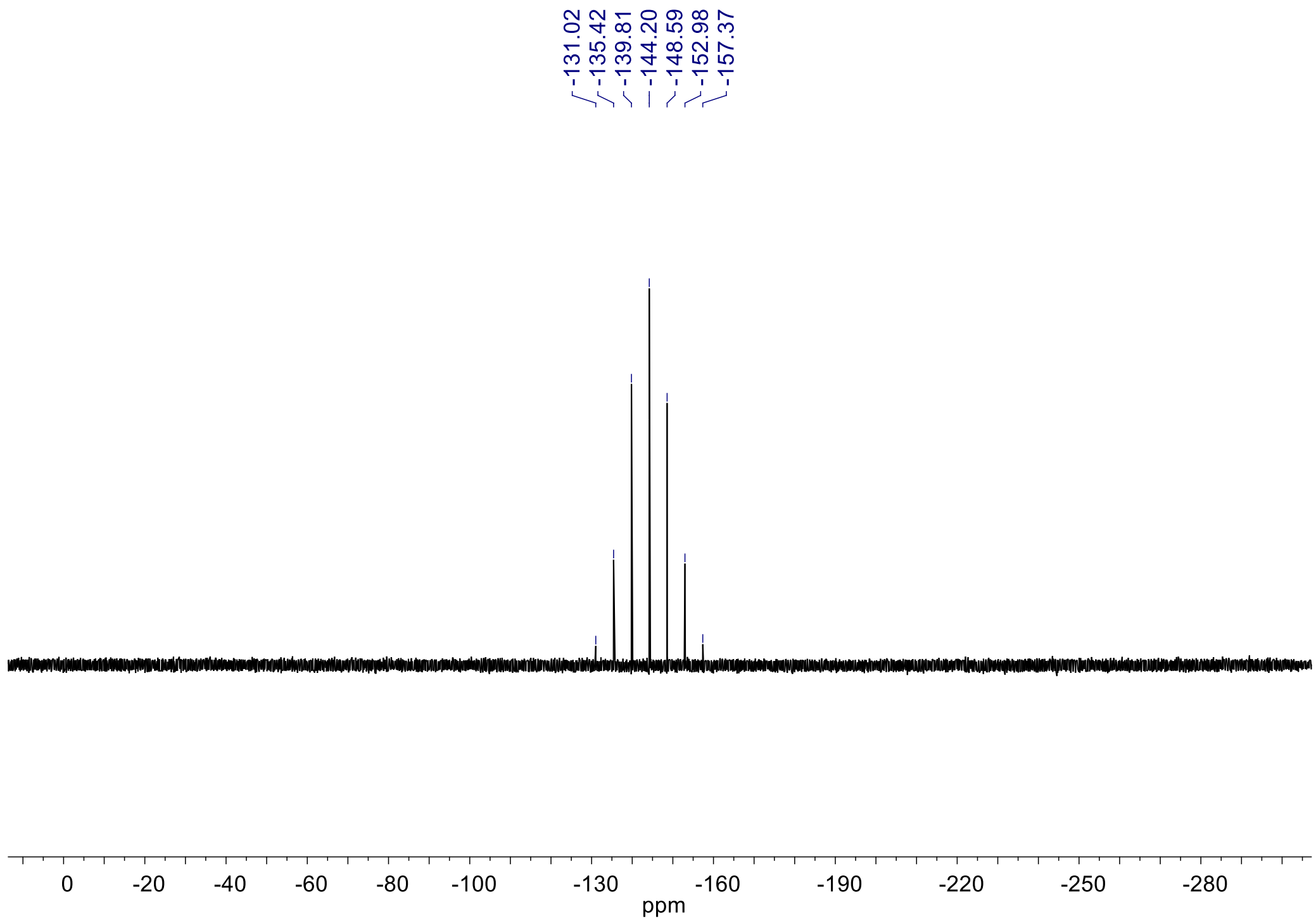

Figure S11. $^{31}P\{^{1}H\}$ NMR spectrum of **2** in DMSO-d$_6$.

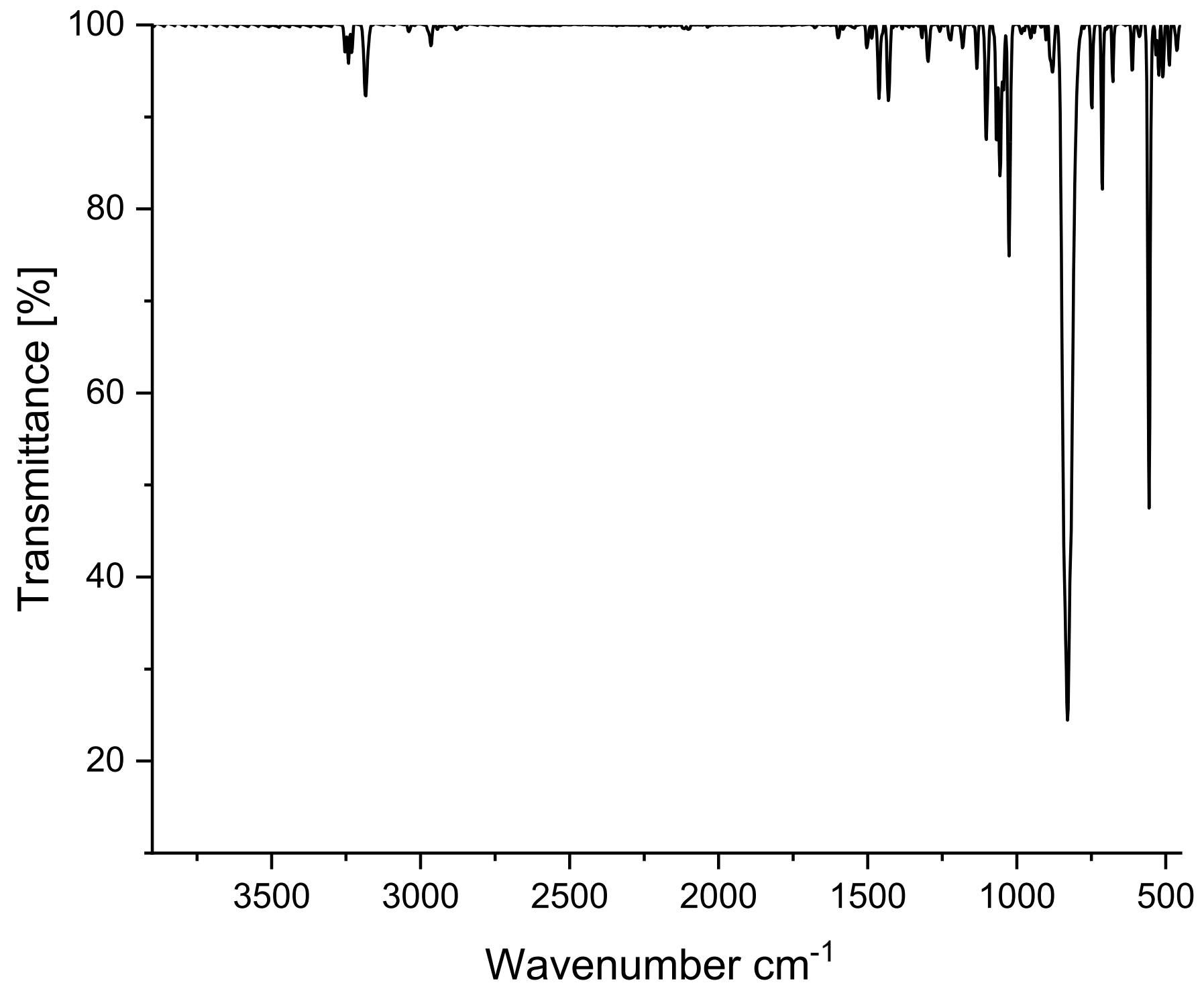

Figure S12. ATR-FTIR spectra of **2** in the solid form.

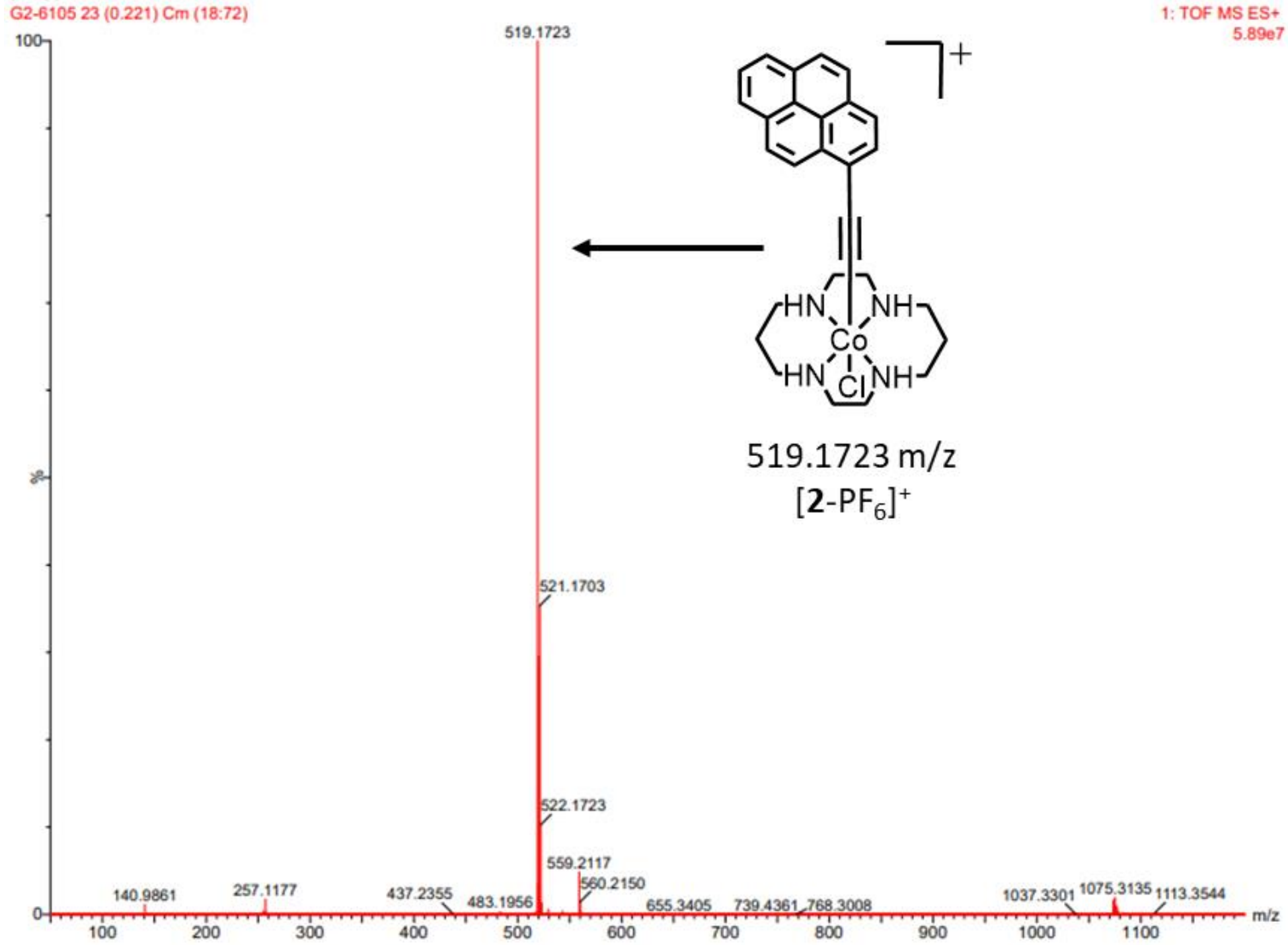

Figure S13. High resolution ESI mass spectrum (positive mode) of **2**.

Table S1. Crystallographic data for cobalt(III) complexes **1** and **2**.

**Metal complex**	**1**	**2**
CCDC No.	2521599	2521598
formula	$C_{41}CoF_8H_{40}N_4O_3P$ • $0.5C_4H_{10}O$	$C_{28}CoF_6H_{33}N_4ClP$
Fw	915.73	664.93
Crystal system	monoclinic	triclinic
Space group	$P2_1/c$	P-1
a, Å	11.5832(5)	8.0844(4)
b, Å	12.4800(6)	9.8894(5)
c, Å	29.0623(12)	18.2249(10)
α, deg.	90	99.447(3)
β, deg.	99.966(2)	93.247(2)
γ, deg.	90	103.161(2)
V, Å^3	4137.8(3)	1392.84(13)
Z	4	2
D_{calcd}, Mg/m^3	1.470	1.585
2θ / deg.	7.728 to 144.626	9.34 to 144.806
Reflections collected	75196	21145
Independent reflections	8143	5378
Goodness-of-fit on F^2	1.131	1.034
R_1, wR_2 [$I \geq 2\sigma(I)$]	0.0616, 0.1423	0.0631, 0.1539
R_1, wR_2 [all data]	0.0635, 0.1432	0.0699, 0.1606

Table S2. Selected bond lengths (Å) and angles (°) for cobalt(III) complex **1**.

Co(1)-O(1)	1.973(2)	Co(1)-N(3A)	1.986(10)
Co(1)-N(1A)	1.963(7)	Co(1)-N(3)	1.958(6)
Co(1)-N(1)	1.997(8)	Co(1)-N(4A)	1.982(7)
Co(1)-N(2A)	1.980(8)	Co(1)-N(4)	1.971(6)
Co(1)-N(2)	1.984(7)	Co(1)-C(24)	1.873(3)
O(1)-Co(1)-N(1)	83.3(3)	N(3)-Co(1)-N(1)	179.6(3)
O(1)-Co(1)-N(2A)	88.1(3)	N(3)-Co(1)-N(2)	87.3(2)
O(1)-Co(1)-N(2)	89.3(2)	N(3)-Co(1)-N(4)	93.8(2)
O(1)-Co(1)-N(3A)	94.0(4)	N(4)-Co(1)-O(1)	89.18(19)
O(1)-Co(1)-N(4A)	90.0(2)	N(4)-Co(1)-N(1)	86.3(3)
N(1A)-Co(1)-O(1)	85.1(3)	N(4)-Co(1)-N(2)	178.2(3)
N(1A)-Co(1)-N(2A)	95.5(3)	N(4A)-Co(1)-N(3A)	90.8(4)
N(1A)-Co(1)-N(3A)	178.7(4)	C(24)-Co(1)-O(1)	171.58(13)
N(1A)-Co(1)-N(4A)	88.2(3)	C(24)-Co(1)-N(1A)	87.3(3)
N(2A)-Co(1)-N(3A)	85.4(4)	C(24)-Co(1)-N(1)	88.5(3)
N(2A)-Co(1)-N(4A)	175.6(3)	C(24)-Co(1)-N(2A)	88.9(3)
N(2)-Co(1)-N(1)	92.6(3)	C(24)-Co(1)-N(2)	89.0(3)
N(3)-Co(1)-O(1)	96.4(2)	C(24)-Co(1)-N(3A)	93.6(4)

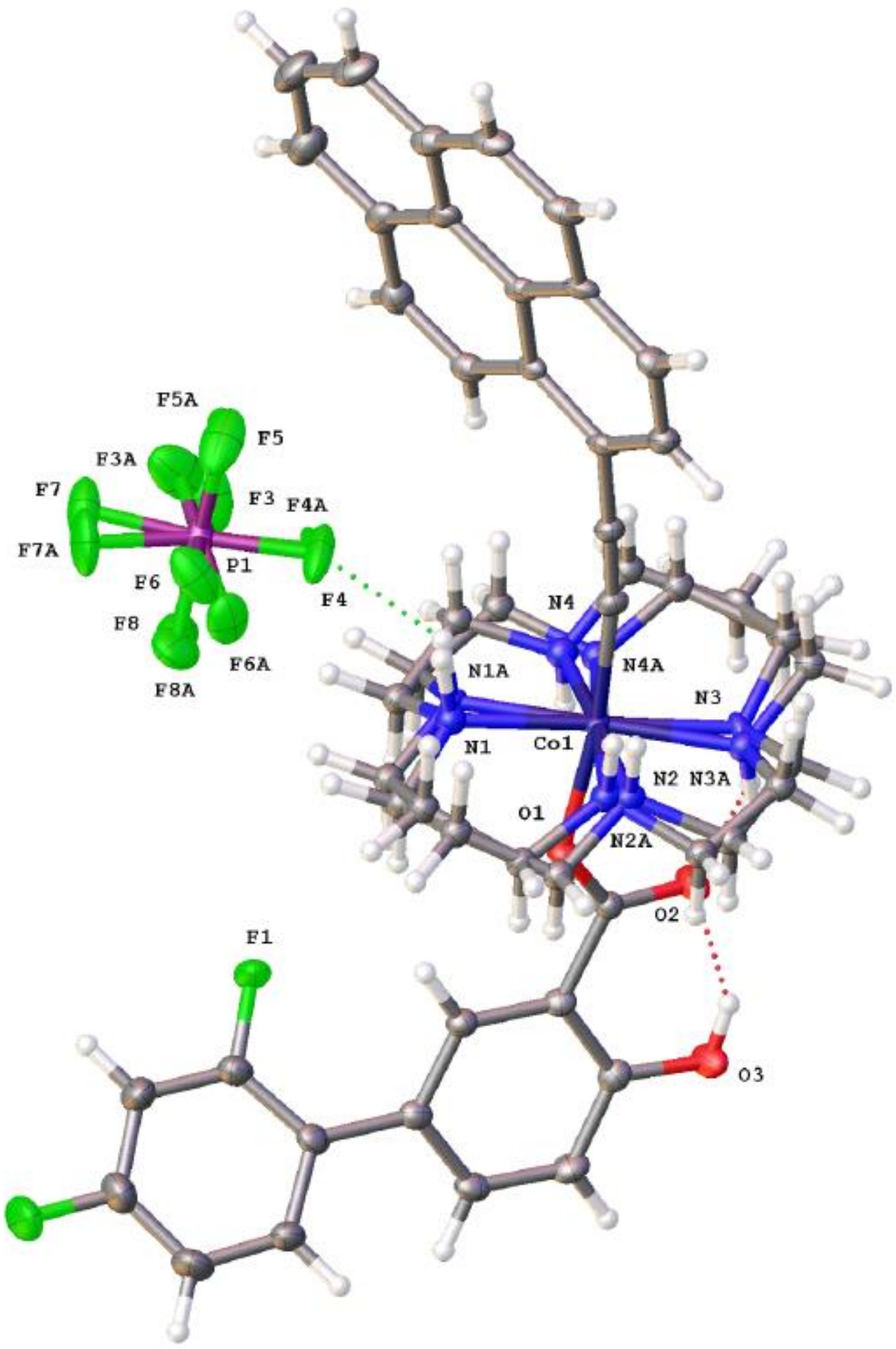
F5A
F5
F3A
F7
F3
F4A
F7A
P1
F6
F4
F8
F6A
F8A
N4
N1A
N4A
N3
N1
Co1
N2
N3A
O1
N2A
O2
F1
O3
F2

Table S3. Selected bond lengths (Å) and angles (°) for cobalt(III) complex **2**.

Co(1)-Cl(1)	2.3205(11)	Co(1)-N(3)	1.968(3)
Co(1)-N(1)	1.985(3)	Co(1)-N(4)	1.970(3)
Co(1)-N(2)	1.979(3)	Co(1)-C(11)	1.879(4)
Cl(1)-Co(1)-N(1)	91.16(10)	N(2)-Co(1)-N(1)	92.90(14)
Cl(1)-Co(1)-N(2)	92.82(10)	N(3)-Co(1)-N(1)	179.23(14)
Cl(1)-Co(1)-N(3)	89.15(10)	N(3)-Co(1)-N(2)	86.38(14)
Cl(1)-Co(1)-N(4)	87.72(10)	N(3)-Co(1)-N(4)	94.42(14)
C(11)-Co(1)-N(1)	87.37(15)	N(4)-Co(1)-N(1)	86.30(14)
C(11)-Co(1)-N(2)	88.81(16)	N(4)-Co(1)-N(2)	179.05(14)
C(11)-Co(1)-N(3)	92.34(15)	C(11)-Co(1)-Cl(1)	177.86(13)
C(11)-Co(1)-N(4)	90.63(16)		

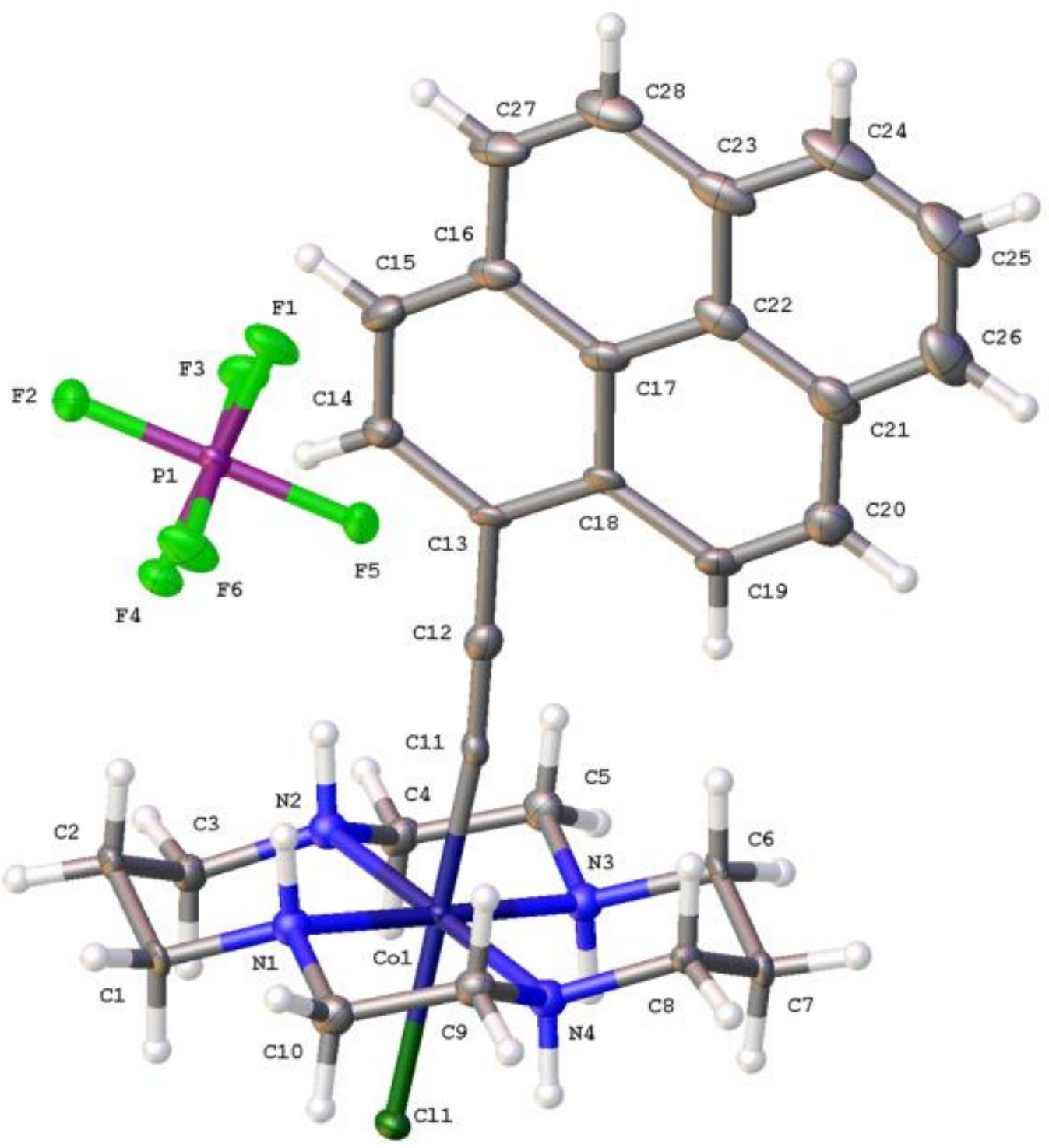

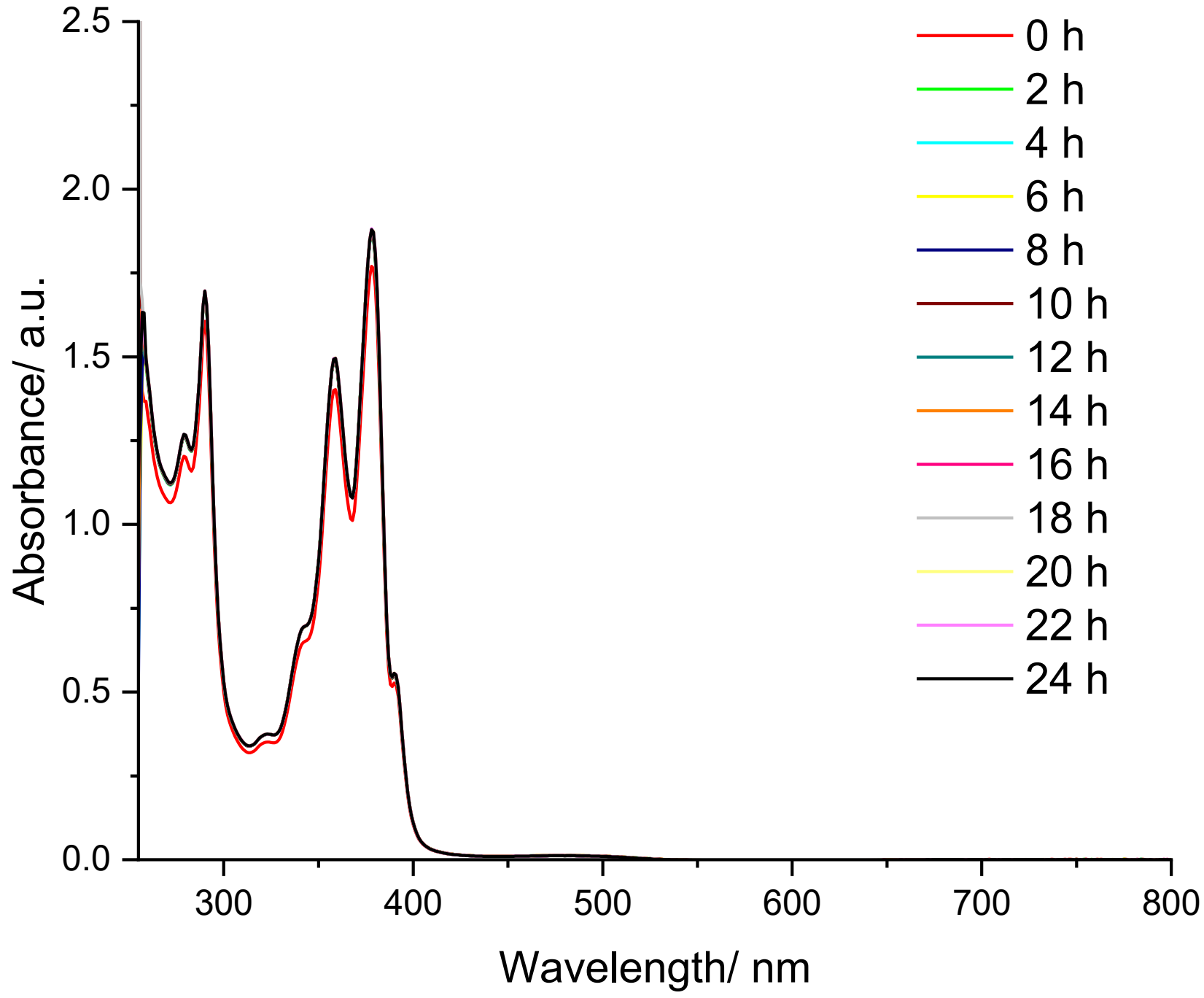

Figure S14. UV-Vis spectra of **1** (50 µM) in DMSO over the course of 24 h at 37 ºC.

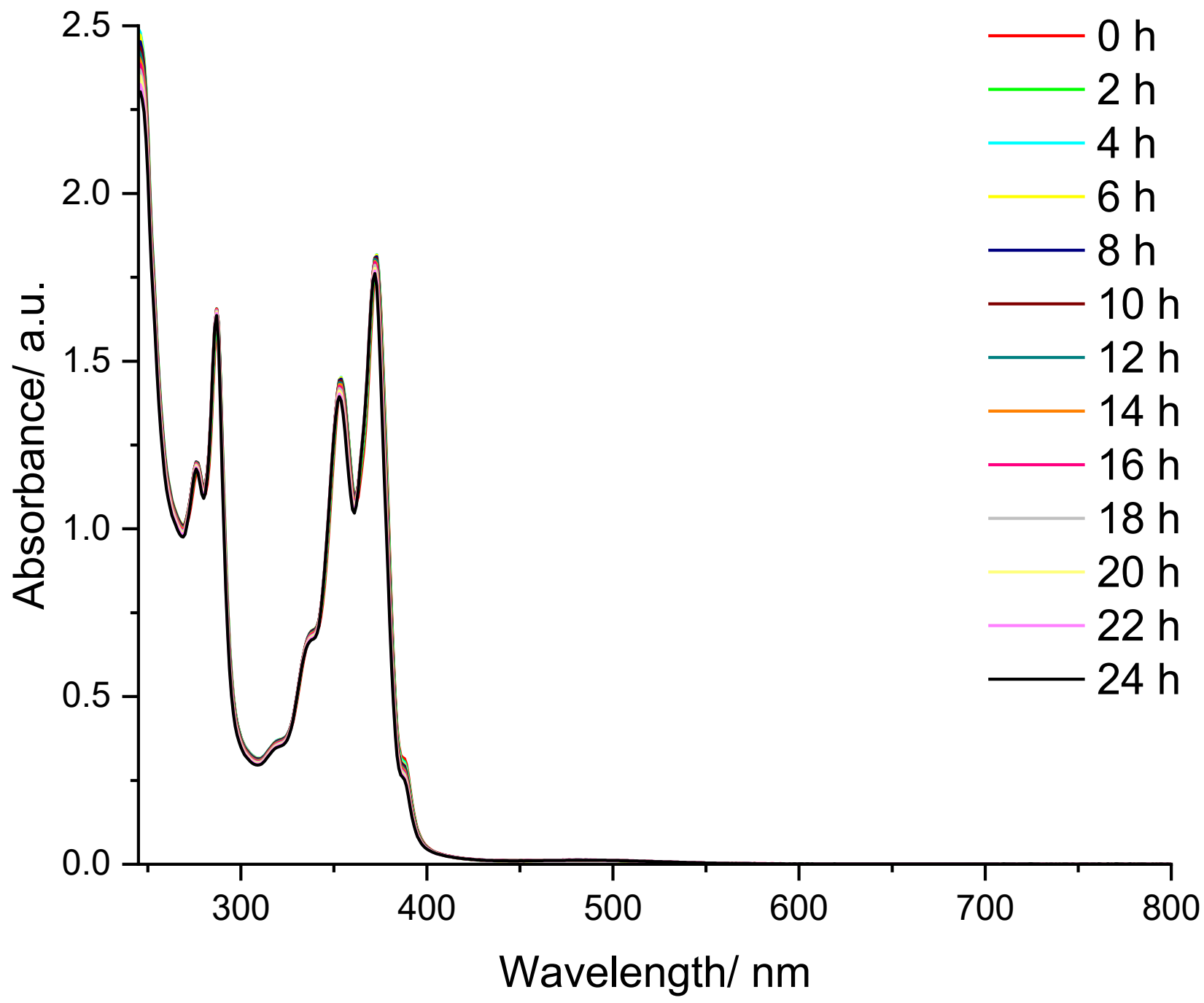

Figure S15. UV-Vis spectra of **1** (50 µM) in H_2O:DMSO (8:2) over the course of 24 h at 37 ºC.

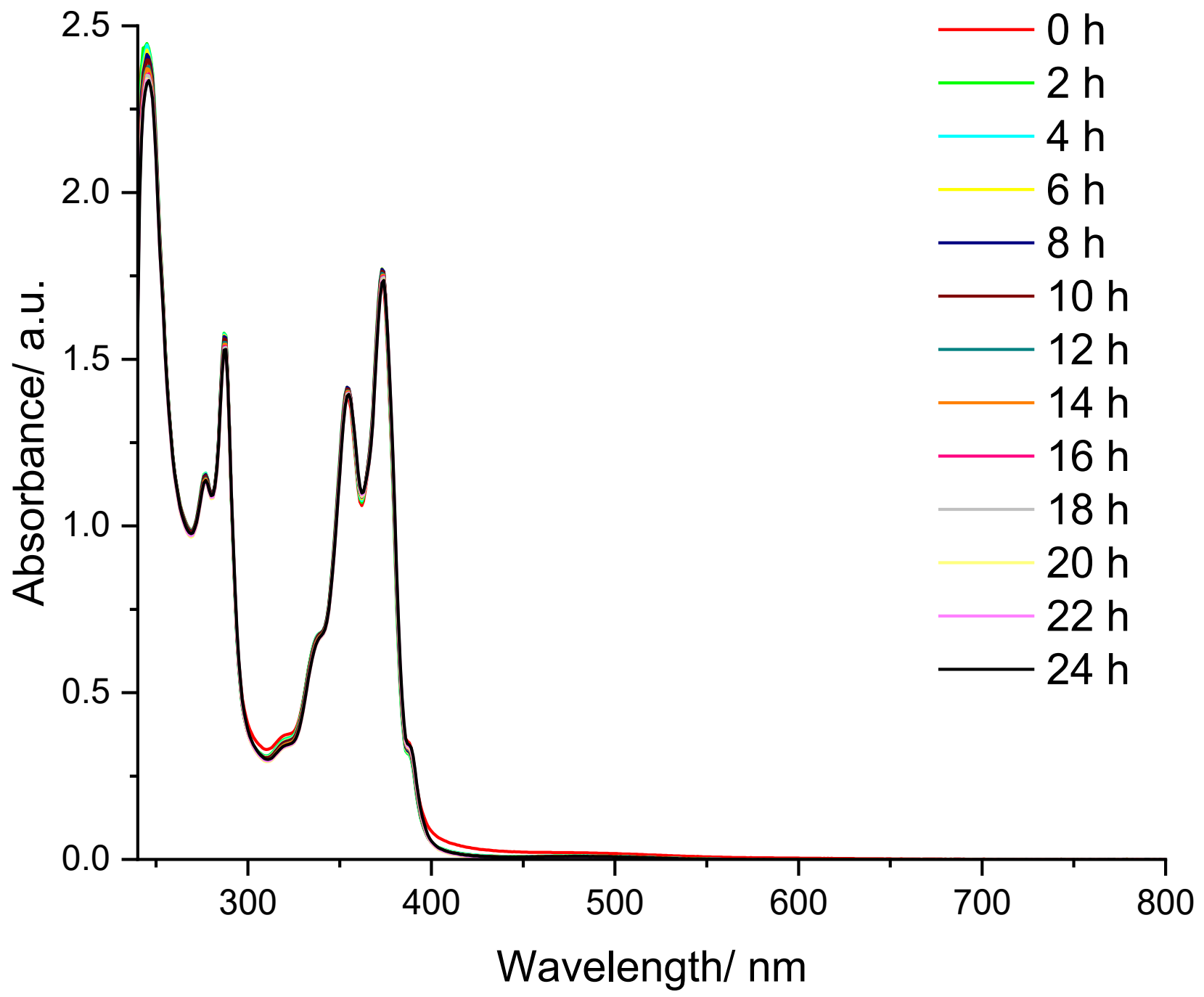

Figure S16. UV-Vis spectra of **1** (50 µM) in PBS:DMSO (8:2) over the course of 24 h at 37 ºC.

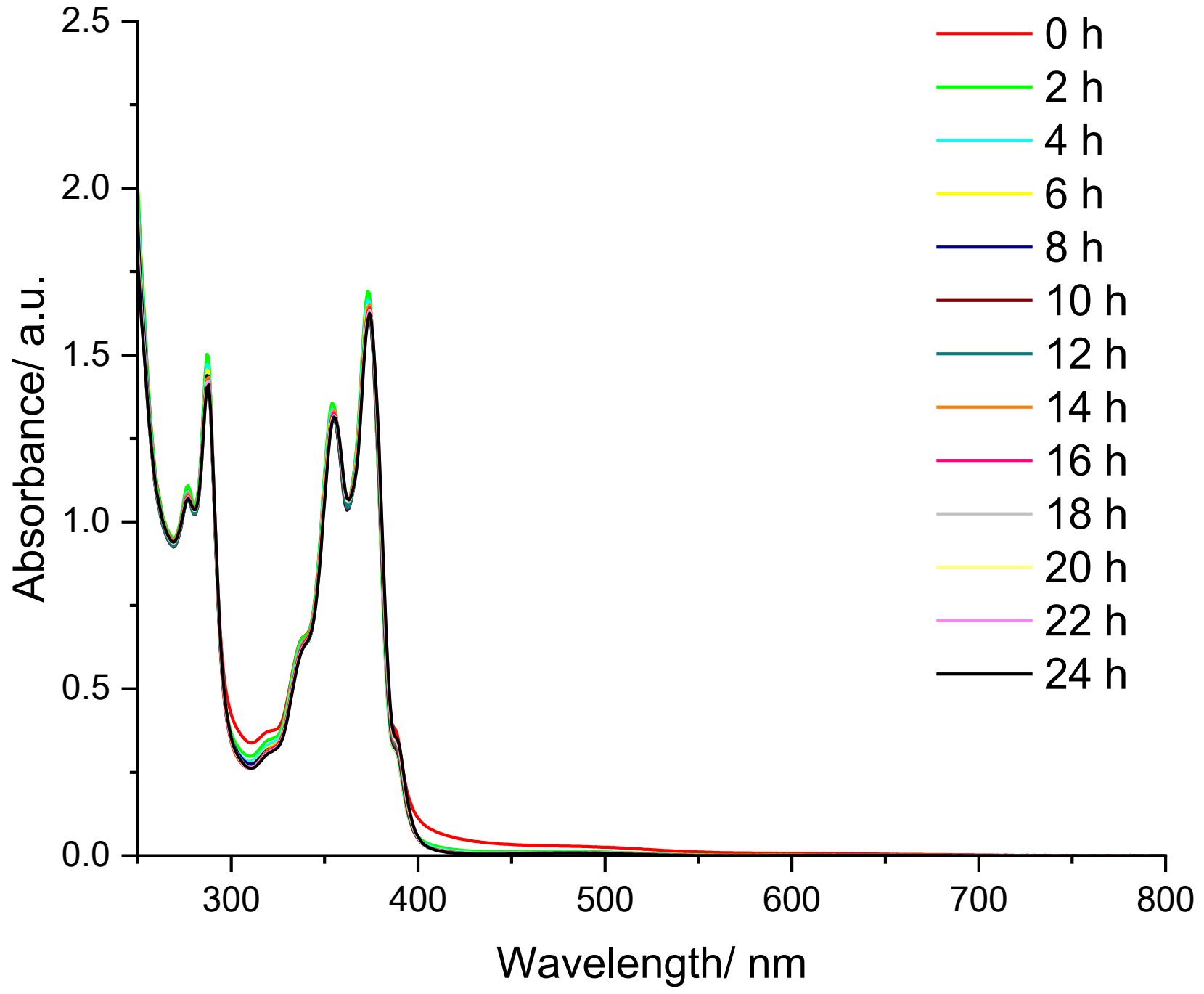

Figure S17. UV-Vis spectra of **1** (50 µM) in Mammary Epithelial Cell Growth Medium (MEGM):DMSO (8:2) over the course of 24 h at 37 ºC.

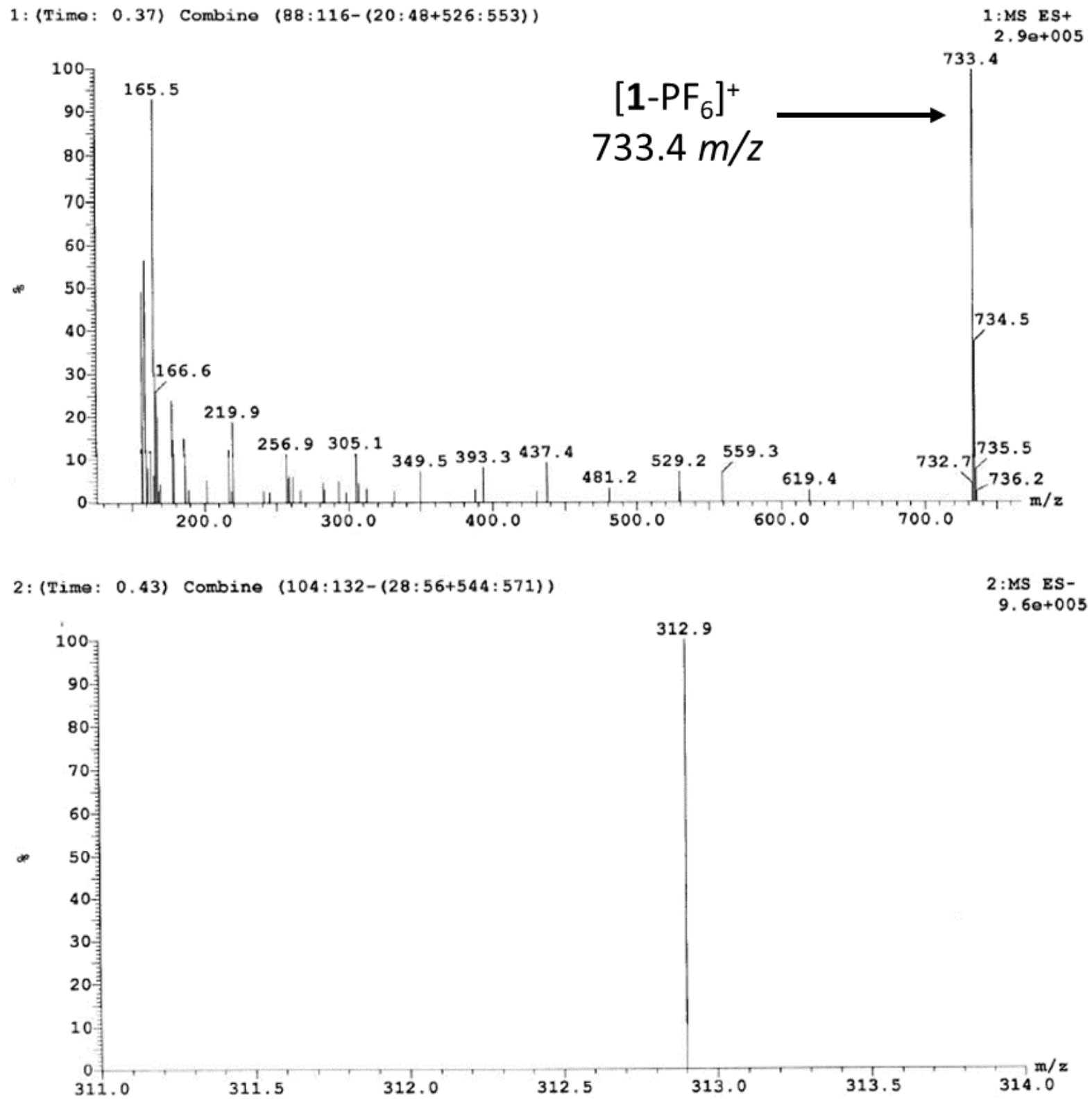

Figure S18. ESI mass spectrum (positive and negative modes) of **1** (50 μM) in H_2O:DMSO (8:2) after 24 h incubation.

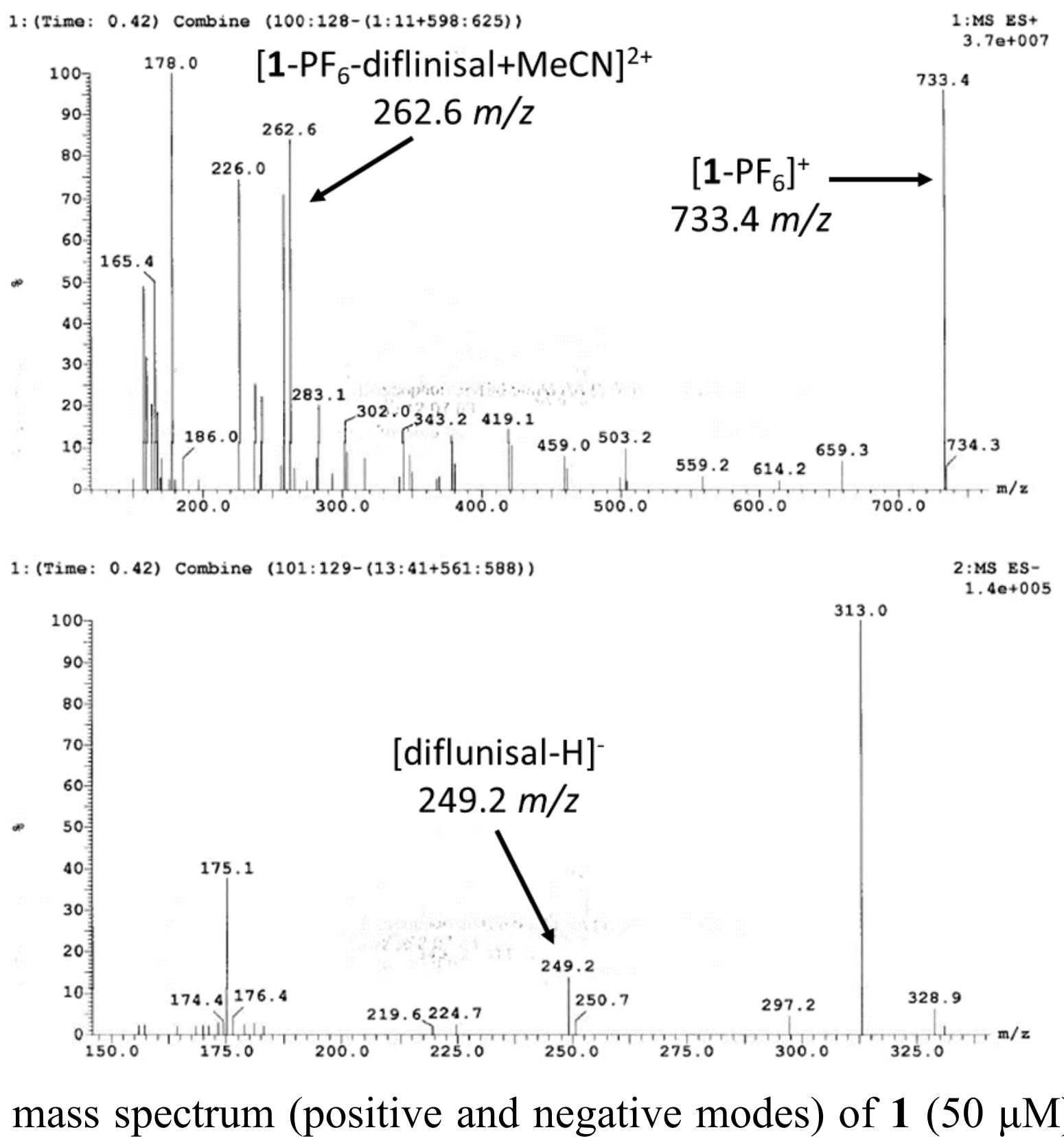

Figure S19. ESI mass spectrum (positive and negative modes) of **1** (50 μM) in H_2O:DMSO (8:2), in the presence of ascorbic acid (250 μM) after 24 h incubation.

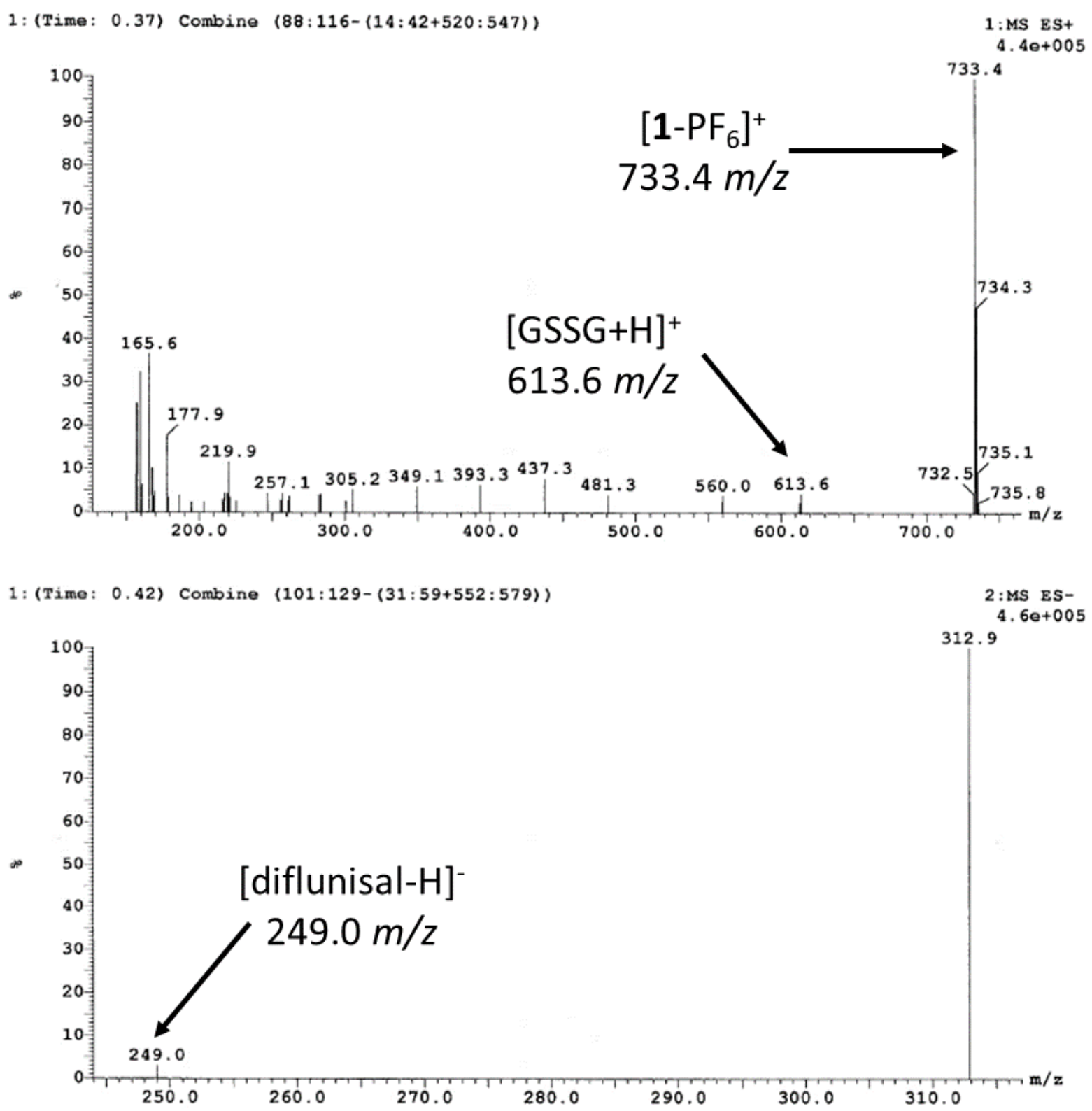

Figure S20. ESI mass spectrum (positive and negative modes) of **1** (50 µM) in H_2O:DMSO (8:2), in the presence of glutathione (500 µM) after 24 h incubation.

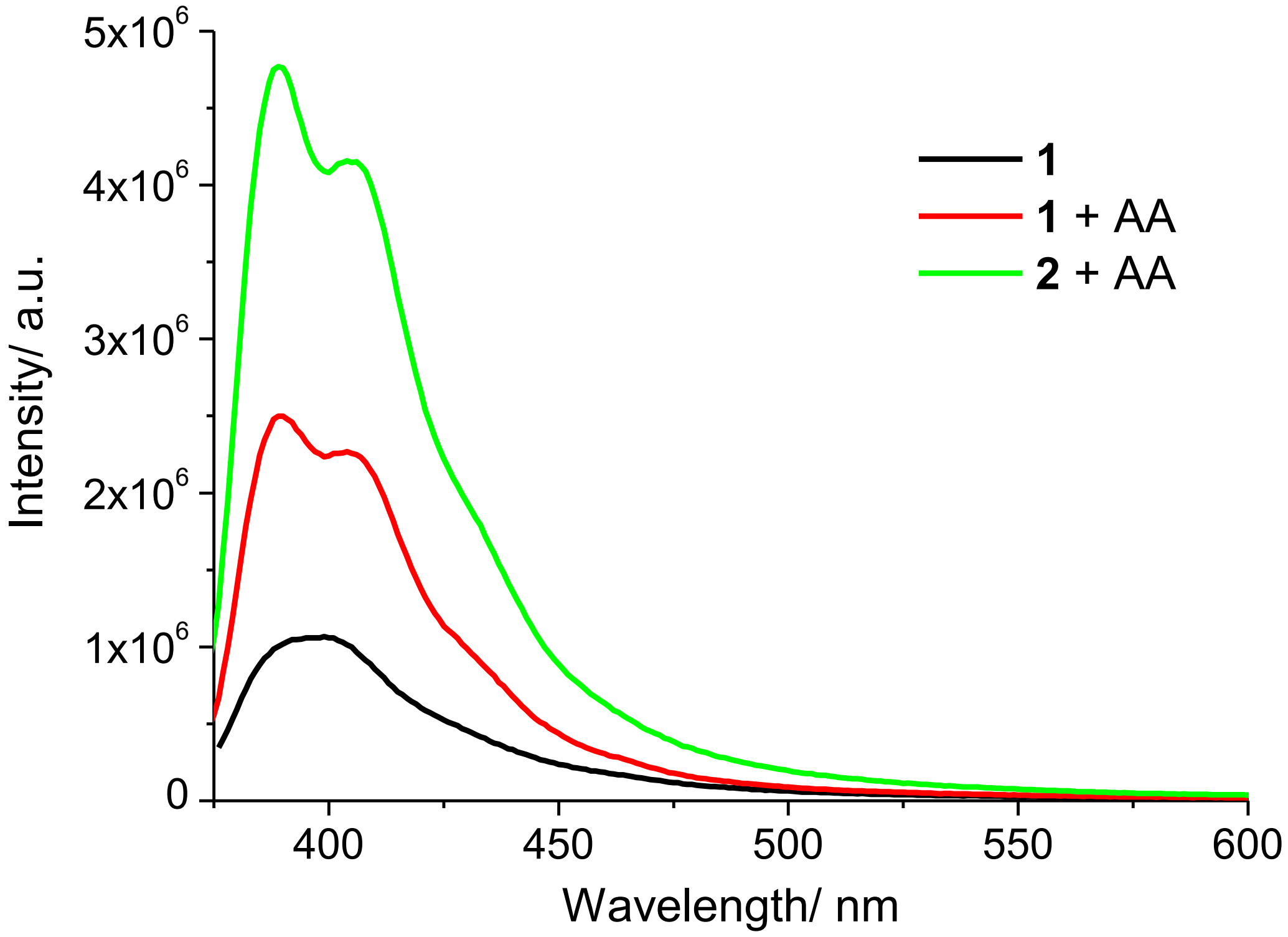

Figure S21. Emission spectra in acetonitrile (λ_{ex} =356 nm) of **1** (25 µM) and **1** (25 µM) or **2** (25 µM) in the presence of ascorbic acid (250 µM).

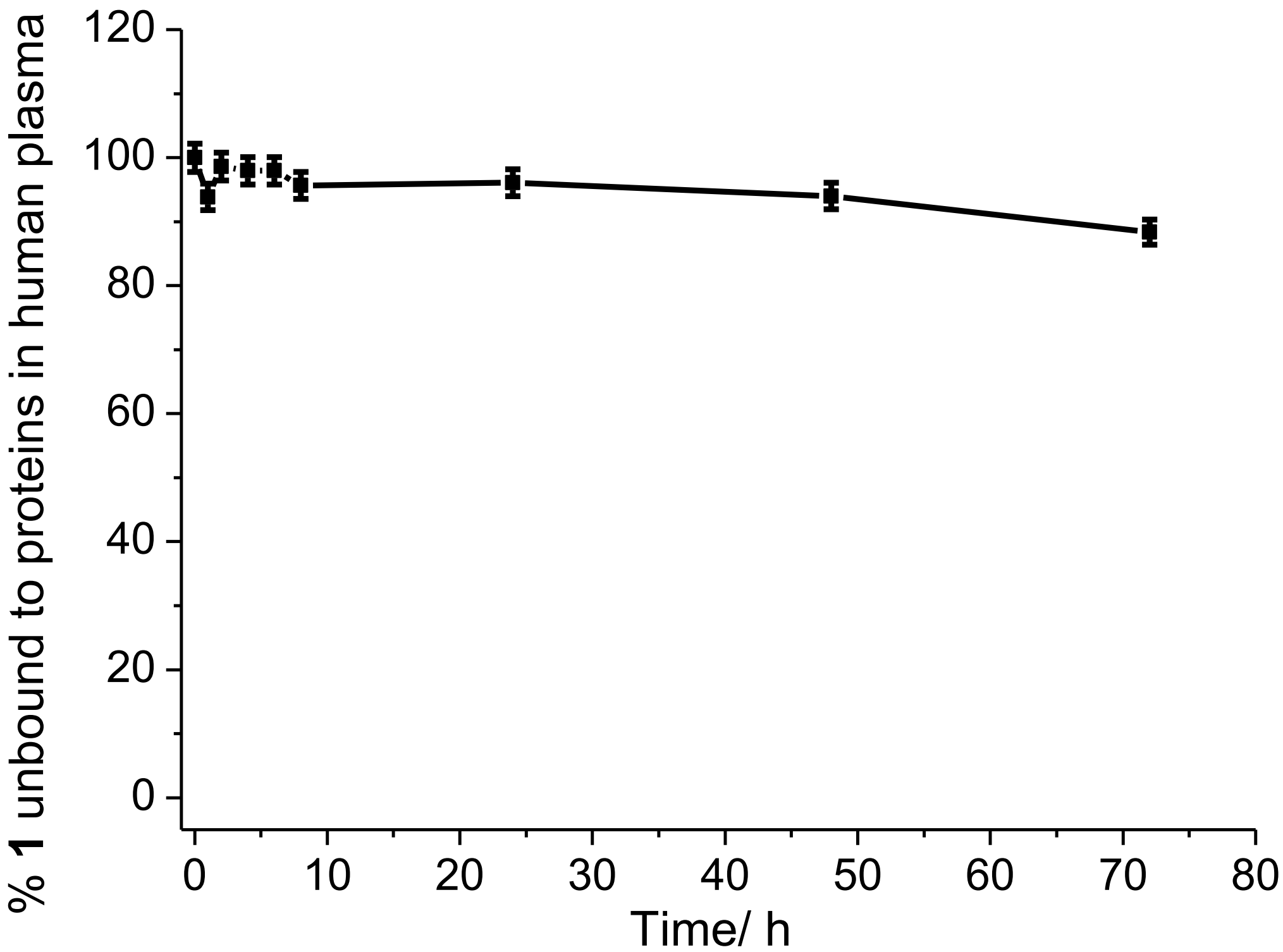

Figure S22. The stability profile of **1** (1 mM) in human plasma over the course of 72 h at 37 °C.

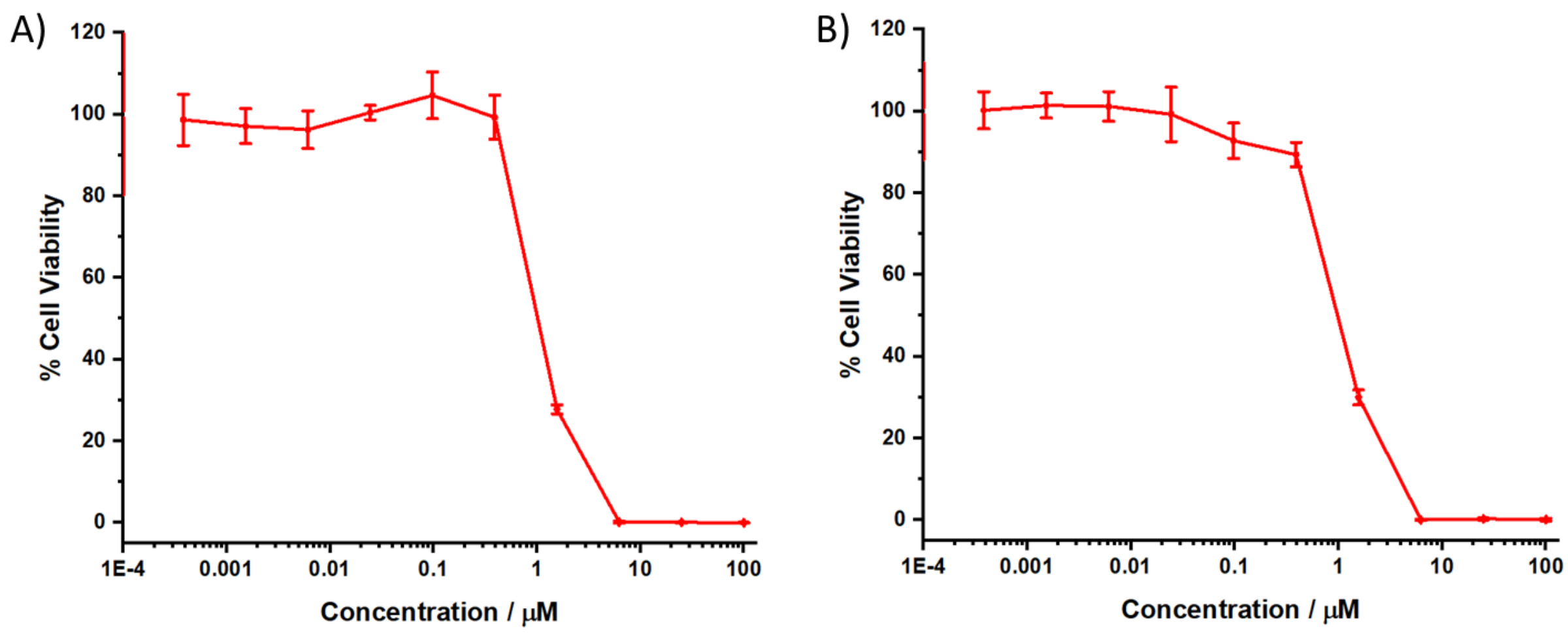

Figure S23. Representative dose-response curves for the treatment of (A) HMLER and (B) HMLER-shEcad cells with **1** after 72 h incubation. Error bars represent standard deviations.

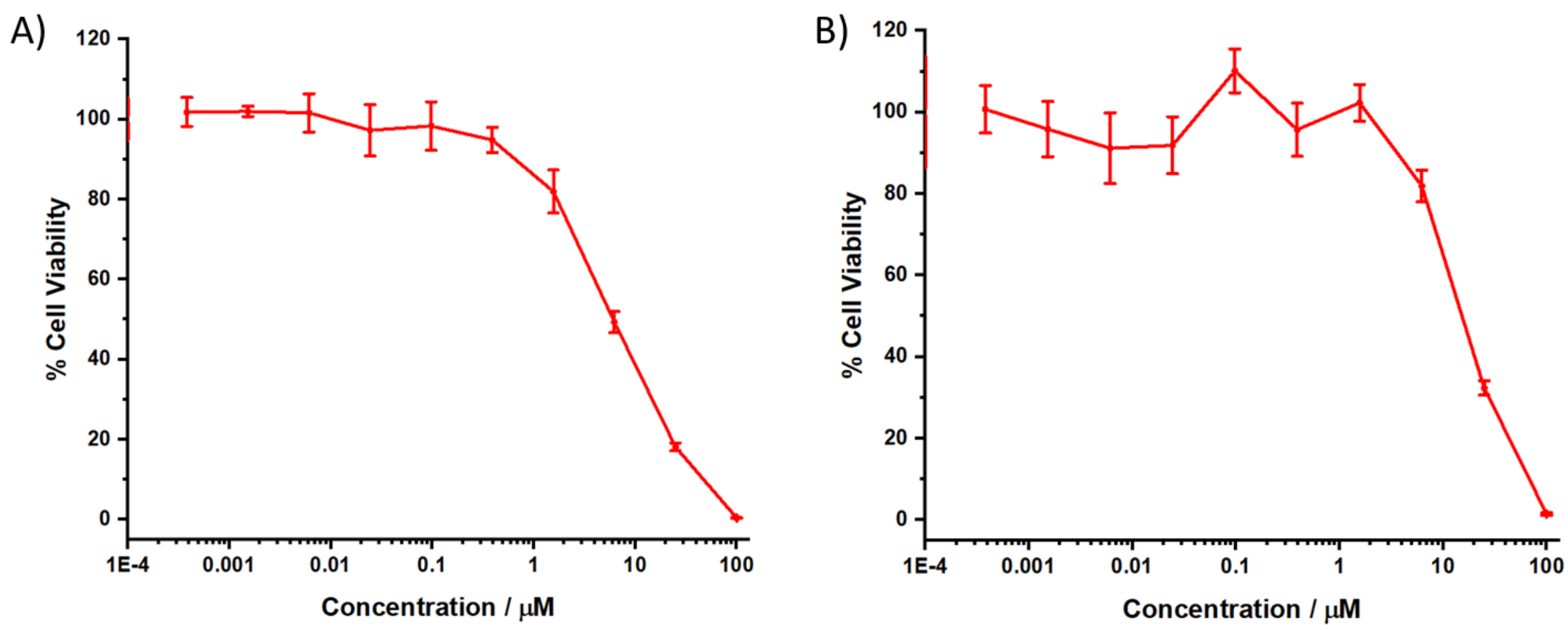

Figure S24. Representative dose-response curves for the treatment of (A) HMLER and (B) HMLER-shEcad cells with **2** after 72 h incubation. Error bars represent standard deviations.

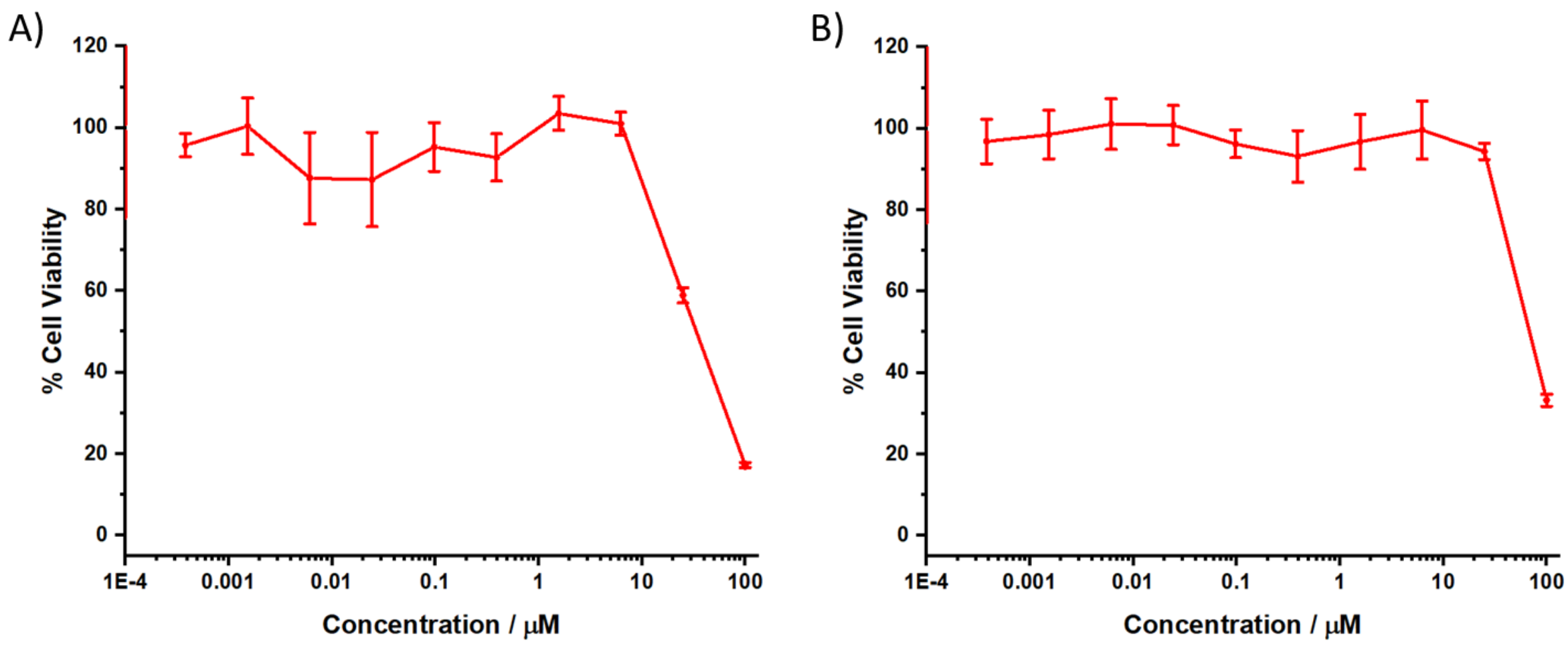

Figure S25. Representative dose-response curves for the treatment of (A) HMLER and (B) HMLER-shEcad cells with diflunisal after 72 h incubation. Error bars represent standard deviations.

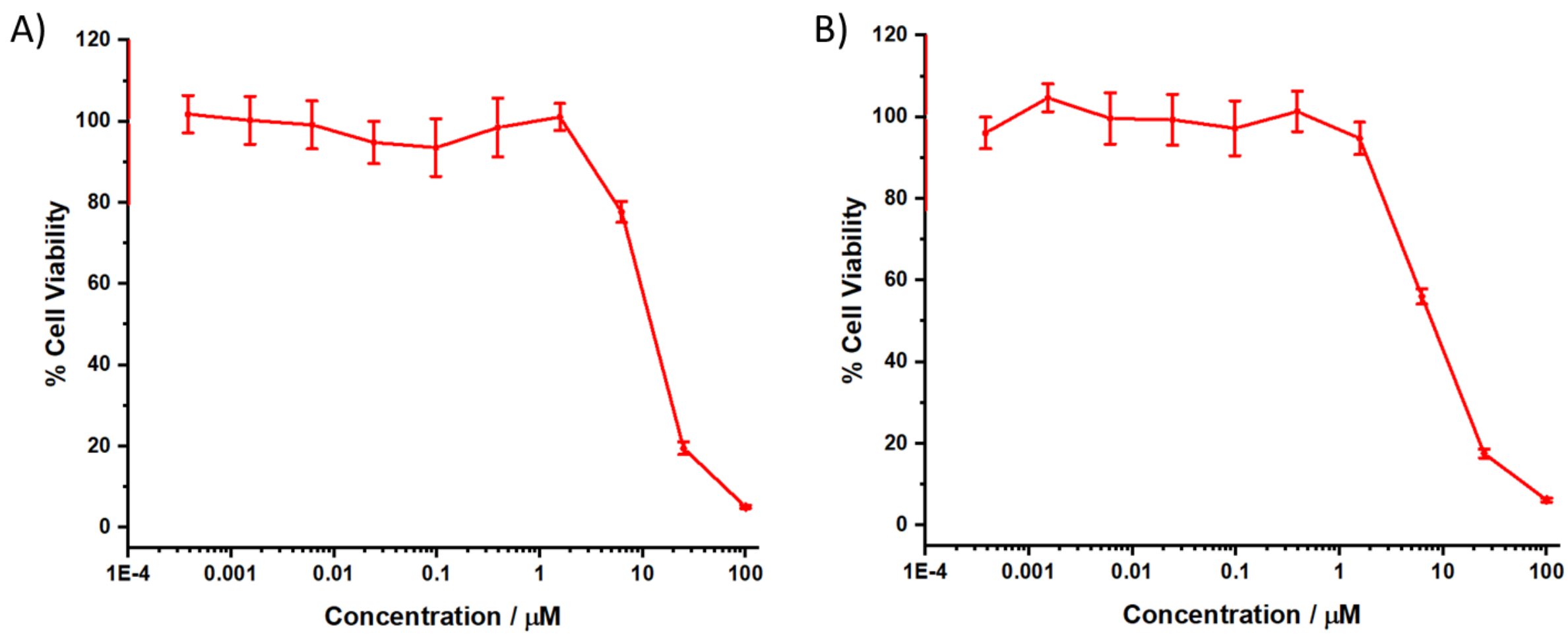

Figure S26. Representative dose-response curves for the treatment of (A) HMLER and (B) HMLER-shEcad cells with 1-ethynyl pyrene after 72 h incubation. Error bars represent standard deviations.

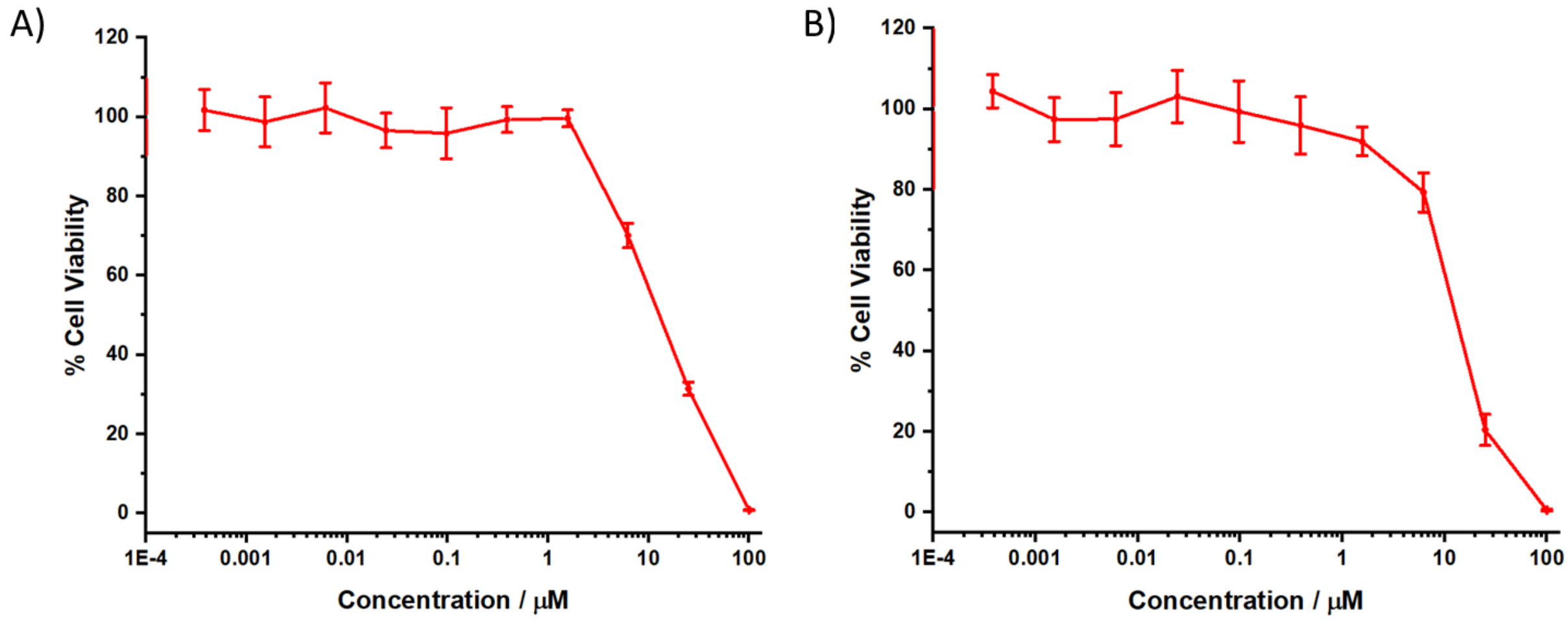

Figure S27. Representative dose-response curves for the treatment of HMLER-shEcad cells with (A) 1:1 mixture of **2** and diflunisal and (B) 1:1:1 mixture of **3**, 1-ethynyl pyrene, and diflunisal after 72 h incubation. Error bars represent standard deviations.

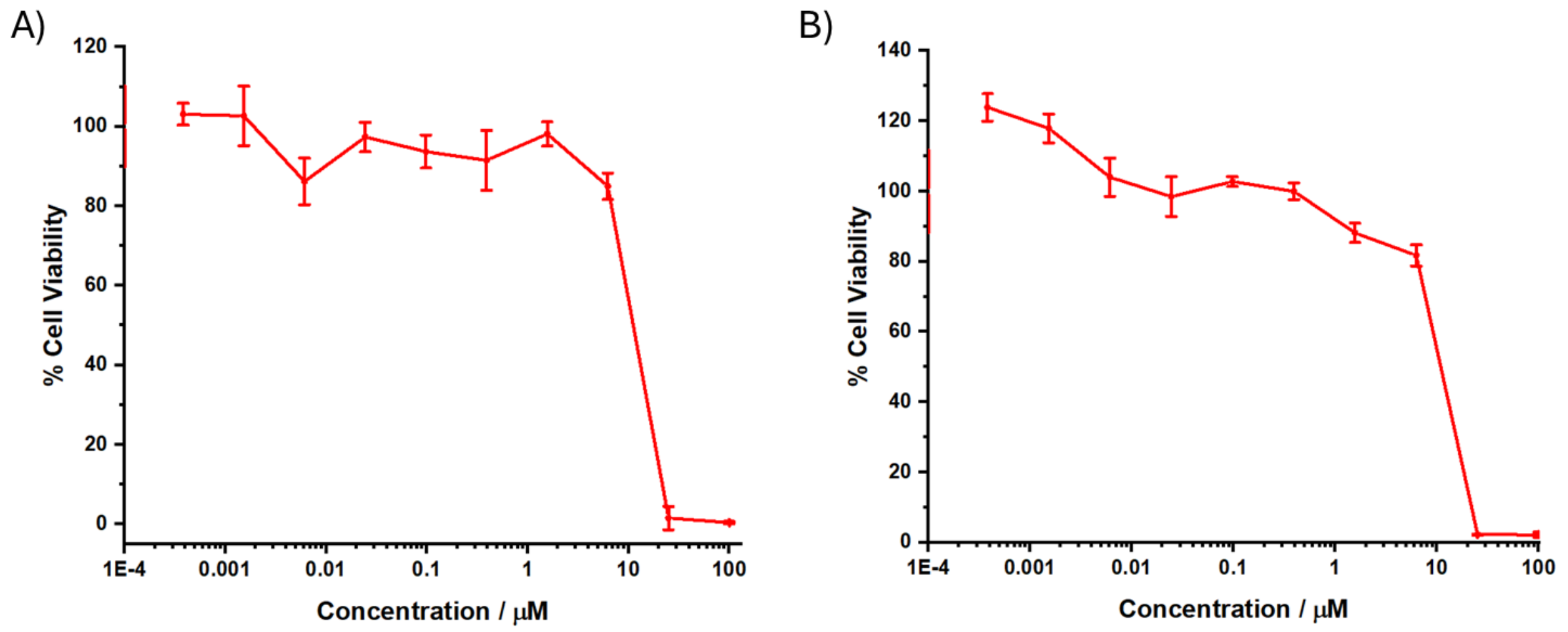

Figure S28. Representative dose-response curves for the treatment of (A) BEAS-2B cells or (B) HEK 293 cells with **1** after 72 h incubation. Error bars represent standard deviations.

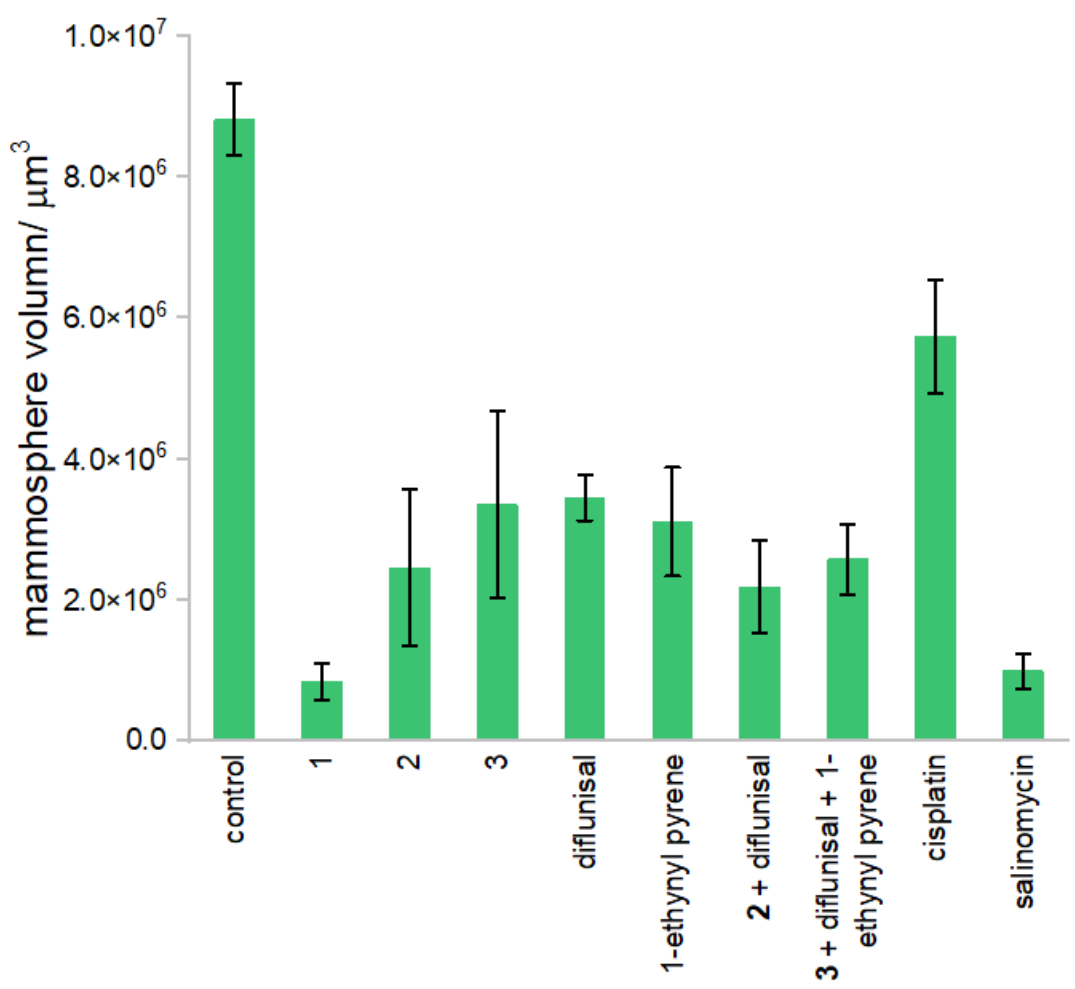

Figure S29. Average volume (μm^3) of mammospheres in the absence and presence of cobalt(III) complexes **1-3**, diflunisal, 1-ethynyl pyrene, **2** + diflunisal (1:1), **3** + diflunisal + 1-ethynyl pyrene (1:1:1), cisplatin or salinomycin (IC_{20} value for 5 days).

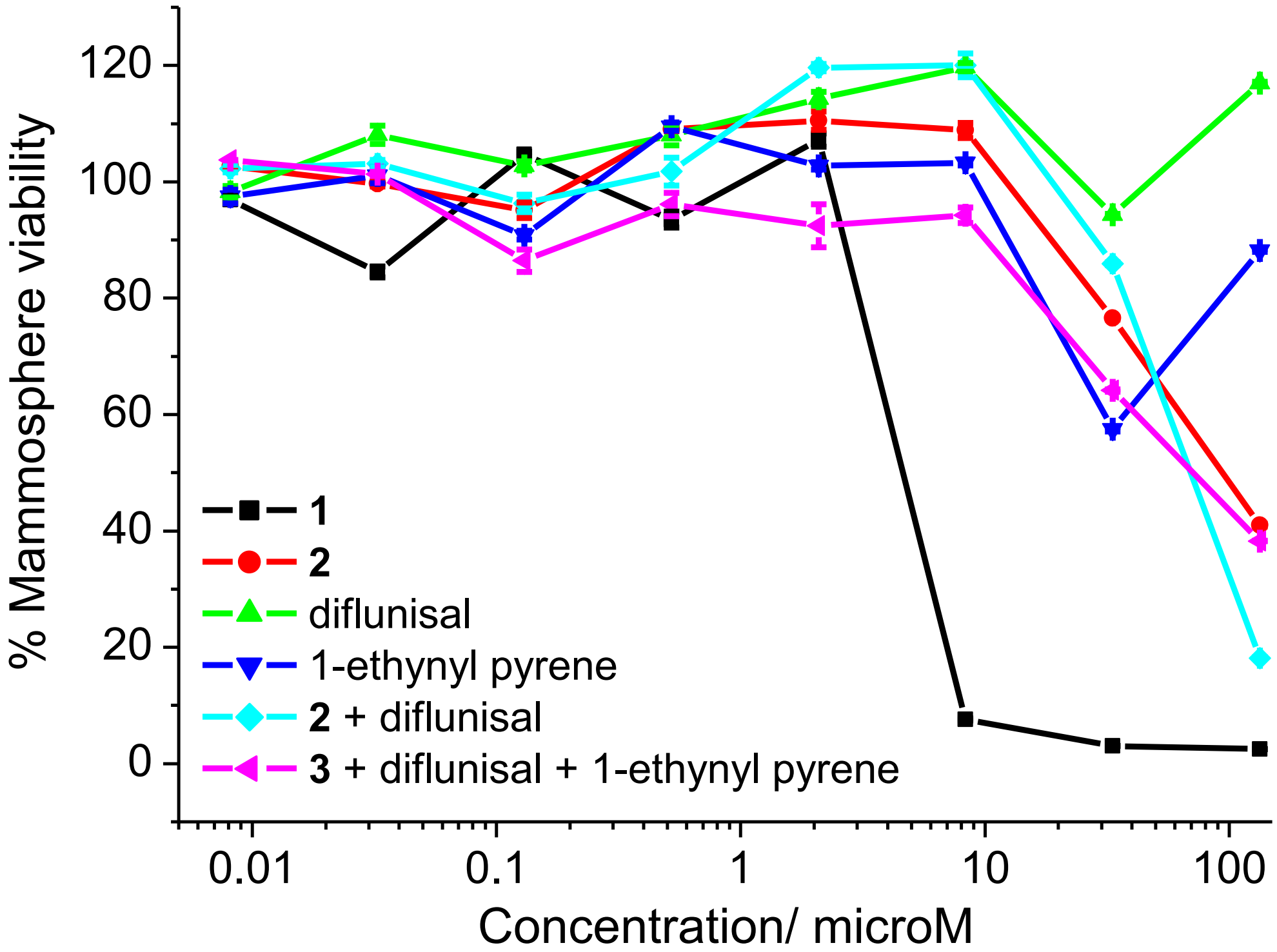

Figure S30. Representative dose-response curves for the treatment of HMLER-shEcad mammospheres with cobalt(III) complexes **1** and **2**, diflunisal, 1-ethynyl pyrene, **2** + diflunisal (1:1), or **3** + diflunisal + 1-ethynyl pyrene (1:1:1).

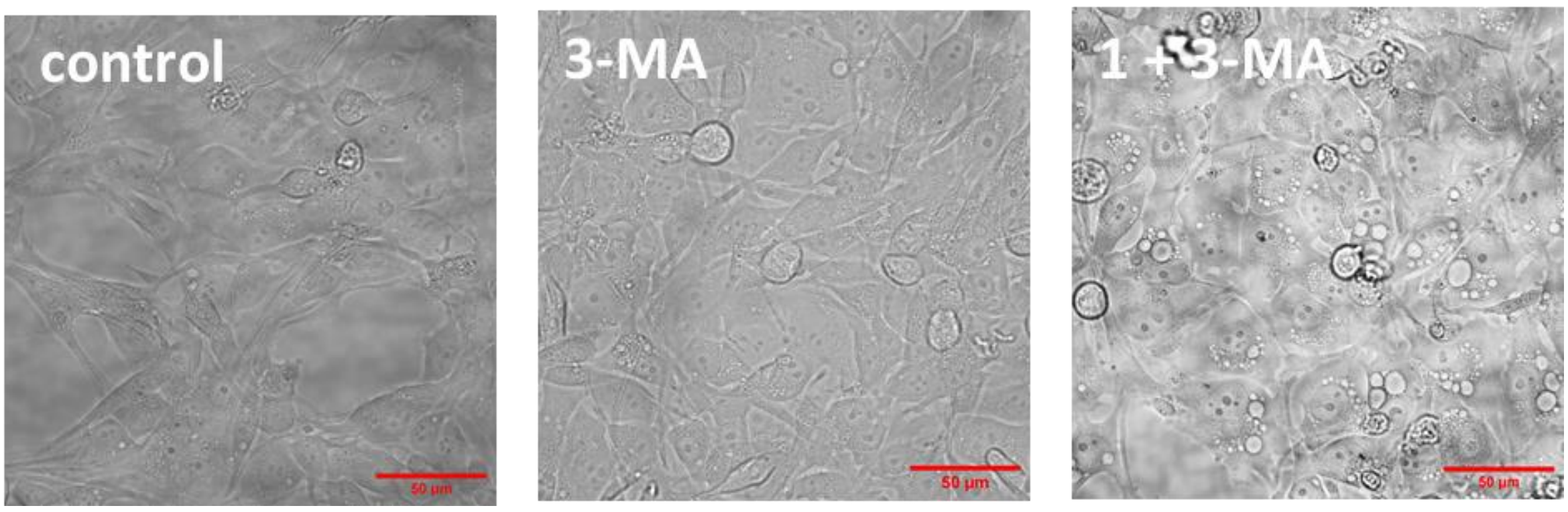

Figure S31. Representative bright-field images (× 60) of untreated HMLER-shEcad cells, HMLER-shEcad cells treated with 3-methyladenine only (1 mM for 48 h) and HMLER-shEcad cells co-treated with **1** (2 × IC_{50} value for 48 h) and 3-methyladenine (1 mM for 48 h).

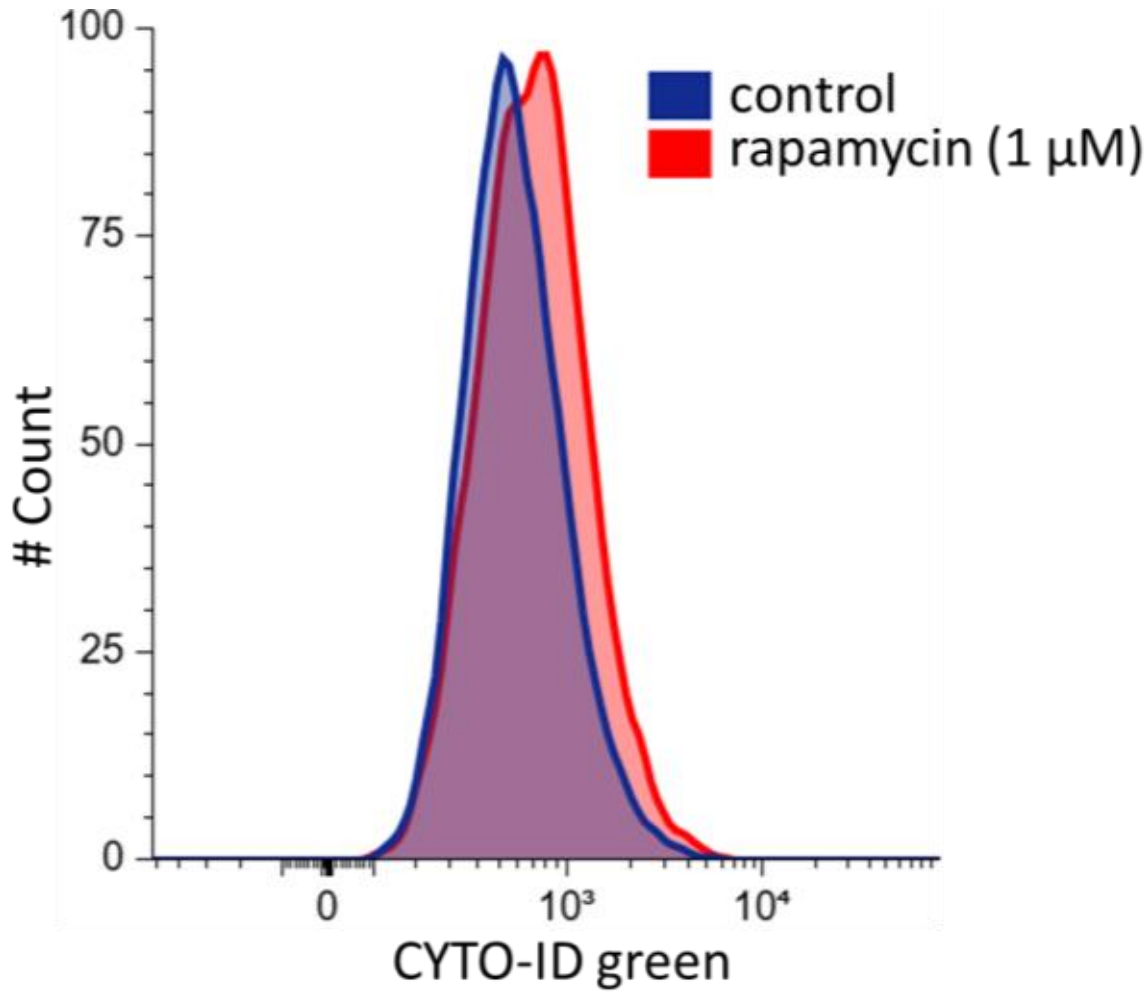

Figure S32. Representative histograms displaying the green fluorescence emitted by CYTO-ID green reagent-stained HMLER-shEcad cells untreated (blue) and treated with rapamycin (1 μM, red) for 24 h.

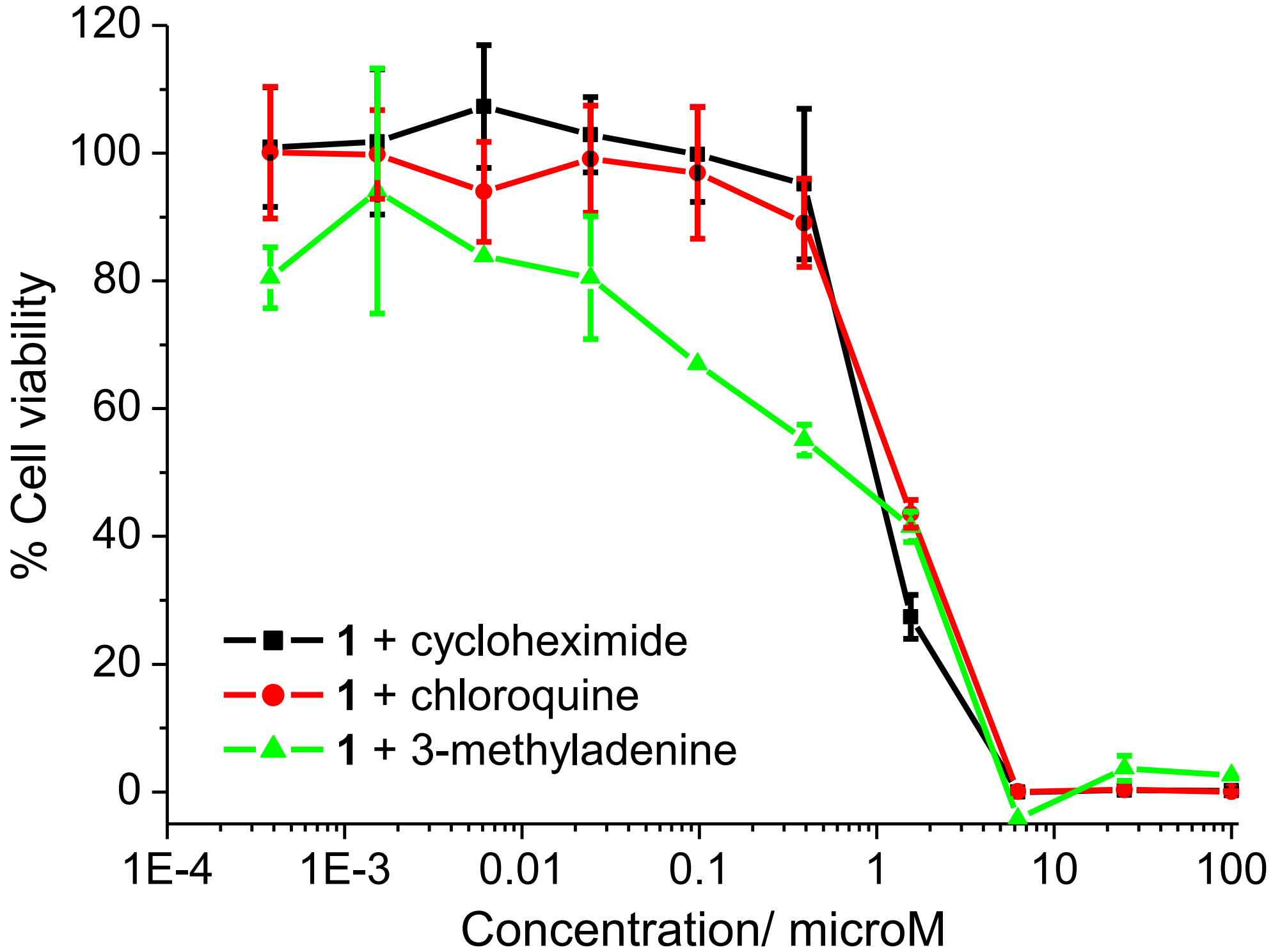

Figure S33. Representative dose-response curves for the treatment of HMLER-shEcad cells with **1** and cycloheximide (1 μM) or chloroquine (10 μM) or 3-methyladenine (1 mM) after 72 h incubation. Error bars represent standard deviations.

Table S4. IC_{50} values of **1** against HMLER-shEcad cells in the absence and presence of paraptosis (cycloheximide, 1 µM) or autophagy (chloroquine, 10 µM and 3-methyladenine, 1 mM) or methuosis (EIPA, pre-treated with 4 µM for 6 h) inhibitors. [a] Determined after 72 h incubation (mean of three independent experiments ± SD).

Treatment	HMLER-shEcad [µM][a]
**1**	0.98 ± 0.02
**1** + cycloheximide	0.97 ± 0.11
**1** + chloroquine	1.29 ± 0.08
**1** + 3-methyladenine	0.69 ± 0.05
**1** + EIPA	2.37 ± 0.01

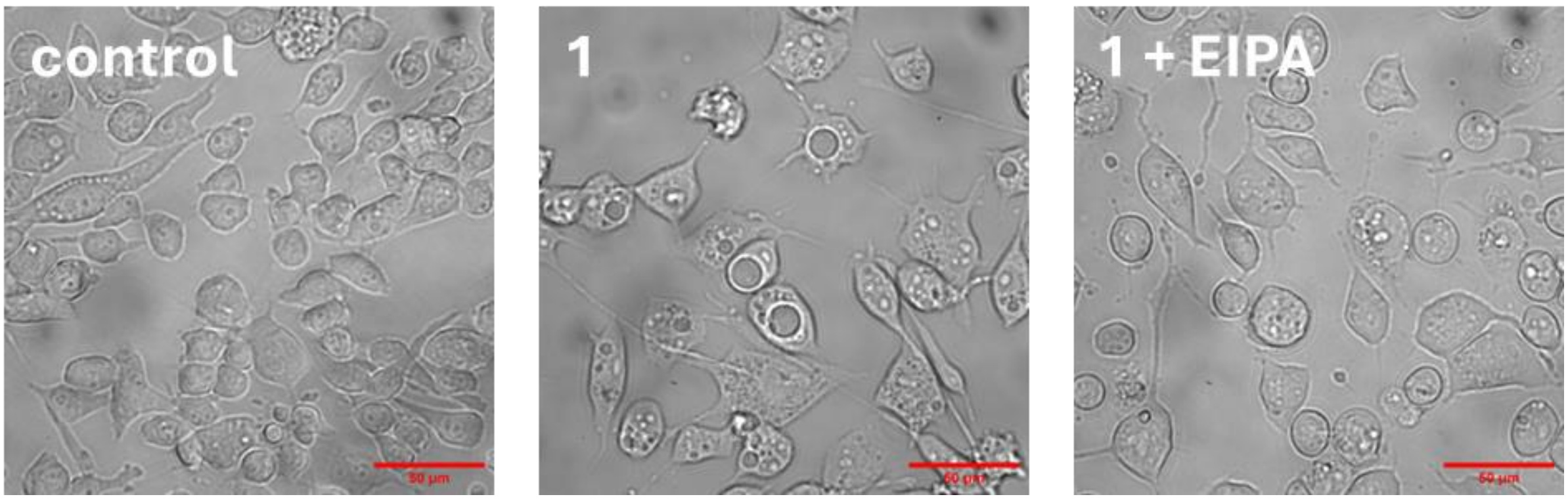

Figure S34. Representative bright-field images (× 60) of untreated HMLER-shEcad cells, HMLER-shEcad cells treated with **1** (2 × IC_{50} value for 48 h) and HMLER-shEcad cells co-treated with **1** (2 × IC_{50} value for 48 h) and EIPA (pre-treated with 4 µM for 6 h).

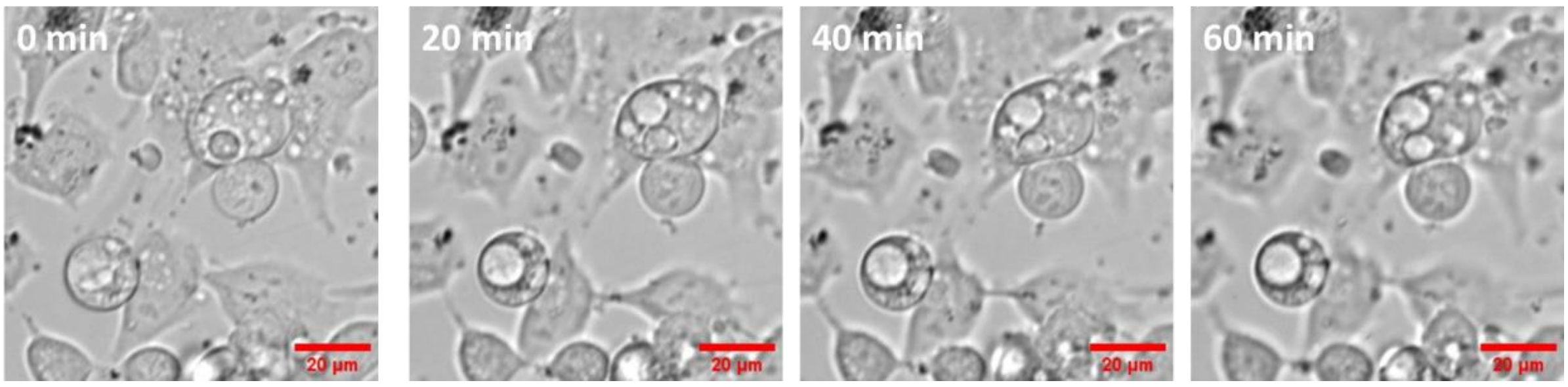

Figure S35. Representative live cell images (× 60) of HMLER-shEcad cells treated with **1** (4 × IC_{50} value) over a 60 min period.

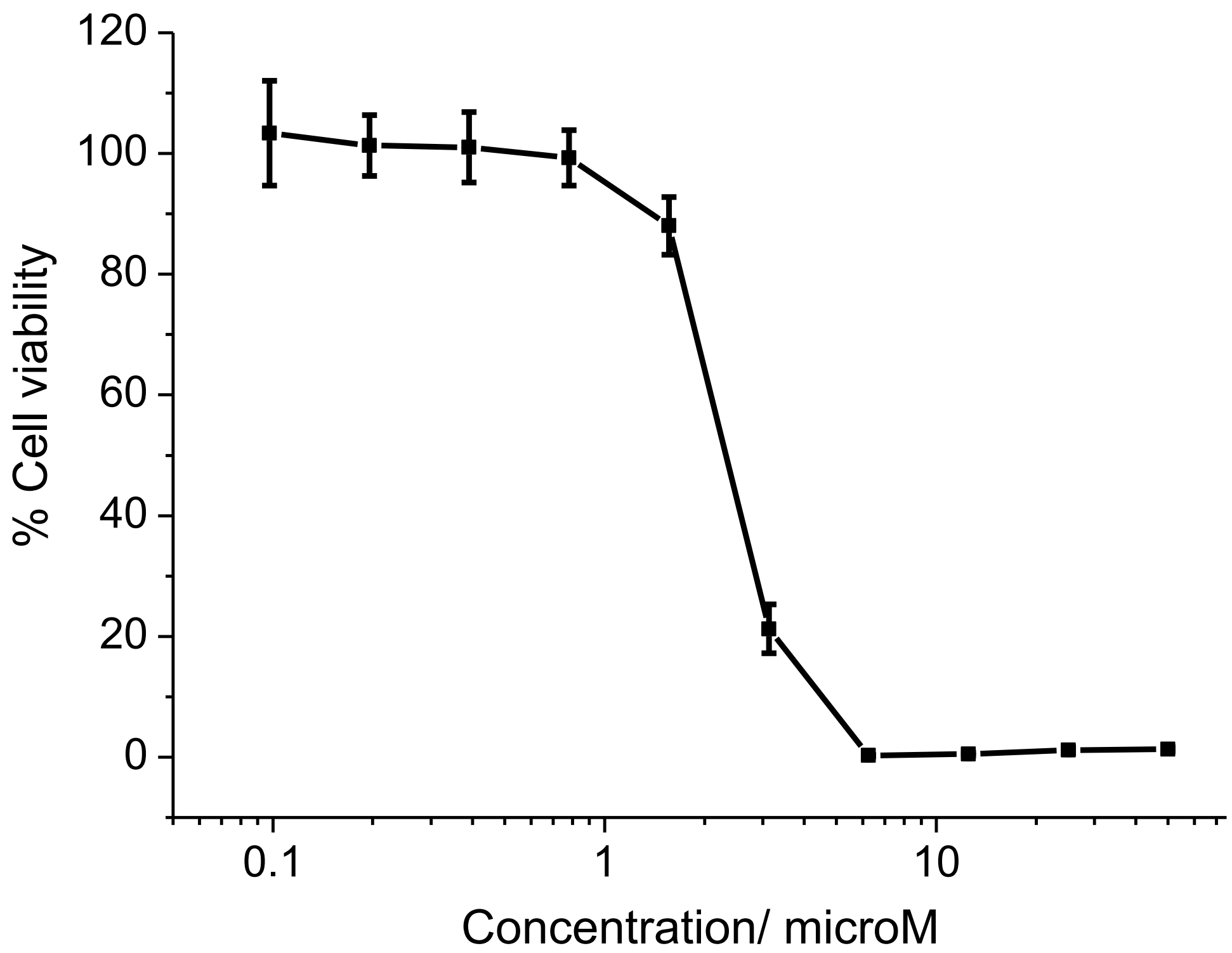

Figure S36. Representative dose-response curves for the treatment of HMLER-shEcad cells with **1** and EIPA (pre-treated with 4 μM for 6 h) after 72 h incubation. Error bars represent standard deviations.

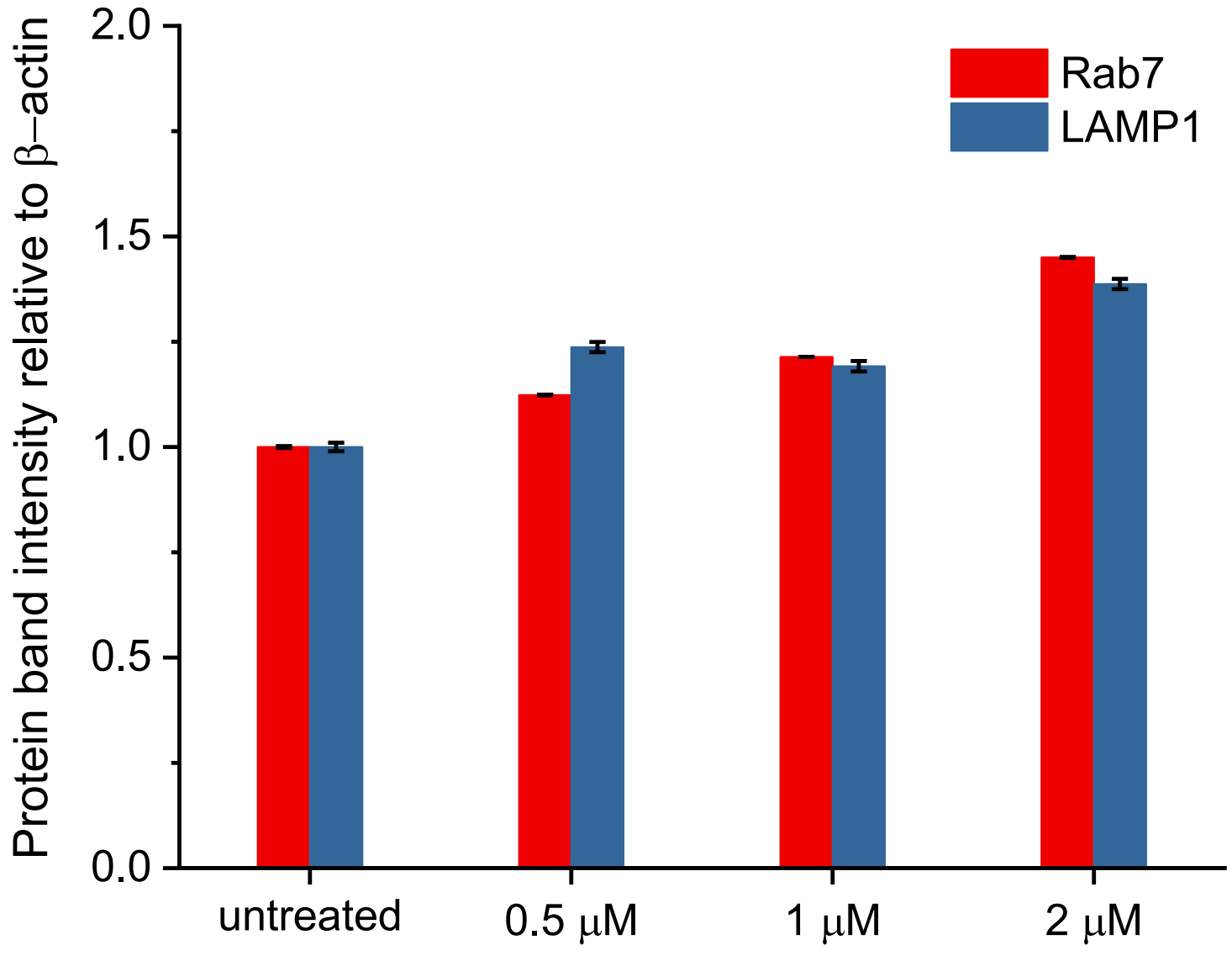

Figure S37. Protein band intensities corresponding to the immunoblotting analysis shown in Figure 4A.

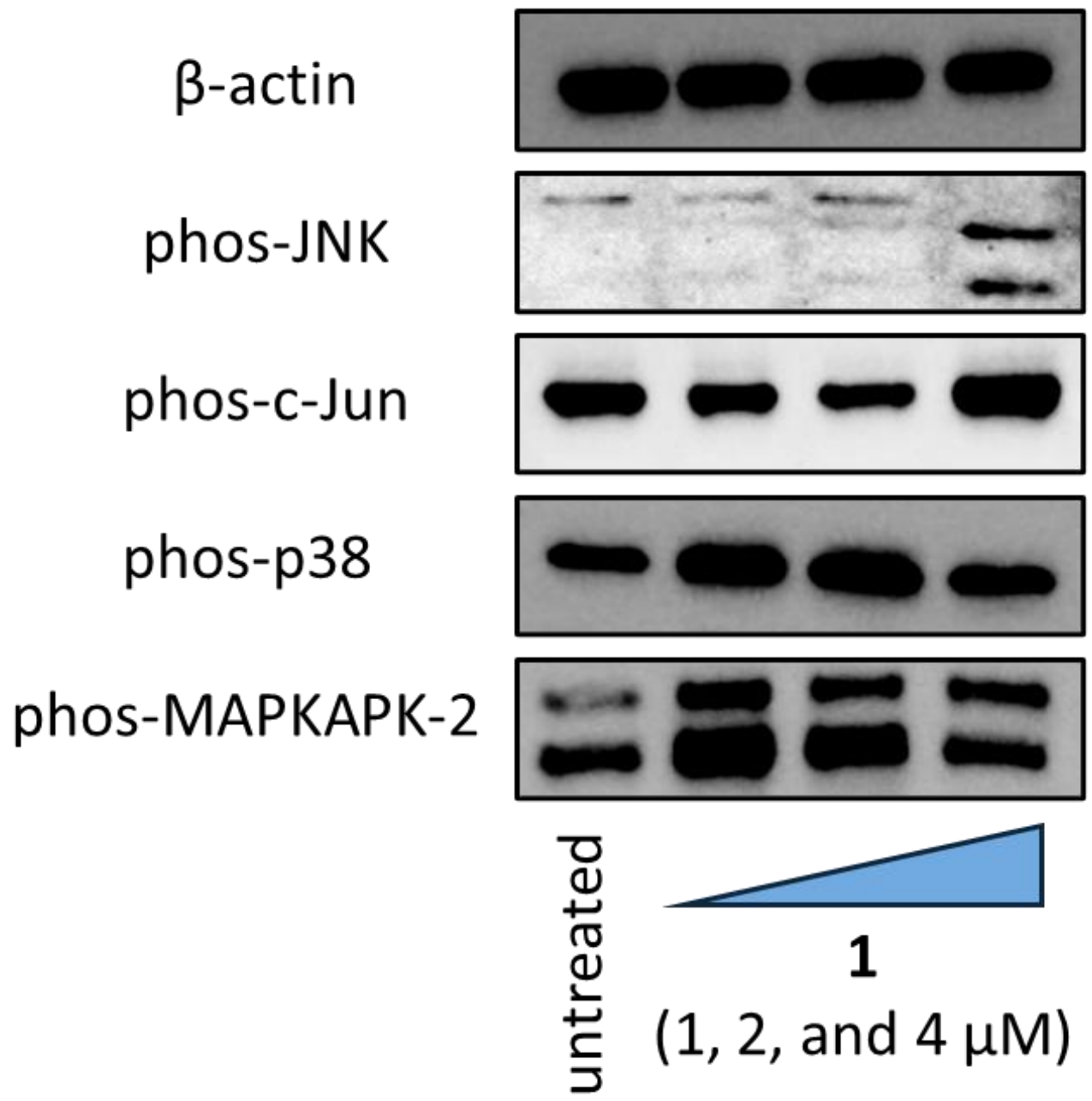

Figure S38. Immunoblotting analysis of proteins related to the JNK and p38 pathways. Protein expression in HMLER-shEcad cells following treatment with **1** (1-4 μM after 24 h incubation).

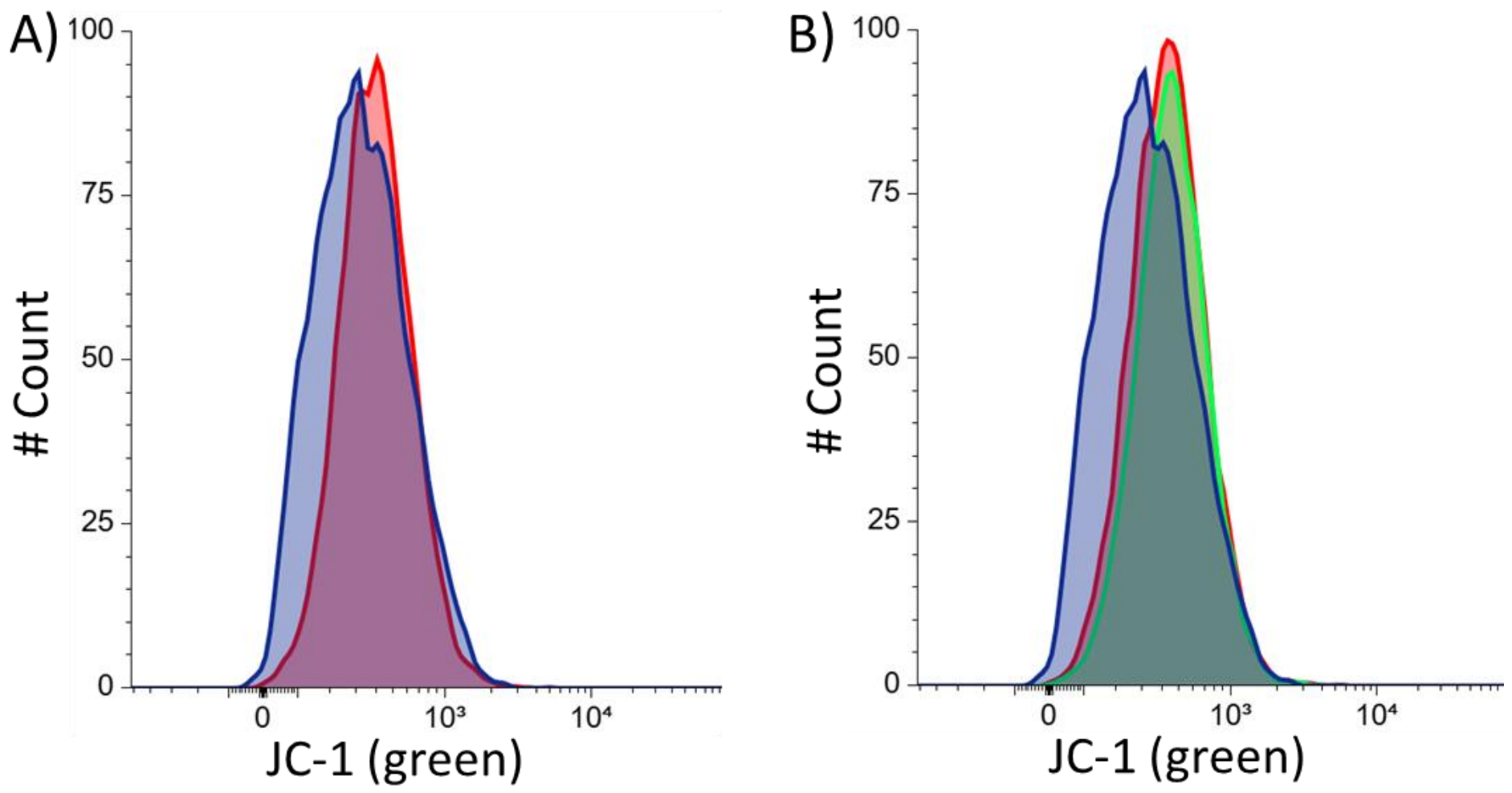

Figure S39. (A) Representative histograms displaying the green fluorescence emitted by JC-1 stained HMLER-shEcad cells (blue), and HMLER-shEcad cells treated with **1** (IC_{50} value for 24 h) (red). (B) Representative histograms displaying the green fluorescence emitted by JC-1 stained HMLER-shEcad cells (blue), and HMLER-shEcad cells treated with CCCP (1 μM or 5 μM for 24 h) (red and green).

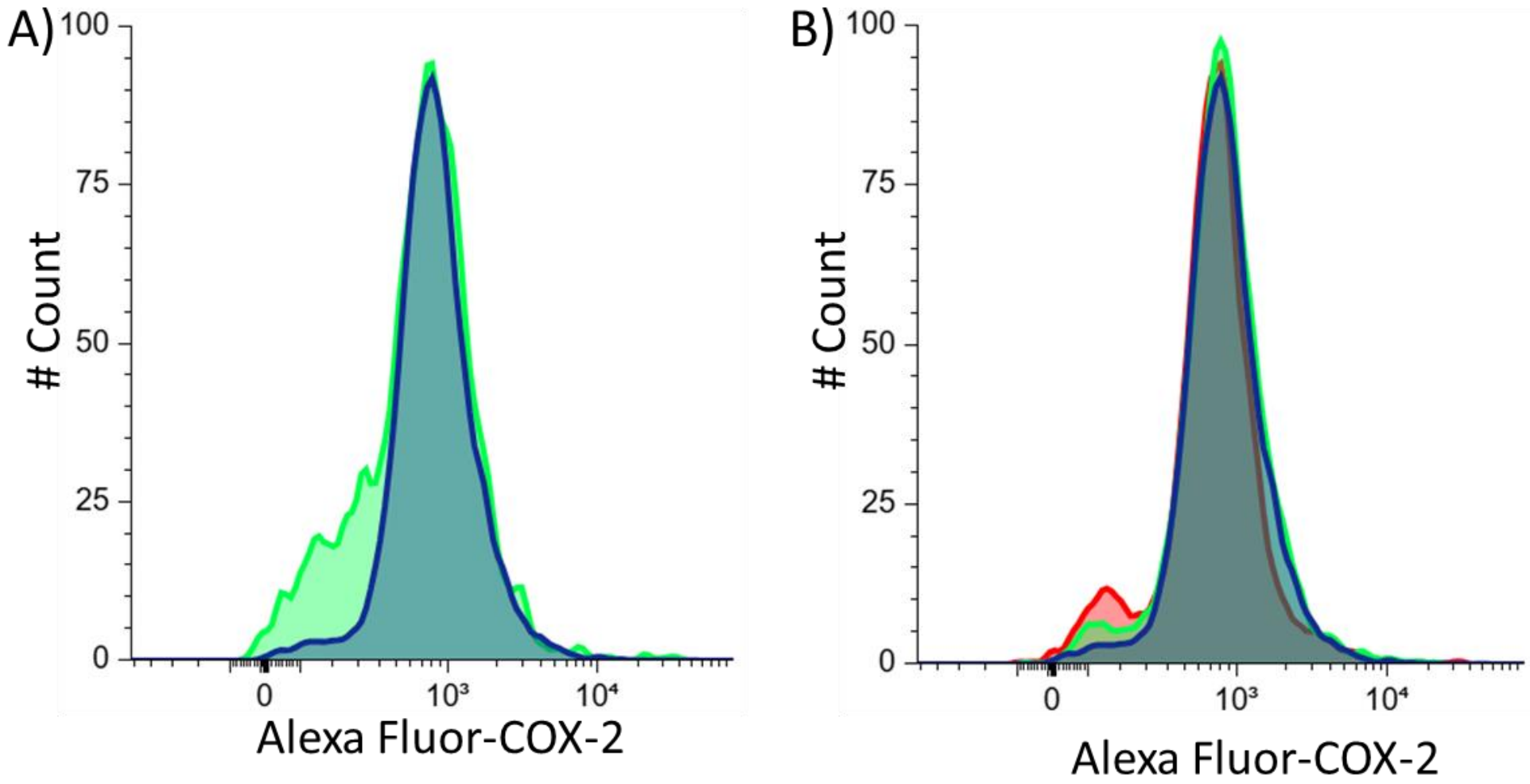

Figure S40. (A) Representative histograms displaying the green fluorescence emitted by anti-COX-2 Alexa Fluor 488 nm antibody-stained HMLER-shEcad cells treated with LPS (2.5 μg/mL) for 24 h, followed by 48 h in untreated media (blue) or media containing **1** (4 × IC_{50} value, green). (B) Representative histograms displaying the green fluorescence emitted by anti-COX-2 Alexa Fluor 488 nm antibody-stained HMLER-shEcad cells treated with LPS (2.5 μg/mL) for 24 h, followed by 48 h in untreated media (blue) or media containing diflunisal (20 or 40 μM, green and red).

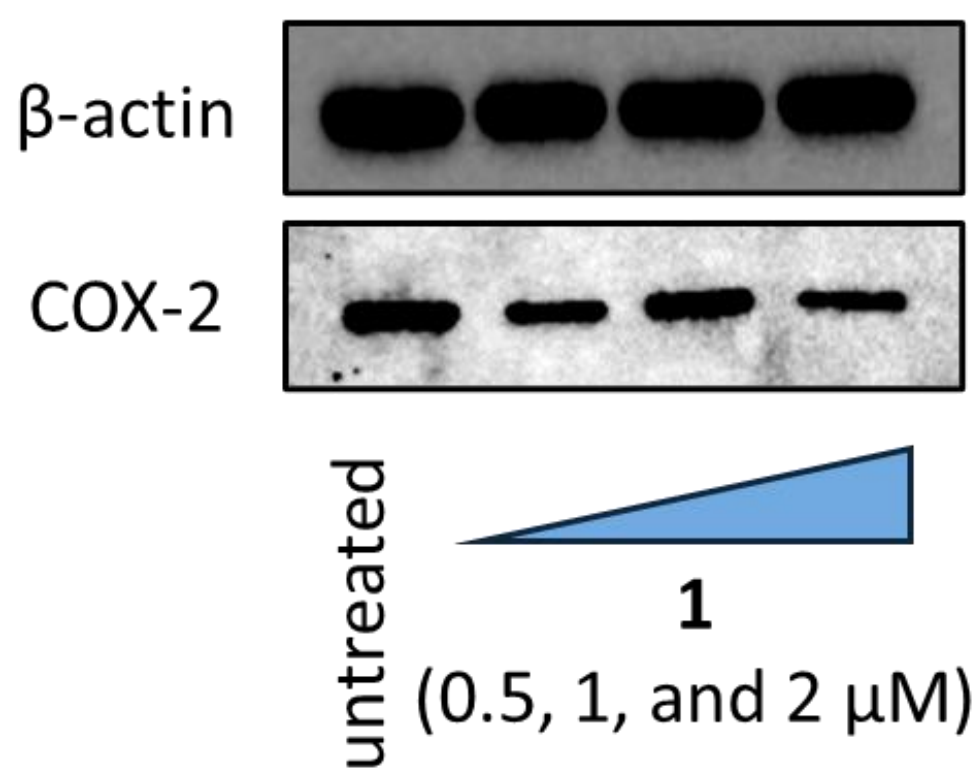

Figure S41. Immunoblotting analysis of the COX-2 protein. Protein expression in HMLER-shEcad cells following treatment with **1** (0.5-2 μM after 72 h incubation).

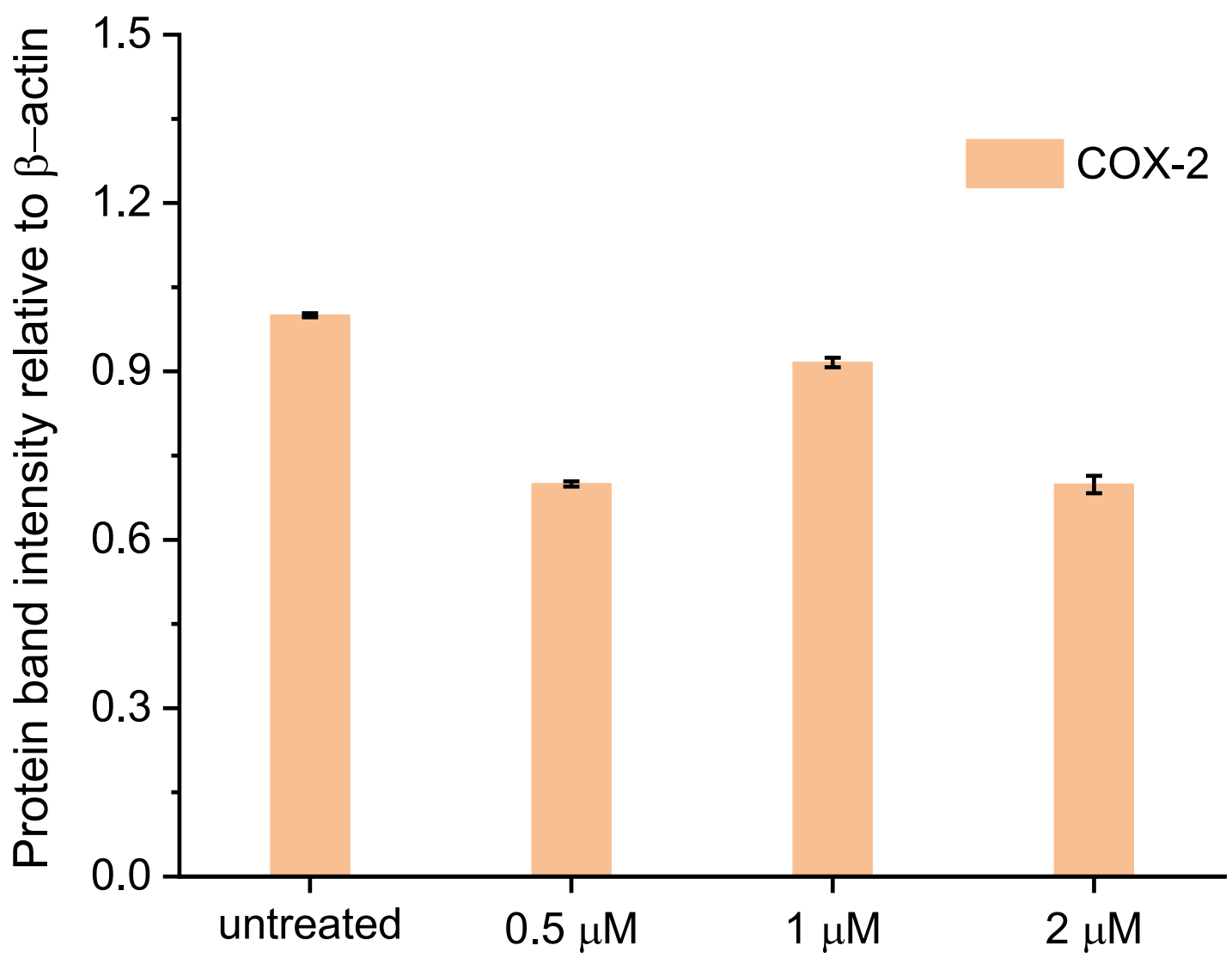

Figure S42. Protein band intensities corresponding to the immunoblotting analysis shown in Figure S41.

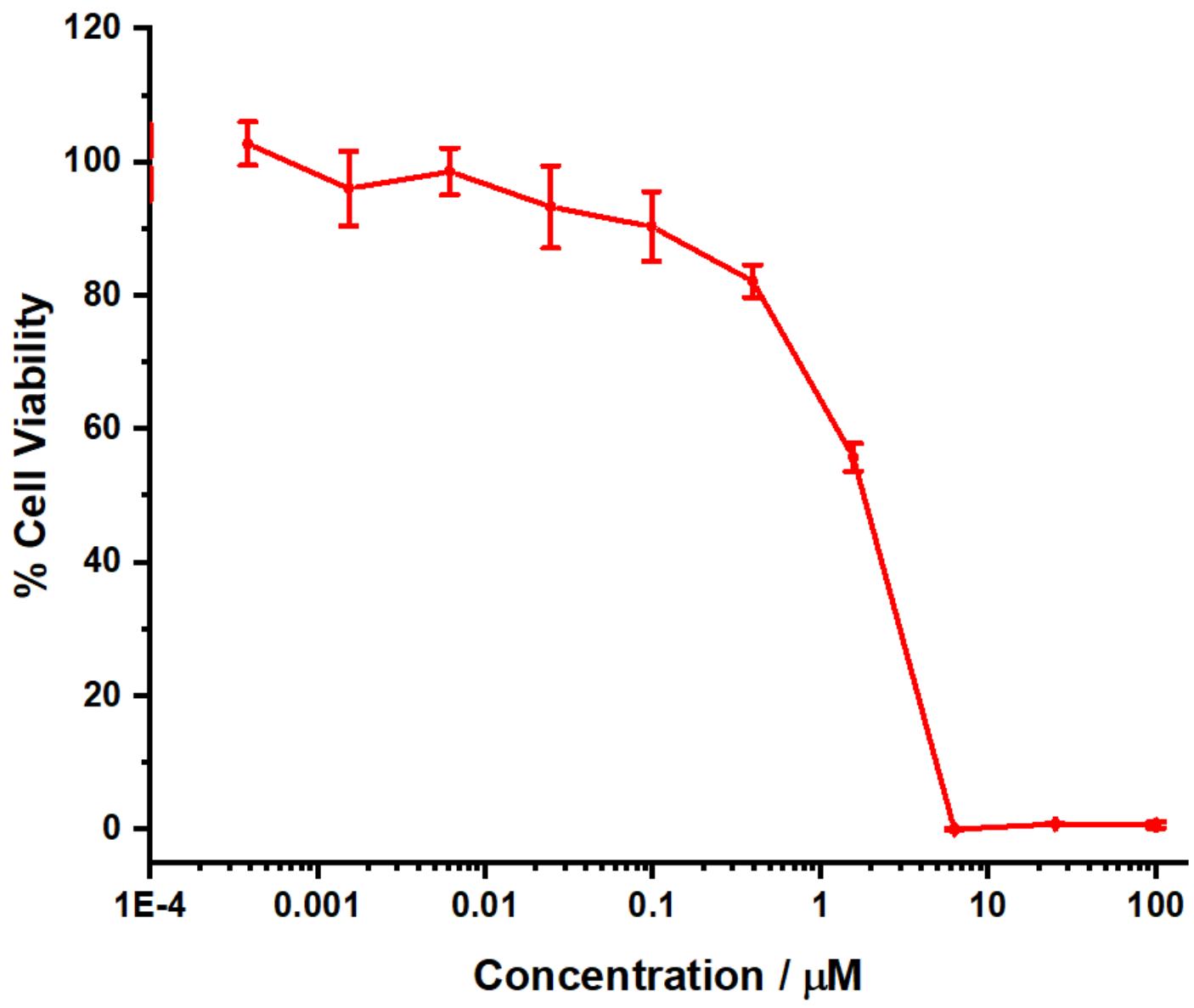

Figure S43. Representative dose-response curves for the treatment of HMLER-shEcad cells pre-treated with lipopolysaccharide (LPS) (2.5 μg/mL for 24 h) and with **1** after 72 h incubation. Error bars represent standard deviations.

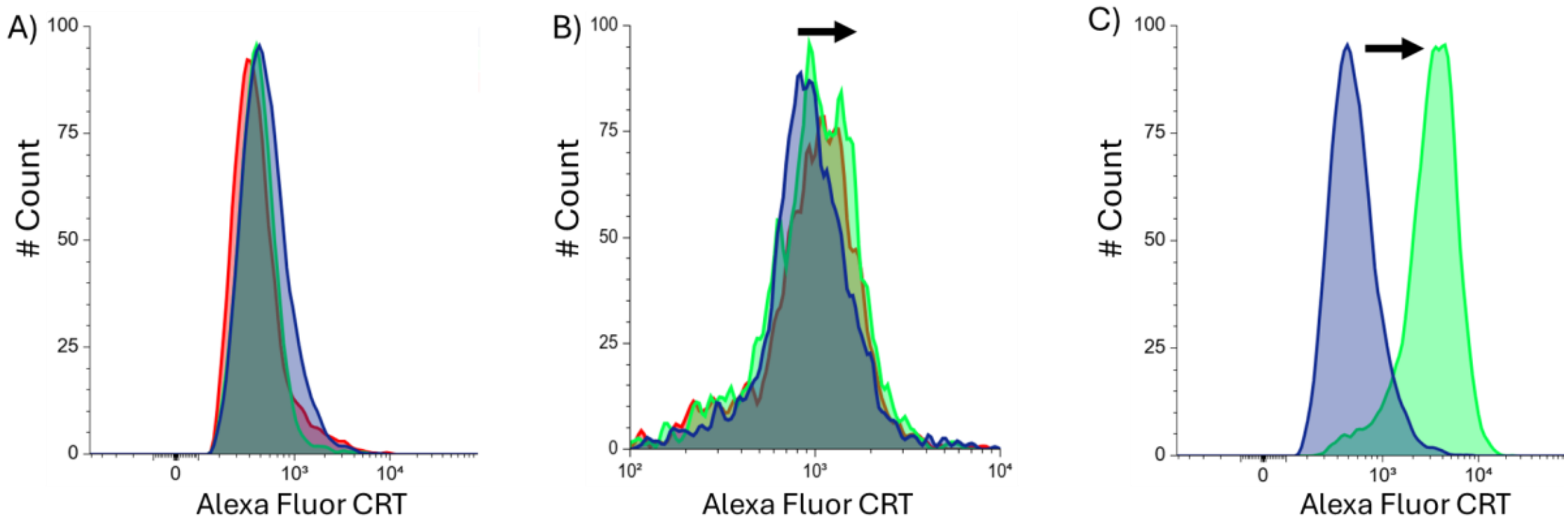

Figure S44. (A) Representative histograms displaying the green fluorescence emitted by anti-CRT Alexa Fluor 488 nm antibody-stained HMLER-shEcad cells untreated (blue) and treated with **1** (2 × IC_{50} value) (green) or **1** (4 × IC_{50} value) (red) for 24 h. (B) Representative histograms displaying the green fluorescence emitted by anti-CRT Alexa Fluor 488 nm antibody-stained HMLER-shEcad cells untreated (blue) and treated with **1** (2 × IC_{50} value) (green) or **1** (4 × IC_{50} value) (red) for 6 h. (C) Representative histograms displaying the green fluorescence emitted by anti-CRT Alexa Fluor 488 nm antibody-stained HMLER-shEcad cells untreated (blue) and treated with cisplatin (150 μM) and thapsigargin (7 μM) (green) for 24 h.

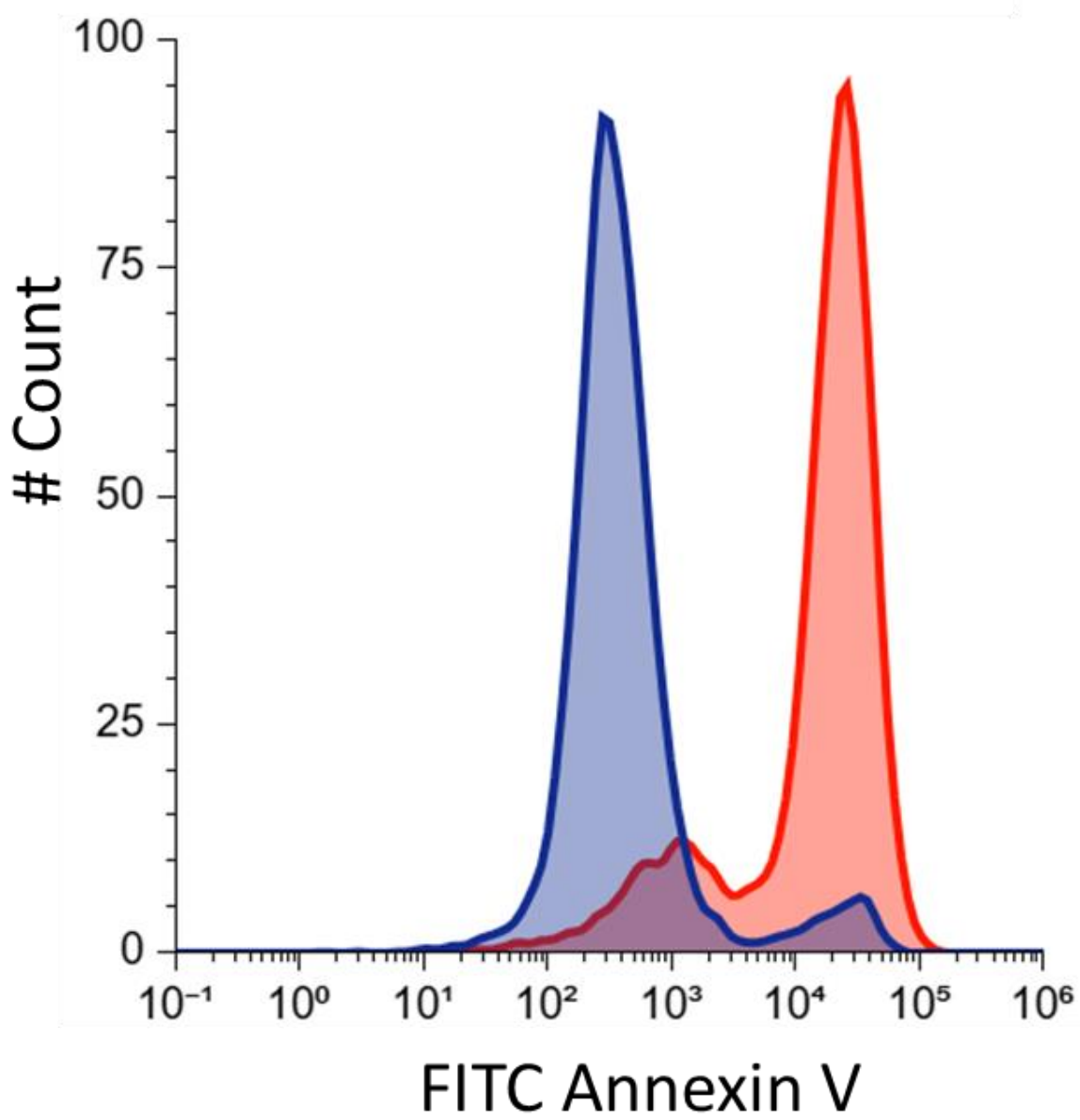

Figure S45. Representative histograms displaying the green fluorescence emitted by FITC Annexin V-stained HMLER-shEcad cells untreated (blue) and treated with cisplatin (25 μM) (red) for 72 h.

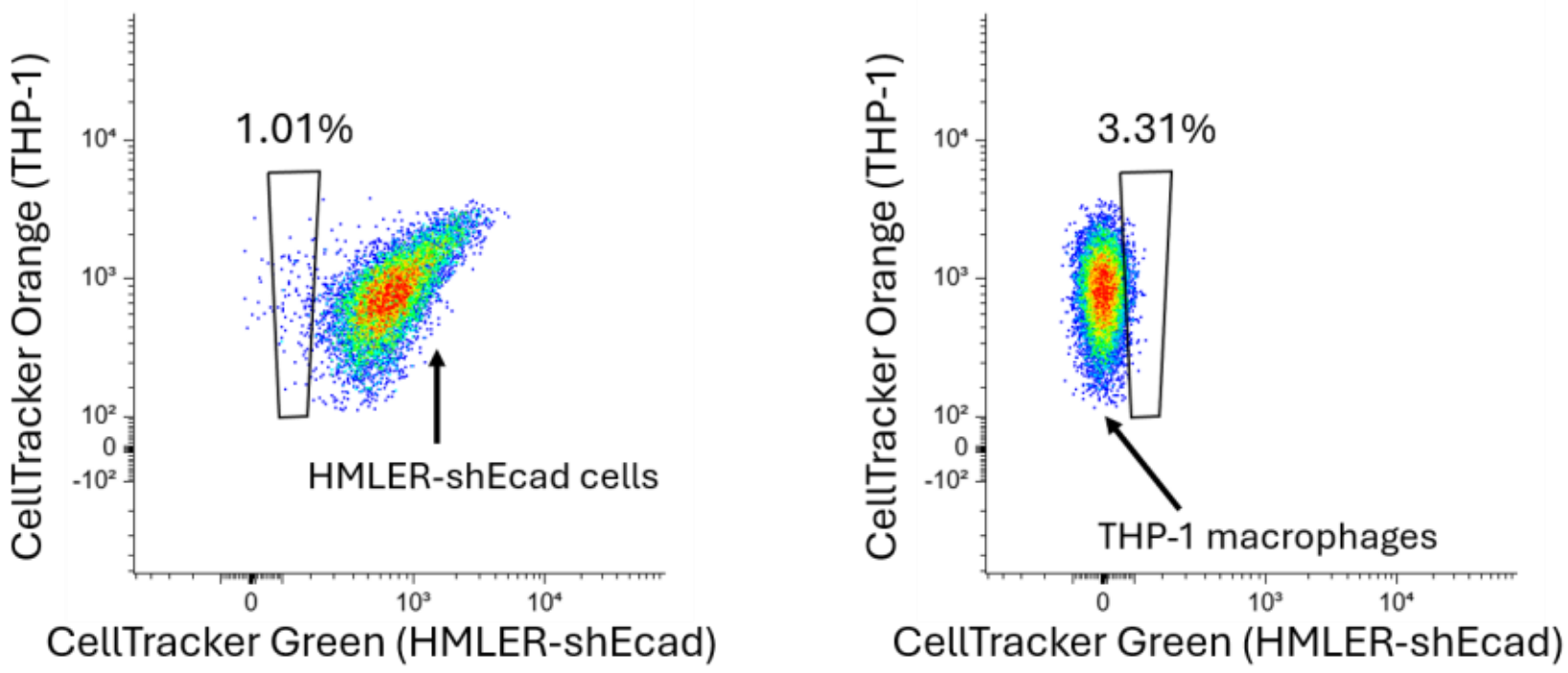

Figure S46. Representative two-dimensional scatter plots of CellTracker Green-stained HMLER-shEcad cells and CellTracker Orange-stained THP-1 macrophages.

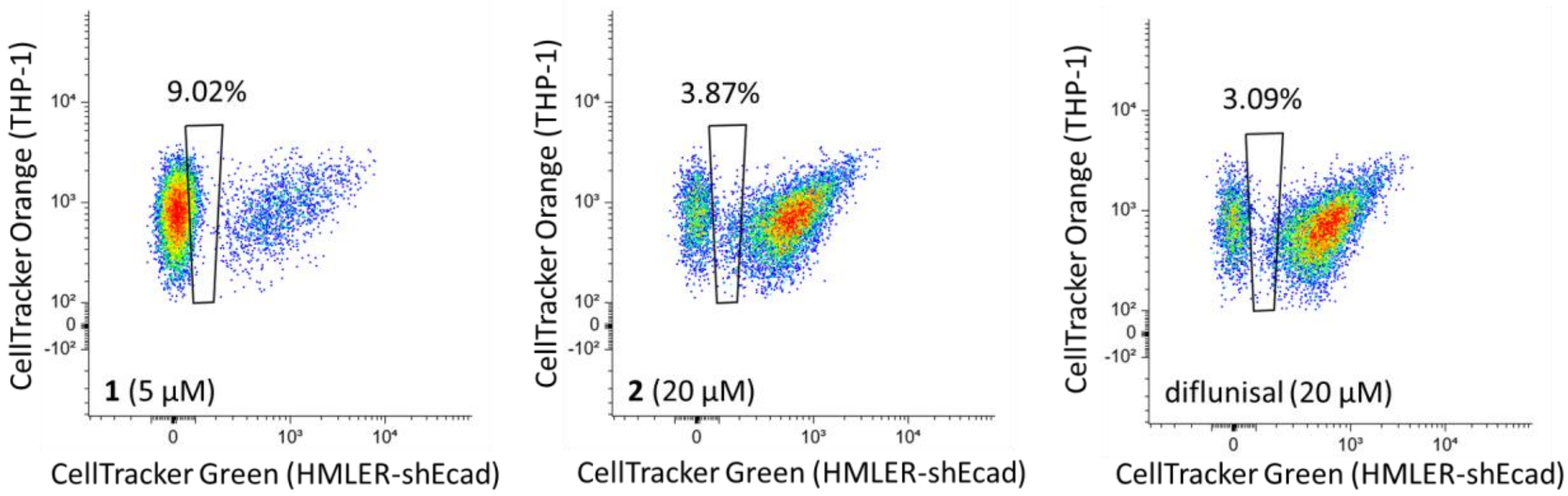

Figure S47. Representative two-dimensional scatter plots of CellTracker Green-stained HMLER-shEcad cells untreated and treated with **1** (5 μM) or **2** (20 μM) or diflunisal (20 μM) for 24 h and then co-cultured with CellTracker Orange-stained THP-1 macrophages for 2 h.

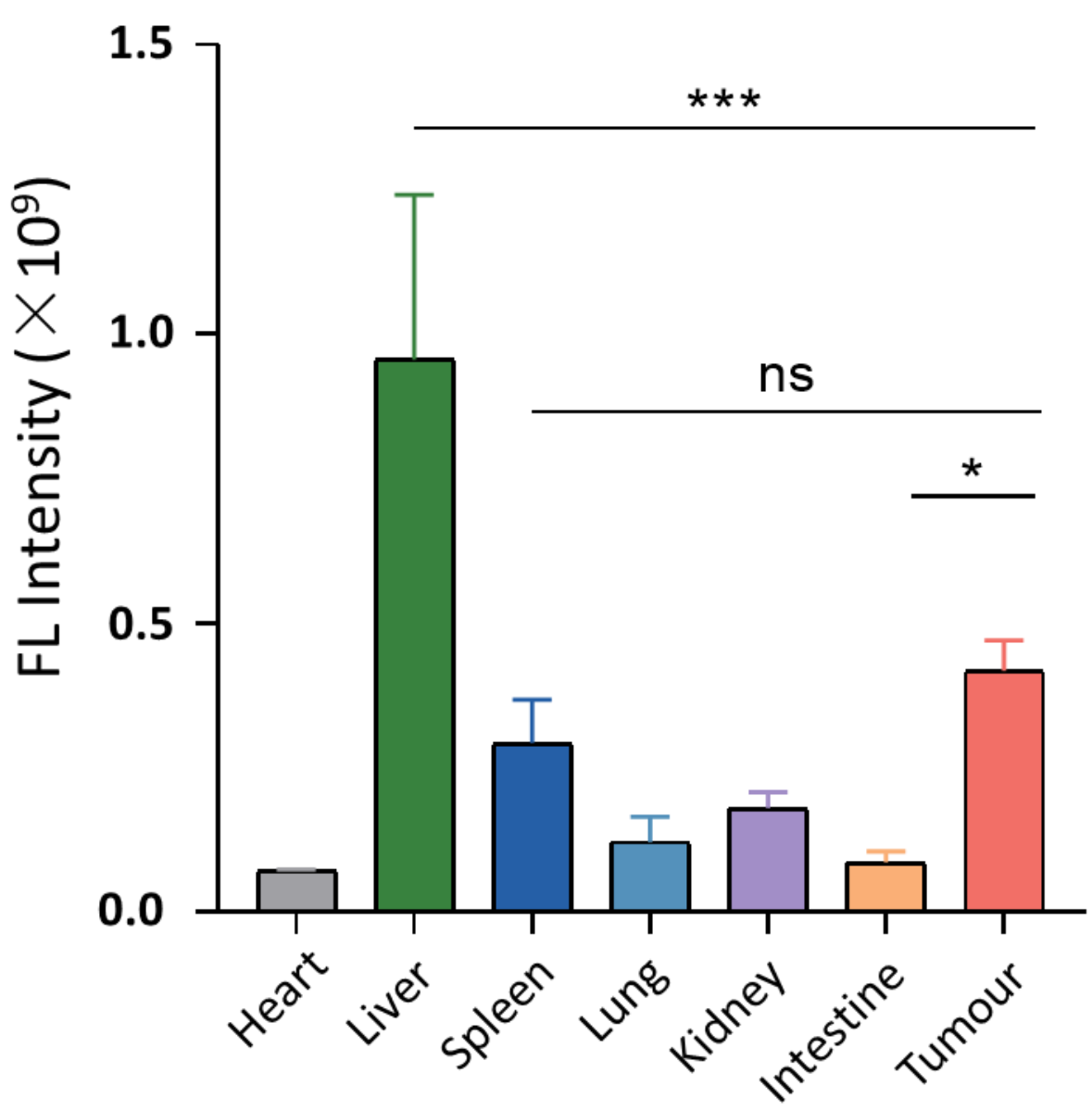

Figure S48. Biodistribution of **NP-1@Cy7.5** in BALB/c mice (n=5) bearing 4T1 breast tumours 3 days post intravenous administration.

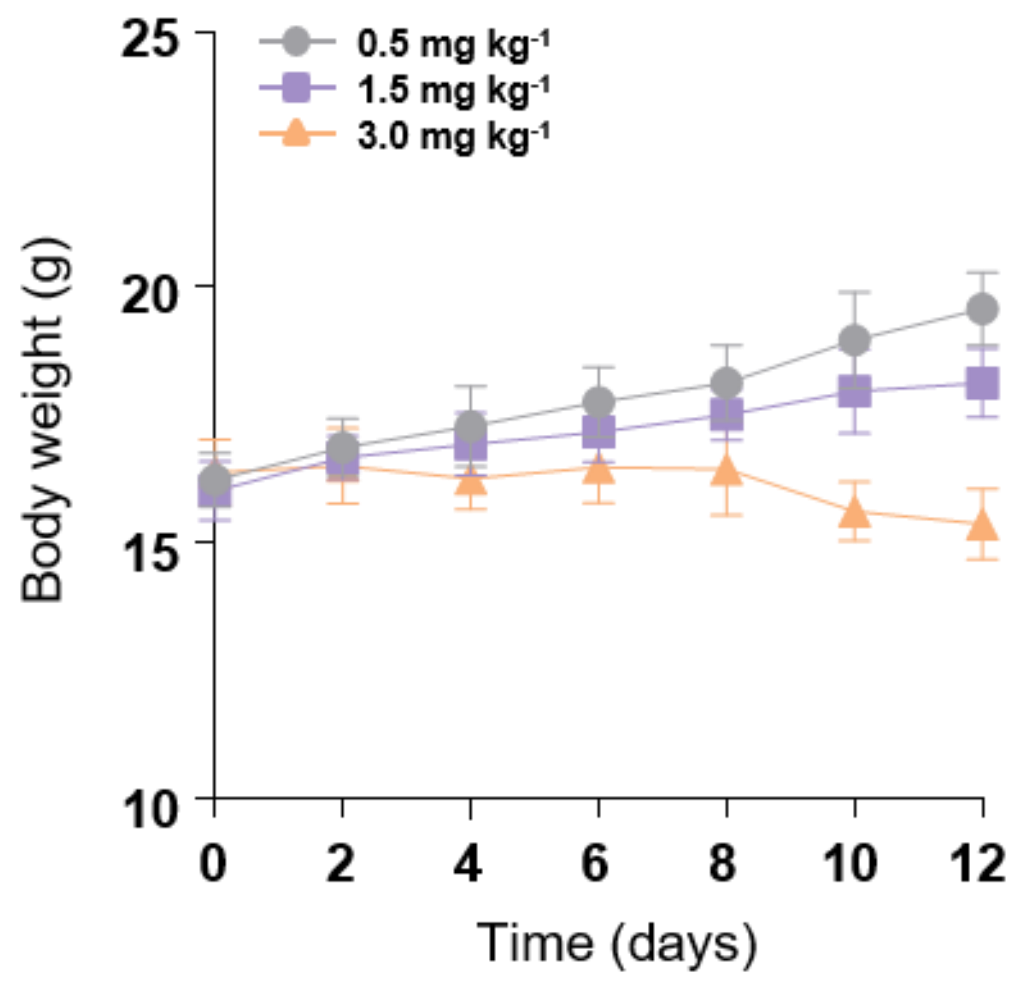

Figure S49. The change in body weight of BALB/c mice (n=6) upon intravenous administration of various doses of **NP-1** (0.5-3 mg kg^{-1}) over 12 days.

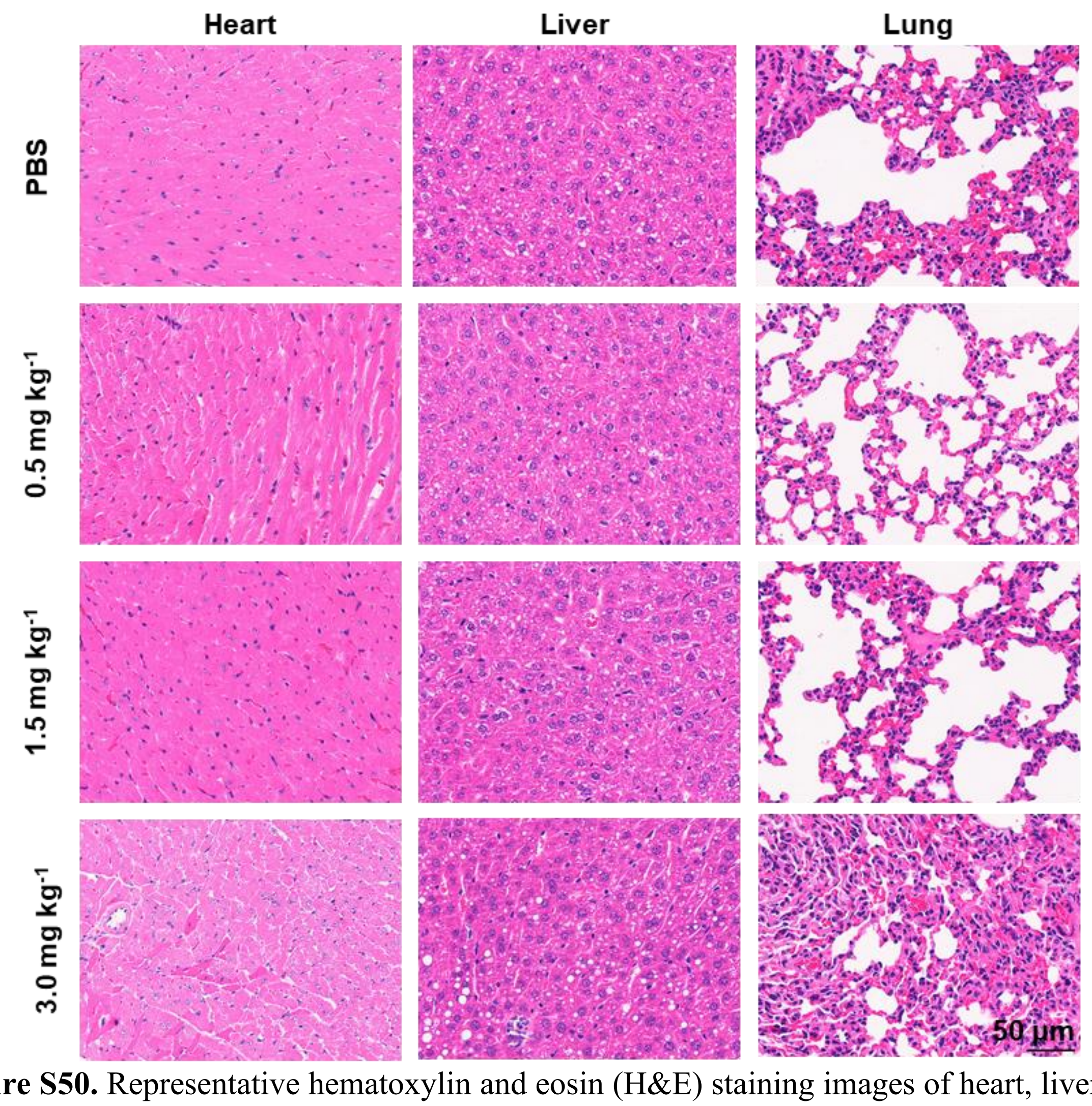

Figure S50. Representative hematoxylin and eosin (H&E) staining images of heart, liver, and lung tissue taken from BALB/c mice upon intravenous administration of various doses of **NP-1** (0.5-3 mg kg^{-1}) or the vehicle (PBS) over 12 days.

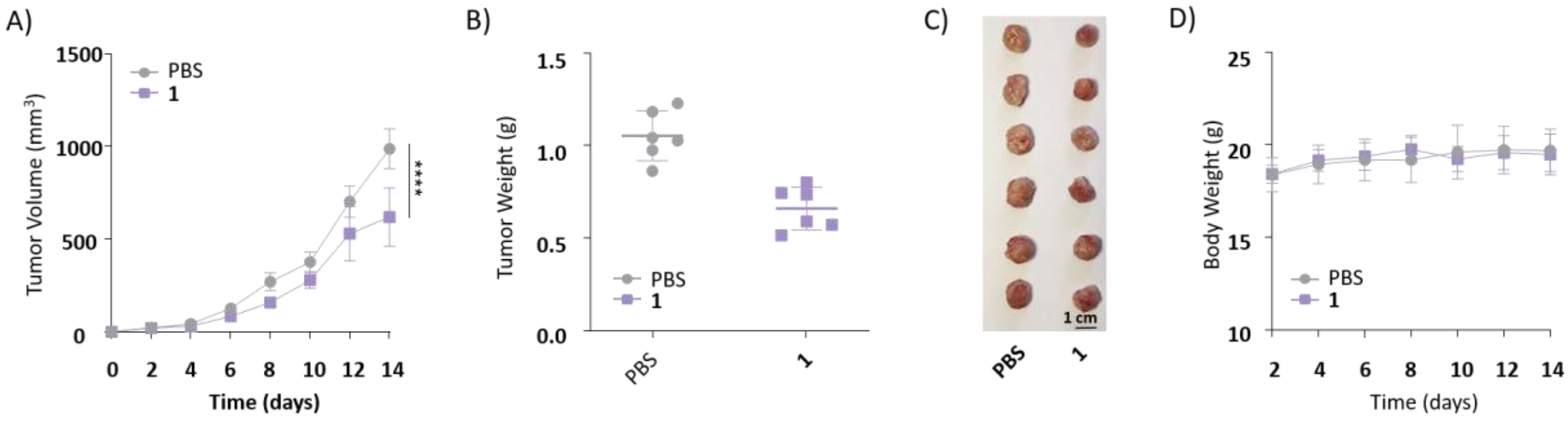

Figure S51. (A) Averaged tumour growth inhibition curves of 4T1 tumour-bearing mice (n = 6 mice), following intravenous administration of the vehicle (PBS) or **1** (1.5 mg Co kg^{-1}). The data is represented as the mean ± SD (n = 6 mice, ****$p < 0.0001$, one-way analysis of variance (ANOVA) with Tukey's multiple comparisons test). (B) Excised tumour weight after 14 days upon different treatments. (C) Images of excised tumours from 4T1 tumour-bearing mice after 14 days upon different treatments. (D) Body weight of 4T1 tumour-bearing mice during the efficacy study lasting 14 days.

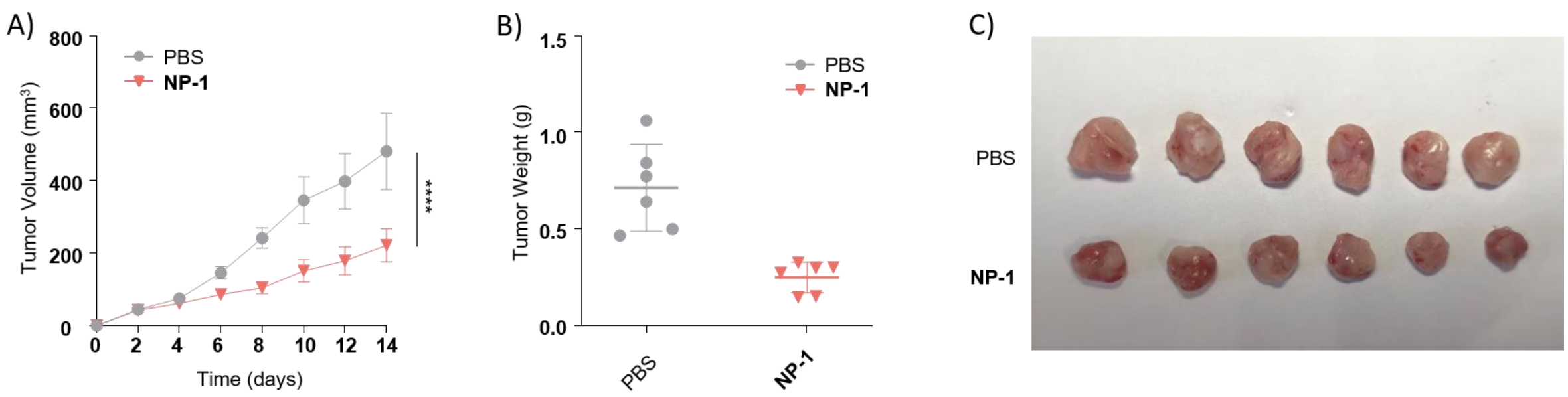

Figure S52. (A) Average tumour growth inhibition curves of secondary 4T1 tumours in mice (n = 6 mice) following primary tumour establishment by 4T1 cell inoculation, intravenous administration of either vehicle (PBS) or **NP-1** (1.5 mg Co kg^{-1}), surgical resection of the primary tumours after the treatment period, and subsequent rechallenge with a second inoculation of 4T1 cells. The data is represented as the mean ± SD (n = 6 mice, ****$p < 0.0001$, one-way analysis of variance (ANOVA) with Tukey's multiple comparisons test). (B) Excised secondary tumour weight measured 14 days after the second inoculation of 4T1 cells. (C) Images of excised secondary tumours collected from 4T1 tumour-bearing mice 14 days following rechallenge with a second inoculation of 4T1 cells.

<u>Uncropped images of the blots in the manuscript and SI</u>

Figure 4A

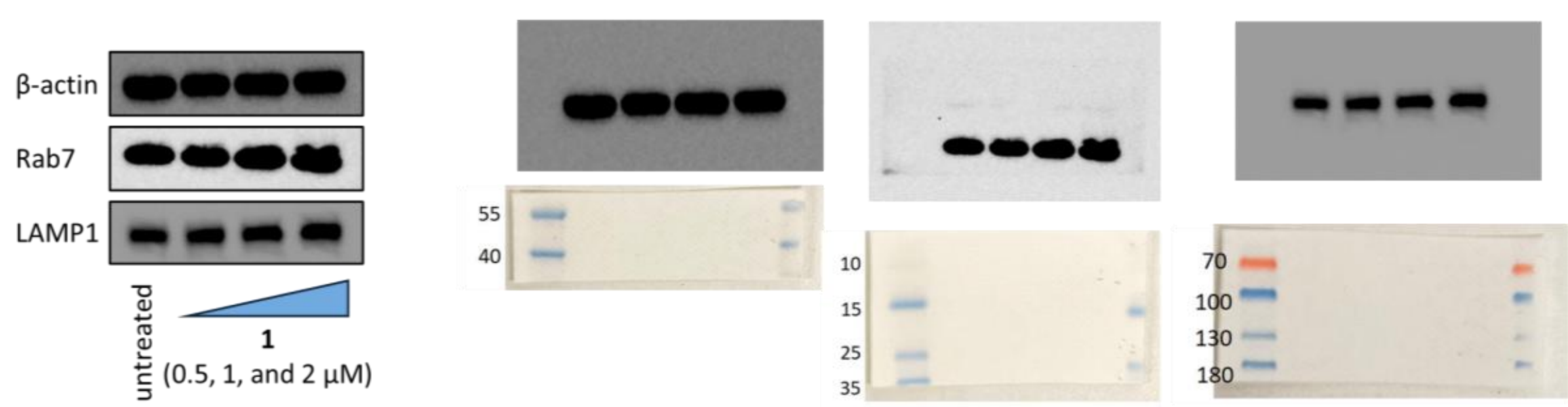

Figure 5B

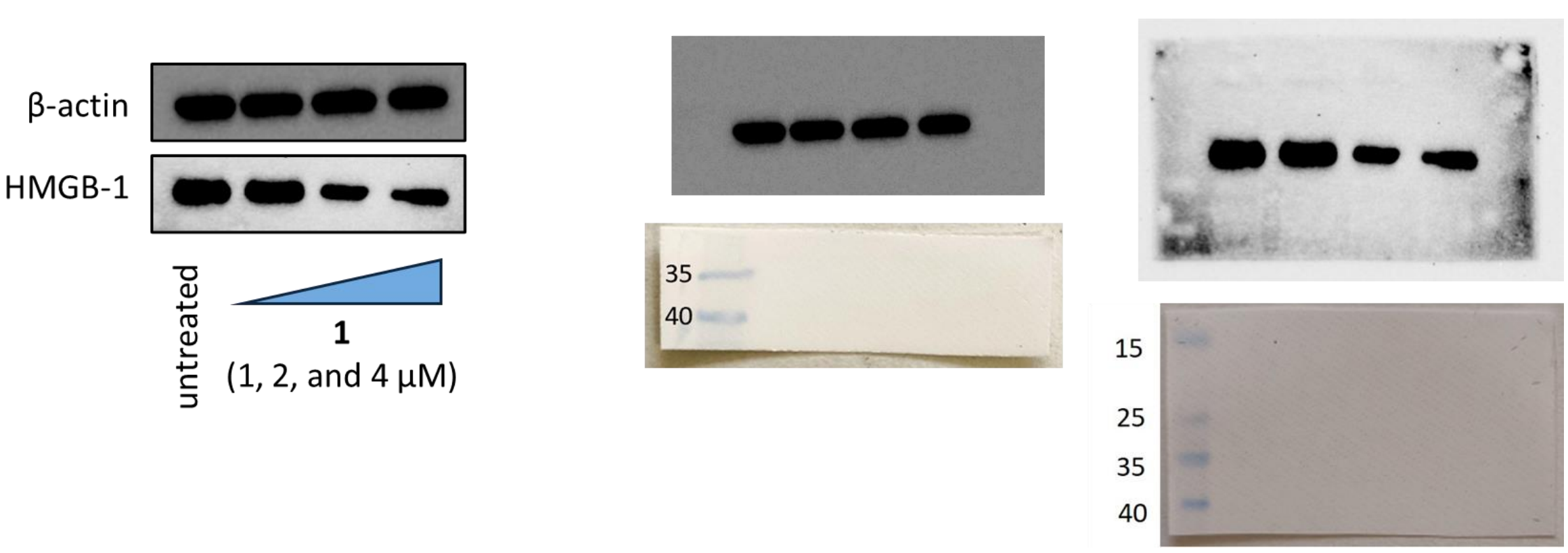

Figure S38

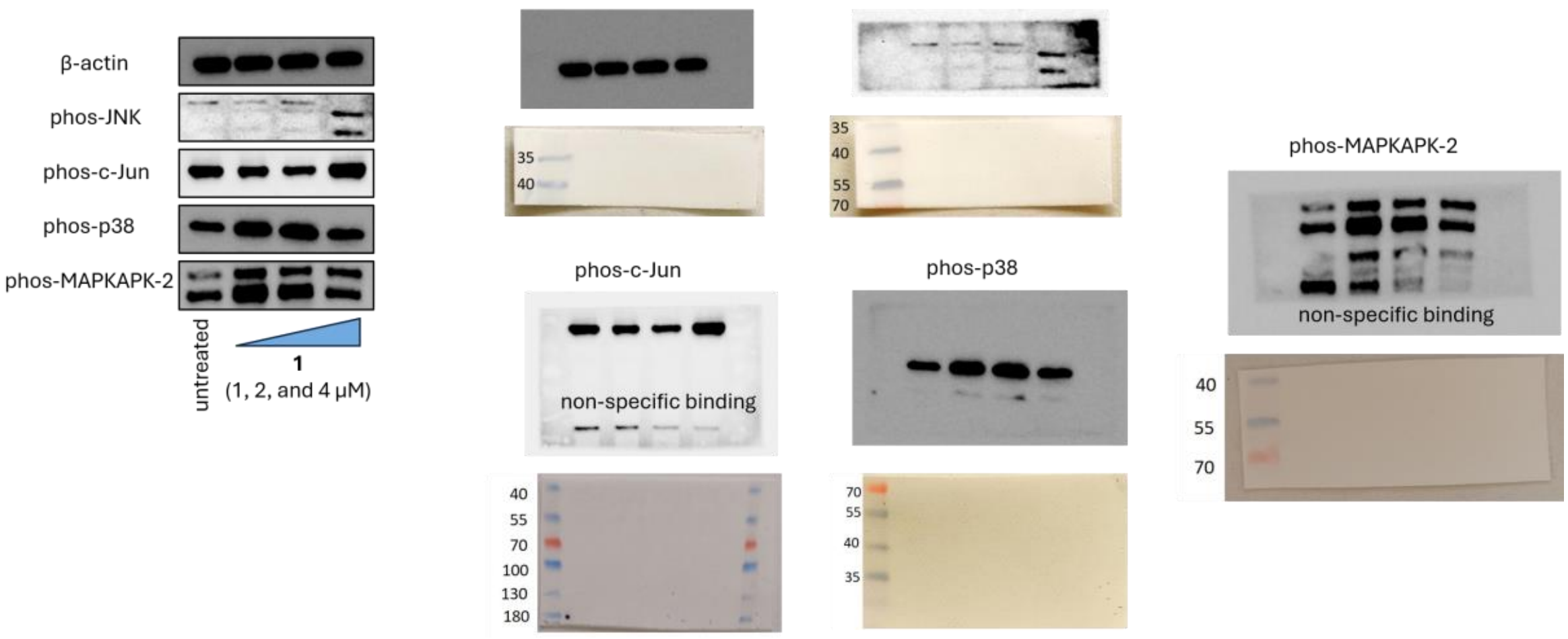

Figure S41

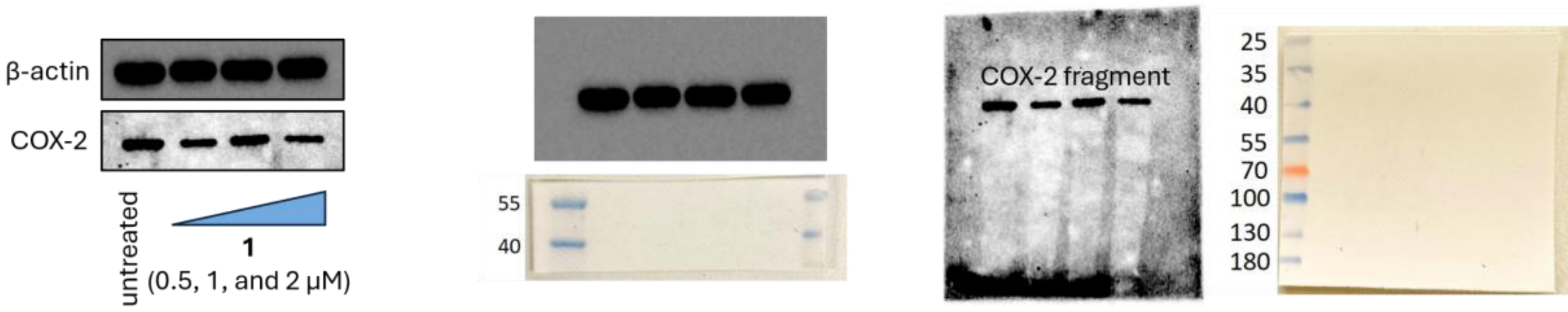

References

[1] B. Bosnich, C. K. Poon, M. L. Tobe, *Inorg. Chem.* **1965**, *4*, 1102-1108.

[2] G. M. Sheldrick, Program for Area Detector Absorption Correction, Institute for Inorganic Chemistry; University of Göttingen: Göttingen, Germany, **1996**.

[3] G. Sheldrick, *Acta Cryst.* **2008**, *A64*, 112-122.

[4] G. M. Sheldrick, *Acta Crystallogr. Sect. C* **2015**, *71*, 3-8.

[5] O. V. Dolomanov, L. J. Bourhis, R. J. Gildea, J. A. K. Howard, H. Puschmann, *J Appl Crystallogr* **2009**, *42*, 339-341.

[6] S. Wang, Y. Zhang, W. Cong, J. Liu, Y. Zhang, H. Fan, Y. Xu, H. Lin, *J. Tradit. Chin. Med.* **2016**, *36*, 530-537.
